# Astrocytic SCAP deletion ameliorates diabetes-associated cognitive impairment by suppressing microglial neuroinflammation and lipid droplet accumulation via the LCN2–24p3R–mTOR axis

**DOI:** 10.1038/s41419-026-09019-y

**Published:** 2026-06-22

**Authors:** Kunyu Liu, Haoqiang Zhang, Wenwen Zhu, Tong Niu, Yonglian Hu, Haiping Zhou, Ruoyu Sun, Junru Zhou, Shaohua Wang, Yang Yuan

**Affiliations:** 1 Department of Endocrinology, Affiliated Zhongda Hospital of Southeast University, Nanjing, China; 2 School of Medicine, Southeast University, Nanjing, China; 3 Department of Endocrinology and Metabolism, Center for Leading Medicine and Advanced Technologies of IHM, The First Affiliated Hospital of USTC, Division of Life Sciences and Medicine, University of Science and Technology of China, Hefei, China; 4 Huzhou Central Hospital, Affiliated Central Hospital of Huzhou University, Affiliated Huzhou Hospital, Zhejiang University School of Medicine, Huzhou, China; 5 Department of Endocrinology, The Affiliated Suzhou Hospital of Nanjing Medical University, Suzhou Municipal Hospital, Suzhou, Jiangsu China; 6 The First People’s Hospital of Bengbu, Bengbu, China; 7 Department of Endocrinology, Tongren Hospital, Shanghai Jiao Tong University School of Medicine, Shanghai, China

**Keywords:** Diabetes complications, Alzheimer's disease

## Abstract

Diabetes-associated cognitive impairment (DACI) is a prevalent and debilitating complication of type 2 diabetes, yet the mechanisms linking metabolic dysregulation to neuroinflammation and cognitive decline remain incompletely understood. In this study, we identify astrocytic SCAP (SREBP cleavage-activating protein) as a crucial regulator of glial metabolic–inflammatory crosstalk in DACI. Using a high-fat diet-induced mouse model of diabetes, we demonstrate that astrocytic SCAP expression is significantly upregulated, whereas astrocyte-specific SCAP deletion alleviates cognitive deficits, reduces microglial activation, and attenuates lipid droplet accumulation in the hippocampus. Transcriptomic analysis of SCAP-deficient mice identified lipocalin-2 (LCN2) as a prominent downstream candidate associated with inflammatory regulation. Mechanistically, astrocytic SCAP promotes NF-κB–dependent LCN2 expression, leading to LCN2-dependent paracrine activation of microglial 24p3R–mTOR signaling and consequent microglial inflammatory activation and lipid droplet accumulation. Consistently, pharmacological inhibition of mTOR or antibody-mediated blockade of LCN2 in vivo significantly ameliorated hippocampal neuroinflammation, neuronal injury, and cognitive decline in diabetic mice. These findings reveal a novel SCAP–LCN2–24p3R–mTOR signaling axis that links astrocytic lipid sensing to microglial inflammatory and metabolic dysfunction, providing a mechanistic framework for metabolic-inflammatory coupling in DACI and highlighting this pathway as a potential therapeutic target for DACI.

## Introduction

Type 2 diabetes mellitus (T2DM) is a common chronic metabolic disease, and its global prevalence is expected to increase to 783 million by 2045 [1]. Among the central nervous system (CNS) complications associated with T2DM, cognitive impairment is one of the most common, often presenting with structural brain abnormalities and deficits in learning and memory. Diabetes-associated cognitive impairment (DACI) not only impairs patients’ cognitive function but also compromises their ability to perform effective glycemic self-management, thereby increasing the risk of severe metabolic complications, such as hypoglycemia, hyperosmolar hyperglycemic state, and diabetic ketoacidosis. Currently, treatment strategies for DACI primarily include glycemic control, dietary modifications, lifestyle interventions, and neurotrophic support. However, clinical evidence suggests that glycemic control alone has limited efficacy in improving cognitive outcomes [2], underscoring the urgent need to elucidate the underlying pathophysiological mechanisms of DACI and identify novel therapeutic targets.

Emerging evidence suggests that the interplay between metabolic dysregulation and neuroinflammation plays a central role in the pathogenesis of DACI [3]. Among the cellular mediators involved, dysfunctional glial cells—particularly, the aberrant activation of astrocytes and microglia—have been identified as key contributors to DACI-related neuropathology [4]. Astrocytes and microglia not only fulfill distinct immunological functions but also engage in bidirectional communication following CNS insults, thereby amplifying neuroinflammatory responses. Microglia act as resident immune sentinels, rapidly responding to neuronal damage through the release of pro-inflammatory cytokines and chemokines, which, in turn, influence astrocytic activation. Conversely, reactive astrocytes can modulate microglia phenotypes and function [5]. Microglia and astrocytes exhibit extensive crosstalk, and this bidirectional communication is a fundamental feature and/or cause of chronic glial reactivity found in most neurodegenerative conditions [6–8].

Sterol regulatory element-binding protein (SREBP) cleavage-activating protein (SCAP) is an essential regulator of cellular lipid homeostasis, functioning as an obligate chaperone for SREBP to coordinate cholesterol and fatty acid synthesis. Beyond its canonical metabolic role, dysregulated SCAP activity has been linked to heightened inflammatory responses in multiple peripheral metabolic disorders [9–12]. In the CNS, astrocyte-specific SCAP deficiency severely disrupts lipid synthesis and impairs hippocampal synapse formation and function [13], highlighting its importance in brain homeostasis. Our previous studies further revealed that SCAP participates in neuroinflammatory signaling through cell-type–specific mechanisms: in microglia, SCAP accumulation within the Golgi apparatus enhances NLRP3 inflammasome activation, thereby exacerbating neuroinflammation and cognitive impairment in diabetic mice [14]; in astrocytes, SCAP facilitates the recruitment of IκBα to the Golgi, promoting NF-κB nuclear translocation and complement C3 transcription, ultimately contributing to neuronal dysfunction during DACI progression [15]. Collectively, these findings identify SCAP as a noncanonical regulator of neuroinflammation, extending its functional repertoire beyond lipid metabolic regulation.

Despite growing evidence that SCAP exerts cell-type–specific effects in the CNS, it remains unclear whether astrocytic SCAP contributes to diabetic neuroinflammation by orchestrating astrocyte–microglia crosstalk. Owing to the blood–brain barrier, cholesterol within the CNS is largely synthesized locally, with astrocytes serving as the principal source and acting as key immunometabolic regulators that integrate metabolic and inflammatory signaling to shape interglial communication [16, 17]. In the present study, using a high-fat diet (HFD)–induced T2DM mouse model, we identify a previously unrecognized astrocyte-to-microglia signaling axis driven by astrocytic SCAP that promotes microglial inflammatory activation and metabolic reprogramming, thereby contributing to the pathogenesis of DACI.

## Materials and methods

### Animal models

Astrocyte-specific SCAP conditional knockout (SCAP cKO) mice were generated by crossing SCAP floxed mice on a C57BL/6J background with Aldh1l1-CreERT2 transgenic mice (Jackson Laboratory, USA), which express tamoxifen-inducible Cre recombinase under the control of the Aldh1l1 promoter. Tamoxifen (Sigma-Aldrich, T5648) was dissolved in corn oil and administered intraperitoneally at a dose of 75 mg/kg once daily for 5 consecutive days at 6 weeks of age. Cre-negative SCAP flox/flox littermates were used as controls. All mice were housed under standard conditions with a 12-h light/dark cycle and had ad libitum access to food and water. Only male mice were used in this study.

To induce T2DM, mice were fed a HFD (60% fat, 20% protein, 20% carbohydrate; D12492, Research Diets) for 24 weeks, while control mice received a normal chow diet (NCD; 10% fat, 20% protein, 70% carbohydrate; D12450B, Research Diets). Based on genotype and dietary intervention, mice were divided into four experimental groups: NCD-fed SCAP fl/fl (NCD fl/fl), NCD-fed SCAP cKO (NCD cKO), HFD-fed SCAP fl/fl (HFD fl/fl), and HFD-fed SCAP cKO (HFD cKO). These group designations are used throughout the manuscript for simplicity.

For in vivo intervention experiments, HFD-fed mice were randomly assigned to receive either rapamycin (RAPA) or systemic anti-LCN2 antibody treatment. RAPA was prepared as previously described and administered via intraperitoneal injection at a dose of 2 mg/kg every other day for 14 days, with vehicle-treated mice serving as controls [18]. In parallel, a separate cohort of HFD-fed mice received daily intraperitoneal injections of a polyclonal anti-LCN2 antibody (Invitrogen, PA5-79590) for 14 consecutive days, based on previously published protocols [19]. Control animals received species-matched IgG at the same dose and schedule.

Investigators were blinded to genotype and treatment during behavioral testing and data analysis whenever feasible. All animal procedures were conducted in accordance with the ARRIVE guidelines and institutional regulations for animal care and use, and were approved by the Institutional Animal Care and Use Committee of Zhongda Hospital, Southeast University, China (Approval No. 20210301022).

### Glucose tolerance test (GTT)

Mice were fasted for 12 h before testing. Baseline blood glucose levels were measured immediately prior to intraperitoneal injection of glucose (2 g/kg body weight). Blood samples were collected from the tail vein at 15, 30, 60, 90, and 120 min after glucose administration. Glucose concentrations were determined using a handheld glucometer (Accu-Chek®, Roche Diagnostics, Basel, Switzerland), with each measurement performed in duplicate to ensure accuracy. The glucometer was calibrated according to the manufacturer’s instructions prior to use.

### Behavioral analysis

#### Open field test (OFT)

The OFT was conducted to assess anxiety-like behavior as previously described [20, 21]. Mice were placed in the center of a white PVC arena (50 × 50 × 50 cm) and allowed to explore freely for 5 min. Behavior was recorded using an overhead video tracking system. The time spent and the number of entries into the central zone (25 × 25 cm) were analyzed. The arena was cleaned with 75% ethanol between trials to remove olfactory cues.

#### Novel object recognition (NOR) test

Recognition memory was assessed using the NOR test as described previously [22]. During training, mice were placed in an arena containing two identical objects (A and B) and allowed to explore for 5 min. After a retention interval (1 or 24 h), one object was replaced with a novel object. Mice were then reintroduced into the arena for 5 min, and exploration times were recorded. The preference index (PI) was calculated as: PI = [time with novel / (time with novel + time with familiar)] × 100%.

#### Y-maze test

Short-term spatial memory was evaluated using the Y-maze test [23]. Mice were allowed to freely explore three visually distinct arms for 6 min. Arm entry sequence was recorded to calculate the spontaneous alternation rate (SAR): SAR = [number of alternations / (total entries − 2)] × 100%. Total arm entries and distance traveled were measured to assess locomotor activity.

#### Morris water maze (MWM)

Spatial learning and memory were assessed using the MWM [24]. Mice were trained for 5 days (4 trials per day) to locate a submerged platform in a circular pool (150 cm diameter) with visual cues. The water temperature was maintained at 21 ± 1°C and made opaque with non-toxic dye. Escape latency was recorded. On day 6, a probe trial was conducted with the platform removed. Mice were allowed to swim freely for 60 s, and platform crossings, path length, and time spent in the target quadrant were recorded using the tracking system.

### Tissue perfusion and sample collection

Following completion of behavioral testing, mice were deeply anesthetized with isoflurane and subjected to tissue collection. For histological analyses, mice were transcardially perfused with phosphate-buffered saline (PBS), followed by 4% paraformaldehyde (PFA). Brains were removed, post-fixed in 4% PFA for 12 h at 4 °C, and subsequently cryoprotected in 30% sucrose solution until they sank. Coronal brain sections were prepared using a cryostat for immunohistochemical and immunofluorescence analyses.

For molecular analyses, a separate cohort of mice was euthanized without perfusion. Bilateral hippocampal tissues were rapidly dissected on ice, snap-frozen in liquid nitrogen, and stored at −80 °C until further processing. Blood samples were collected at sacrifice from the orbital venous plexus into EDTA-coated tubes and centrifuged at 3000 × *g* for 10 min at 4 °C to obtain plasma, which was stored at −80 °C until analysis.

#### Metabolic parameter assays

Plasma levels of total cholesterol (TC), triglycerides (TG), low-density lipoprotein cholesterol (LDL-C), and high-density lipoprotein cholesterol (HDL-C) were measured using enzymatic commercial kits (Nanjing Jiancheng Bioengineering Institute, China) according to the manufacturer’s instructions, as previously described [25]. For tissue assays, hippocampal samples were homogenized in ice-cold PBS (1:9, w/v) and centrifuged to obtain supernatants for analysis. Hippocampal TC and TG levels were quantified using the same kits.

#### Isolation of microglia and astrocytes by magnetic-activated cell sorting (MACS)

Single-cell suspensions were prepared from adult mouse brains using the Brain Dissociation Kit (P) (Miltenyi Biotec, 130-095-942) in combination with a 30% Percoll gradient, as previously described with minor modifications [26]. Whole brains were used to ensure sufficient cell yield. Briefly, whole brains were rapidly collected, rinsed in Hank’s balanced salt solution (HBSS), and finely minced using a sterile scalpel. Tissue fragments were enzymatically dissociated at 37 °C for 15 min according to the manufacturer’s instructions. The resulting suspension was filtered through a 70 μm cell strainer to obtain a single-cell suspension. Following centrifugation, myelin and cellular debris were removed using a 30% Percoll gradient (800 × *g*, 12 min, 25 °C). The cell pellet was resuspended in PBS containing 0.5% fetal bovine serum (FBS), and cell concentration was adjusted to 1 × 10⁷ cells/mL. Microglia were isolated by positive magnetic selection using anti-CD11b microbeads (Miltenyi Biotec, 130-093-634). The CD11b⁻ flow-through fraction was then used for astrocyte isolation with anti-ACSA-2 microbeads (Miltenyi Biotec, 130-116-249). The purity of isolated microglia and astrocytes was validated by RT–qPCR for cell-type-specific markers.

#### Oil Red O staining

Frozen brain sections were stained with freshly prepared Oil Red O working solution for 30 min, briefly differentiated in 60% isopropanol, rinsed with PBS, and counterstained with hematoxylin. Lipid deposition was examined under a light microscope.

#### Filipin staining

Frozen brain sections were fixed in 4% PFA for 30 min, then incubated with Filipin III (0.1 mg/mL) for 1 h at room temperature in the dark. Sections were washed thoroughly with PBS. Free cholesterol distribution was imaged by confocal microscopy (excitation 405 nm) using identical acquisition settings across groups.

#### Nissl staining

Paraffin-embedded brain sections were deparaffinized and rehydrated through a graded alcohol series. Sections were stained with Toluidine Blue for 10 min, followed by differentiation in 0.1% acetic acid for 2 min and rinsing in tap water. Nissl-positive neurons in hippocampal regions, including CA1, CA3, and DG, were imaged using CaseViewer 2.4. The number of Nissl-positive neurons within defined regions of interest was quantified using ImageJ software and expressed as a percentage relative to the control group.

#### Golgi staining

Golgi staining was performed according to the manufacturer’s protocol (FD NeuroTech, PK401). Briefly, mouse brains were removed after isoflurane anesthetization and immersed in a mixture of Solutions A + B for 2 weeks at room temperature in the dark. The brains were then transferred to Solution C for another 7 days at 4 °C, under dark conditions. Afterward, brains were sliced into 100-μm-thick sections using a Vibratome (VT1200S; Leica, Germany). Dendritic spine density of CA1 pyramidal neurons was quantified using ImageJ software. Images were obtained using a microscope (Ni-E, Nikon, Japan), and dendritic complexity of CA1 neurons was assessed through Sholl analysis utilizing the Simple Neurite Tracer plugin in ImageJ. Dendritic spine imaging was performed with a 100× oil immersion lens.

### Cell culture and treatment

#### Isolation and culture of primary microglia and astrocytes

Primary glial cells were isolated from the cerebral cortices of postnatal day 0–3 C57BL/6J mice. After removing meninges and blood vessels, cortical tissues were minced and digested with 0.25% trypsin-EDTA at 37 °C for 15 min. The digestion was neutralized using DMEM (C11885500BT, Gibco) with 10% FBS. The tissue was gently triturated, filtered through a 70 μm strainer, and centrifuged at 1000 × *g* for 5 min. The pellet was resuspended in DMEM containing 10% FBS and 1% penicillin/streptomycin (NCM Biotech, C100C5) and plated into poly-D-lysine-coated T25 flasks.

Cells were maintained at 37 °C in 5% CO₂ with medium changes every 3 days. After 10–14 days, microglia were isolated by shaking flasks at 200 rpm for 1 h at 37 °C. The supernatant was collected, centrifuged, and microglia were resuspended and cultured in DMEM containing 10% FBS for subsequent experiments. The purity of microglia was confirmed by Iba1 immunostaining (>95%). The remaining adherent astrocytes were trypsinized, replated, and expanded, with purity exceeding 95% as assessed by GFAP immunostaining.

#### BV2 microglia cell culture and treatment

The BV2 murine microglial cell line (Procell Life Science & Technology, China) was maintained in DMEM supplemented with 10% FBS and 1% penicillin/streptomycin at 37 °C in a humidified atmosphere with 5% CO₂. For direct stimulation experiments, primary microglia and BV2 cells were treated with 1 μg/mL recombinant LCN2 (rLCN2; R&D Systems) for 24 h prior to collection of lysates or culture supernatants [27]. For cell viability assays, BV2 cells were exposed to increasing concentrations of rLCN2 (0, 500 ng/mL, 1 μg/mL, or 2 μg/mL) for 24 h. For polarization-related experiments, BV2 cells were stimulated with LPS (100 ng/mL; Sigma-Aldrich) or IL-4 (20 ng/mL; R&D Systems) in the presence or absence of rLCN2 (1 μg/mL) for 24 h [28]. For mTOR inhibition experiments, BV2 cells were pretreated with rapamycin (100 nM; APExBIO, A8167) for 24 h, followed by stimulation with rLCN2 (1 μg/mL) for an additional 24 h [29]. Rapamycin was maintained during the subsequent LCN2 stimulation period. Vehicle-treated cells served as controls.

#### C8-D1A astrocyte culture

The C8-D1A mouse astrocyte cell line (Vigen Biotechnology, China) was cultured in DMEM supplemented with 10% FBS and 1% penicillin/streptomycin at 37 °C and 5% CO₂.

#### SCAP overexpression and NF-κB inhibition in primary astrocytes

The SCAP overexpression plasmid (pcDNA3.1-SCAP) and corresponding empty vector control (pcDNA3.1-NC) were constructed by GenePharma. Primary astrocytes were transfected with the SCAP overexpression plasmid using Lipofectamine™ 3000 (Thermo Fisher Scientific, USA) according to the manufacturer’s instructions. Twenty-four hours after transfection, cells were treated with the NF-κB inhibitor ammonium pyrrolidinedithiocarbamate (PDTC; 10 μM; Sigma-Aldrich) or vehicle for an additional 24 h [30]. Cells were then harvested for protein extraction, and culture supernatants were collected for LCN2 measurement.

#### siRNA transfection

Gene knockdown was performed using specific small interfering RNAs (siRNAs) targeting SCAP and LCN2 in primary astrocytes or C8-D1A astrocytes, and 24p3R in BV2 microglia. All siRNAs were purchased from GenePharma (China). Transient transfection was carried out using Lipofectamine™ 3000 according to the manufacturer’s protocol. Cells were seeded at 60–80% confluence and transfected with siRNA–lipid complexes prepared in Opti-MEM™. After 6 h, the transfection medium was replaced with fresh complete medium. Cells were cultured for 48 h post transfection before any further treatments. Knockdown efficiency was confirmed at 72 h after transfection by RT–qPCR and western blot analysis. The sequences of all siRNAs are listed in Supplementary Table 1.

For glucose stimulation experiments, transfected astrocytes were exposed to normal glucose (NG, 5.5 mM) or high glucose (HG, 33 mM) conditions for 24 h prior to sample collection or co-culture. In BV2 microglia, cells transfected with si-24p3R were treated with or without rLCN2 for 24 h before harvesting.

#### Transwell co-culture experiment

A Transwell co-culture system (Corning, USA) was used to model indirect communication between astrocytes and microglia. C8-D1A astrocytes were seeded in the upper inserts and transfected with either non-targeting control siRNA (si-NC), siRNA targeting SCAP (si-SCAP), or siRNA targeting LCN2 (si-LCN2). BV2 microglia were plated in the lower wells and maintained separately. After 24 h, astrocytes were switched to either NG or HG medium for an additional 24 h. The inserts were then gently washed with HBSS to remove residual medium and transferred onto BV2-containing wells for 24 h of indirect co-culture. Unless otherwise specified, BV2 cells in the lower chamber were maintained in standard complete medium during the co-culture period.

For downstream microglial analyses, the following co-culture groups were established: (1) BV2 cells co-cultured with NG-treated si-NC astrocytes (control); (2) BV2 cells co-cultured with HG-treated si-NC astrocytes; (3) BV2 cells co-cultured with HG-treated si-SCAP astrocytes; (4) BV2 cells co-cultured with HG-treated si-LCN2 astrocytes. After co-culture, BV2 cells and lower-chamber supernatants were collected for Western blotting, RT–qPCR, immunofluorescence staining, BODIPY staining, or ELISA analysis, as appropriate.

#### Rescue and receptor-interference experiments in the co-culture system

To evaluate whether exogenous LCN2 could rescue the effects of astrocytic SCAP knockdown, rLCN2 was added to the lower chamber medium during the co-culture period in the HG + si-SCAP condition. BV2 microglia were collected after 24 h for downstream analyses.

To investigate whether microglial 24p3R mediates LCN2-dependent mTOR signaling in the astrocyte–microglia co-culture system, BV2 cells were first transfected with si-NC or si-24p3R for 48 h. The transfected BV2 cells were then co-cultured with HG-treated astrocytes transfected with si-NC or si-SCAP, and exogenous rLCN2 was added to the lower chamber medium where indicated. After 24 h of co-culture, BV2 cells were harvested for Western blot analysis.

### Enzyme-linked immunosorbent assay (ELISA)

LCN2 levels in mouse plasma, hippocampal homogenates, and primary astrocyte culture supernatants were quantified using a mouse LCN2 ELISA kit (KE10045, Proteintech) according to the manufacturer’s instructions. Briefly, hippocampal tissues were homogenized, and the clarified supernatants were collected for ELISA analysis.

#### Cell viability assay

Cell viability was assessed using the Cell Counting Kit-8 (CCK-8; Beyotime Biotechnology, China), following the manufacturer’s protocol. Briefly, cells were seeded in 96-well plates and subjected to the indicated treatments. After treatment, cells were incubated with CCK-8 working solution (1:10 dilution) for 4 h at 37 °C in the dark. Absorbance was measured at 450 nm using a microplate reader (BioTek Instruments, USA). Blank wells containing medium and CCK-8 solution without cells were used for background correction. Cell viability was expressed as a percentage of the control group: [(OD sample–OD blank)/(OD control–OD blank)] × 100%.

#### Immunofluorescence and lipid droplet staining in brain tissue and cultured cells

Immunofluorescence staining was performed on brain tissue sections or cultured cells (BV2 and primary microglia) fixed with 4% PFA. The following primary antibodies were used: LCN2 (1:1000, AF1857, R&D Systems), PLIN2 (1:2000, 15294-1-AP, Proteintech), 24p3R (1:2000, abs119942, Absin), SCAP (1:3000, bs-3862R, Bioss), Iba1 (1:2000, ET1705-78, HUABIO), GFAP (1:3000, 16825-1-AP, Proteintech), NeuN (1:2000, 26975-1-AP, Proteintech), CD68 (1:1000, MCA1957T, Bio-Rad), CD206 (1:1000, 18704-1-AP, Proteintech), iNOS (1:2000, HA722031, HUABIO), IL-1β (1:1000, AF02479, AiFang Biological), and IL-6 (1:1000, WL02841, Wanleibio). For multiplex staining, a TSA-based kit (AFIHC024, AiFang Biological) was used. Samples were blocked with 3% bovine serum albumin (BSA) and 0.3% Triton X-100 for 1 h at room temperature, followed by incubation with primary antibodies overnight at 4 °C. After washing with PBS, appropriate HRP-conjugated secondary antibodies (AFIHC002/AFIHC003, AiFang Biological) were applied for 1 h at room temperature. Lipid droplets (LD) were stained using BODIPY 493/503 (1:1000, D3922, Thermo Fisher) or Lipi Red (1 μmol/L, Dojindo). Nuclei were counterstained with DAPI (Beyotime, Biotechnology). Fluorescence images were captured using a confocal microscope (FV3000, Olympus, Tokyo, Japan).

#### Western blotting

Protein extracts were prepared from hippocampal tissues or cultured cells using ice-cold RIPA lysis buffer containing protease and phosphatase inhibitors (FD0819, Fdbio Science, China). After sonication and centrifugation at 12,000 × *g* for 10 min at 4 °C, the supernatants were collected and quantified. For isolation of cytosolic and nuclear fractions, the Nuclear and Cytoplasmic Protein Extraction Kit (P0027, Beyotime) was used. Equal amounts of protein were separated on 4–20% SDS-PAGE gels (ET15420, ACE Biotechnology) and transferred to PVDF membranes (Millipore, USA). Membranes were blocked with 5% non-fat milk for 2 h at room temperature and incubated overnight at 4 °C with the following primary antibodies: SCAP (1:3000, ab190103, Abcam), LCN2 (1:1000, AF16477, AiFang Biological), NF-κB p65 (1:1000, HA721307, HUABIO), p-p65 (1:1000, WL02169, Wanleibio), PLIN2 (1:3000, A6276, ABclonal), 24p3R (1:1000, abs119942, Absin), iNOS (1:2000, HA722031, HUABIO), CD68 (1:1000, MCA1957T, Bio-Rad), IL-1β (1:1000, WL00891, Wanleibio), IL-6 (1:1000, WL02841, Wanleibio), TNF-α (1:1000, WL01581, Wanleibio), mTOR (1:1000, WL02477, Wanleibio), p-mTOR (1:1000, WL03694, Wanleibio), S6K1 (1:1000, WL03839, Wanleibio), p-S6K1 (1:1000, WL04213, Wanleibio), Lamin B1 (1:1000, WL01775, Wanleibio), GAPDH (1:10,000, 60004-1-Ig, Proteintech) and β-actin (1:10,000, 20536-1-AP, Proteintech). After washing, membranes were incubated with HRP-conjugated goat anti-rabbit or goat anti-mouse secondary antibody (1:5000, Biosharp Biotech) for 1 h at room temperature. Protein bands were visualized using a chemiluminescence system (FD8020, Fdbio Science) and quantified with ImageJ software. Target protein levels were normalized to loading controls and expressed relative to the control group.

#### Quantitative real-time PCR (RT-qPCR)

Total RNA was extracted from tissues or cultured cells using the Total RNA Extraction Kit (AG21024, Accurate Biology, China) according to the manufacturer’s instructions. cDNA synthesis was performed using the High-Capacity cDNA Reverse Transcription Kit (AG11711, Accurate Biology). For quantitative PCR, SYBR Green qPCR Master Mix (AG11740, Accurate Biology) was used, and amplification was performed on an ABI Prism 7000 system (Applied Biosystems, USA). β-actin was used as an internal control for normalization. The relative expression of target genes was calculated using the 2^−ΔΔCt^ method. Samples with Ct values greater than 35 were excluded from the analysis. All primers used are listed in Supplementary Table 2.

#### Flow cytometry analysis

Following treatment, BV2 cells were harvested and resuspended at a concentration of 1 × 10⁶ cells/mL. To block Fc receptors, cells were incubated with anti-CD16/32 antibody (E-AB-F0997A, Elabscience) for 30 min at 4 °C. Cells were then stained with surface markers F4/80 (E-AB-F0995C, Elabscience) and CD86 (E-AB-F0994E, Elabscience) for 30 min at 4 °C in the dark. For intracellular staining, cells were permeabilized using a permeabilization buffer and incubated with anti-CD206 antibody (141705, BioLegend). Data acquisition was performed on a BD Influx flow cytometer (BD Biosciences), and the results were analyzed using FlowJo software (v10.10.0). Specific gating strategies and compensation settings were applied to ensure the accurate analysis of microglial populations.

#### Multiplex cytokine analysis

Cytokine levels in cell culture supernatants were quantified using a Mouse Magnetic Luminex Assay kit (R&D Systems) according to the manufacturer’s instructions. The assay panel included FABP4, TNF-α, CCL2, IL-1β, IL-6, VEGF, ICAM-1, CXCL10, CCL3, IGF-1, angiopoietin-2, and RAGE. IL-1β, IL-6, and TNF-α were selected for quantitative analysis.

#### RNA sequencing and analysis

Total RNA was extracted from hippocampal tissues of HFD fl/fl and HFD cKO mice (*n* = 3 per group) using TRIzol reagent (Thermo Fisher, 15596026) according to the manufacturer’s instructions. RNA quantity and integrity were assessed using a NanoDrop 2000 spectrophotometer (Thermo Scientific) and an Agilent 2100 Bioanalyzer. RNA-seq libraries were prepared using the VAHTS Universal V6 RNA-seq Library Prep Kit with poly(A) mRNA enrichment and sequenced on an Illumina platform by OE Biotech Co., Ltd (Shanghai, China). Clean reads were aligned to the mouse reference genome, and gene expression levels were quantified. Differential expression analysis was performed using DESeq2, with *P* values adjusted for multiple testing using the Benjamini–Hochberg method. Differentially expressed genes (DEGs) were defined by an adjusted *P* value < 0.05 and |log_2_FoldChange| ≥ 1.

#### Untargeted metabolomics analysis

BV2 microglial cells were divided into control and rLCN2-treated groups (*n* = 6 per group). After treatment, cells were washed with ice-cold PBS, harvested, and metabolites were extracted using pre-cooled methanol/acetonitrile/water (2:2:1, v/v). The extracts were vortexed, incubated at −20 °C to precipitate proteins, and centrifuged to collect the supernatants. The remaining pellets were further extracted with acetonitrile/water (1:1, v/v), and the combined supernatants were used for subsequent analysis. Untargeted metabolomic profiling was performed by OE Biotech Co., Ltd (Shanghai, China) using liquid chromatography–mass spectrometry (LC–MS). Raw data were processed using XCMS for peak detection, retention time alignment, and peak area quantification. Multivariate and univariate statistical analyses were conducted to identify metabolic alterations, and differential metabolites were identified based on a variable importance in projection (VIP) > 1 and *P* value < 0.05.

#### Co-Immunoprecipitation (Co-IP)

Primary astrocytes were cultured under normal- or high-glucose conditions for 24 h. Cells were lysed on ice using RIPA buffer supplemented with protease inhibitors (FD1002, Fdbio Science) for 20 min. After centrifugation at 12,000 × *g* for 20 min at 4 °C, supernatants were collected, and an aliquot was reserved as the input control. Equal amounts of protein lysates were incubated with species-matched normal IgG or specific antibodies against SCAP (12266-1-AP, Proteintech) or LCN2 (AF1857, R&D Systems) overnight at 4 °C with gentle rotation. Protein G agarose beads (HY-K0204, MCE) were then added and incubated for an additional 4 h at 4 °C. Beads were washed three times with lysis buffer, and bound proteins were eluted with SDS loading buffer and denatured at 100 °C for 8 min. Eluates were analyzed by Western blotting.

#### CUT&RUN-qPCR

CUT&RUN-qPCR was performed in primary astrocytes using a commercial CUT&RUN kit (Vazyme Biotech, China) according to the manufacturer’s instructions. Briefly, primary astrocytes were harvested and immobilized on concanavalin A (ConA) magnetic beads, followed by overnight incubation at 4 °C with antibodies against NF-κB p65 (HA721307, HUABIO) or with species-matched IgG as a negative control. After washing, pG-MNase was added, and chromatin digestion was initiated by the addition of CaCl₂ for 2 h at 4 °C. The reaction was terminated, and the released DNA fragments were purified and subjected to quantitative PCR analysis. Enrichment of target regions within the LCN2 promoter was normalized to IgG controls. Primer sequences are listed in Supplementary Table 3 (GENEray Biotechnology, China).

#### Statistical analysis

Data normality was assessed using the Shapiro–Wilk test prior to statistical analysis. For comparisons between two groups, an unpaired two-tailed Student’s *t*-test was applied. For comparisons among three or more groups, one-way ANOVA followed by Tukey’s multiple comparisons test, or two-way ANOVA followed by Tukey’s or Sidak’s post hoc test, was used according to the experimental design. Data are expressed as mean ± standard error of the mean (SEM), unless otherwise noted in the figure legends. All experiments included at least three independent biological replicates (*n* ≥ 3), where biological replicates were defined as independent samples, experiments, or animals. Statistical analyses and graph generation were performed using GraphPad Prism software (version 10). *P* value < 0.05 was considered statistically significant.

#### Ethics approval

All experimental procedures were conducted in accordance with the ARRIVE guidelines and institutional regulations for animal care and use, and were approved by the Institutional Animal Care and Use Committee of Zhongda Hospital, Southeast University, China (Approval No. 20210301022).

## Results

### Astrocytic SCAP is selectively upregulated in the hippocampus of HFD-fed mice

**Fig. 1:**
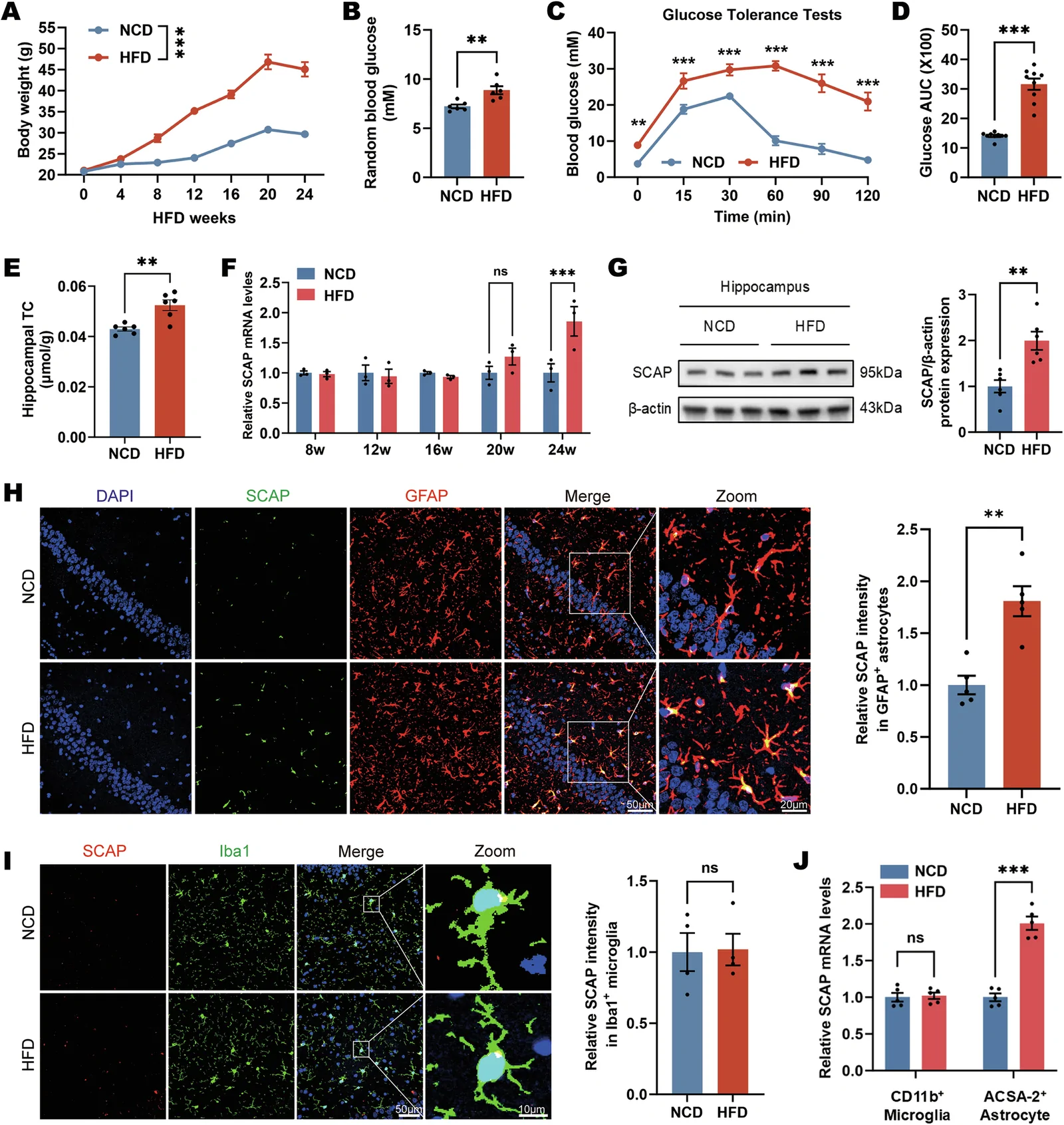
SCAP is selectively upregulated in hippocampal astrocytes of HFD-fed mice. **A** Body weight trajectories of mice fed a normal chow diet (NCD) or high-fat diet (HFD) for 24 weeks (*n* = 6 mice per group). **B** Random blood glucose levels after 24 weeks of dietary intervention (*n* = 6 mice per group). **C** Intraperitoneal glucose tolerance test (IPGTT) curves after 24 weeks of dietary intervention (*n* = 9 mice per group). **D** Quantification of the corresponding area under the curve (AUC) from the IPGTT in **C** (*n* = 9 mice per group). **E** Biochemical quantification of hippocampal total cholesterol (TC) levels in NCD and HFD mice (*n* = 6 mice per group). **F** RT-qPCR analysis of hippocampal SCAP mRNA expression at 8, 12, 16, 20, and 24 weeks of dietary intervention (*n* = 3 mice per group). **G** Representative immunoblotting images and quantification of SCAP protein expression in hippocampal lysates from NCD and HFD mice after 24 weeks of dietary intervention (*n* = 6 mice per group). β-actin was used as the loading control. **H** Representative confocal images of hippocampal sections co-stained for SCAP and GFAP, with DAPI used to label nuclei. Insets show higher-magnification views. Quantification of SCAP fluorescence intensity in GFAP⁺ astrocytes is shown on the right (*n* = 5 mice per group). Scale bars, 50 μm (overview) and 20 μm (enlarged images). **I** Representative confocal images of hippocampal sections co-stained for SCAP and Iba1, with DAPI used to label nuclei. Insets show higher-magnification views. Quantification of SCAP fluorescence intensity in Iba1⁺ microglia is shown on the right (*n* = 4 mice per group). Scale bars, 50 μm (overview) and 10 μm (enlarged images). **J** RT-qPCR analysis of SCAP mRNA expression in MACS-isolated CD11b⁺ microglia and ACSA-2⁺ astrocytes from NCD and HFD mice (*n* = 5 mice per group). Data are presented as mean ± SEM. Statistical analyses were performed using unpaired two-tailed Student’s *t*-test (**B**, **D**–**I**) or two-way ANOVA (**A**, **C**), followed by post hoc tests. ^*^P < 0.05, ^**^P < 0.01, ^***^P < 0.001; ns, not significant.

A T2DM mouse model was established by feeding mice a 60% kcal HFD. Compared with mice fed a NCD, HFD-fed mice exhibited progressive body weight gain (Fig. 1A), elevated random blood glucose levels (Fig. 1B), increased fasting blood glucose (Fig. S1A), impaired glucose tolerance (Fig. 1C, D), and dyslipidemia, including increased TC, TG, and LDL-C levels, whereas HDL-C levels were not significantly altered (Fig. S1B–E). In parallel, lipid homeostasis in the brain was altered, as evidenced by increased neutral lipid accumulation in the hippocampus detected by Oil Red O and LD staining (Fig. S1F, G), along with elevated hippocampal TC and TG levels (Figs. 1E and S1H). Filipin III staining further revealed co-localization of cholesterol with GFAP⁺ astrocytes in the hippocampus (Fig. S1I).

Given the role of SCAP in regulating cellular cholesterol metabolism, hippocampal SCAP expression was assessed during the progression of HFD feeding. Hippocampal SCAP mRNA levels remained comparable at 8–20 weeks but were markedly increased at 24 weeks in HFD-fed mice (Fig. 1F). Consistently, immunoblotting confirmed elevated SCAP protein levels in hippocampal tissue after 24 weeks of HFD feeding (Fig. 1G). Immunofluorescence analysis showed that SCAP was predominantly localized to GFAP⁺ astrocytes, whereas no significant changes were observed in Iba1⁺ microglia (Fig. 1H, I). Cell-type-specific isolation using MACS further confirmed these findings. The purity of isolated cells was validated by enrichment of cell-type–specific markers, including Cx3cr1 for microglia and GFAP for astrocytes (Fig. S1J–L). RT-qPCR analysis demonstrated increased SCAP mRNA expression in astrocytes, but not in microglia, from HFD-fed mice (Fig. 1J). Consistent with in vivo observations, HG stimulation increased SCAP protein levels in primary astrocytes, whereas microglia exhibited only a modest, statistically non-significant increase (Fig. S2).

Behavioral assessments revealed that HFD-fed mice exhibited impaired spatial learning and memory. In the MWM, HFD-fed mice showed prolonged escape latencies during acquisition training, along with reduced path length in the target quadrant and fewer platform crossings during the probe trial (Fig. S3A–C). In addition, spontaneous alternation performance in the Y-maze was significantly reduced (Fig. S3D), and long-term (24 h), but not short-term (1 h), recognition memory was impaired in the NOR test (Fig. S3E, F). In contrast, OFT revealed no significant differences in locomotor activity or anxiety-related behavior between groups (Fig. S3G–I). Together, these results demonstrate that chronic HFD feeding induces systemic metabolic dysfunction, hippocampal lipid accumulation, astrocyte-specific upregulation of SCAP, and cognitive impairment.

### Astrocyte-specific SCAP deletion improves cognitive performance and attenuates microglial activation in HFD-fed mice

**Fig. 2:**
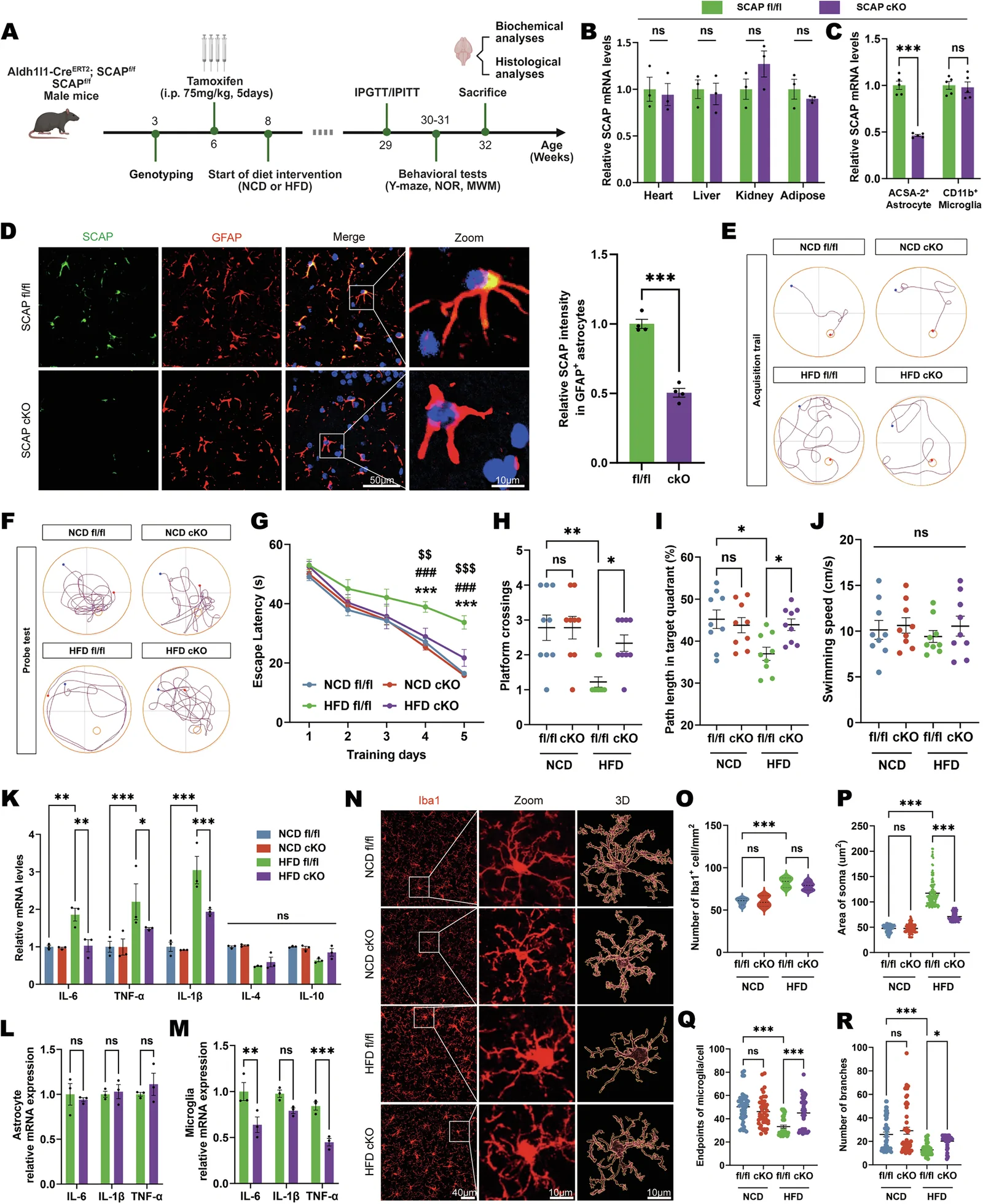
Astrocyte-specific SCAP knockout improves cognitive performance and attenuates microglial activation in HFD-fed mice. **A** Schematic timeline of tamoxifen induction, diet intervention, metabolic testing, behavioral assessments, and tissue collection in male Aldh1l1-CreERT2; SCAP fl/fl (SCAP cKO) mice and SCAP fl/fl littermate controls. Created in BioRender (https://BioRender.com/rra5d9o). **B** RT-qPCR analysis of SCAP mRNA levels in peripheral tissues, including heart, liver, kidney, and adipose tissue, from SCAP fl/fl and SCAP cKO mice (*n* = 3 mice per group). **C** RT-qPCR analysis of SCAP mRNA levels in MACS-isolated hippocampal ACSA-2^+^ astrocytes and CD11b^+^ microglia from SCAP fl/fl and SCAP cKO mice (*n* = 5 mice per group). **D** Representative confocal images of hippocampal sections co-stained for SCAP and GFAP, with DAPI counterstaining. Insets show higher-magnification views. Quantification of SCAP fluorescence intensity in GFAP^+^ astrocytes is shown on the right (*n* = 4 mice per group). Scale bars, 50 μm (overview) and 10 μm (enlarged images). Representative swim trajectories in the Morris water maze (MWM) during the acquisition phase (**E**) and probe trial (**F**). **G** Escape latency during five days of MWM acquisition training (*n* = 9 mice per group). Probe trial analyses showing the number of platform crossings (**H**), path length in the target quadrant (**I**), and swimming speed (**J**) (*n* = 9 mice per group). **K** RT-qPCR analysis of inflammatory cytokine mRNA levels in whole hippocampal tissue (*n* = 3 mice per group). MACS-based cell type-specific RT-qPCR analysis of cytokine mRNA expression in astrocytes (**L**) and microglia (**M**) (*n* = 3 mice per group). **N** Representative confocal images of Iba1⁺ microglia, higher-magnification views, and corresponding 3D reconstructions of microglia from each group. Scale bars, 40 μm (overview) and 10 μm (enlarged images and 3D reconstructions). Quantification of microglial morphological parameters, including the number of Iba1^+^ cells per mm^2^ (**O**), soma area (**P**), endpoints per microglia (**Q**), and branch number (**R**) (*n* = 4–5 mice per group; a total of 60–155 microglia analyzed across groups). Experimental groups are defined as: NCD fl/fl, SCAP fl/fl mice fed a normal chow diet; NCD cKO, astrocyte-specific SCAP cKO mice fed a normal chow diet; HFD fl/fl, SCAP fl/fl mice fed a high-fat diet; and HFD cKO, astrocyte-specific SCAP cKO mice fed a high-fat diet. Data are presented as mean ± SEM. Statistical analyses were performed using unpaired two-tailed Student’s *t*-test (**B**–**D**, **L**, **M**), one-way ANOVA (**H**–**K**, **O**–**R**), or two-way ANOVA (**G**), followed by post hoc tests. In **G**, * indicates NCD fl/fl vs HFD fl/fl, # indicates NCD cKO vs HFD fl/fl, and $ indicates HFD fl/fl vs HFD cKO at the corresponding training day. For the remaining panels, statistical comparisons are indicated by brackets. ^*^P < 0.05, ^**^P < 0.01, ^***^P < 0.001; ns, not significant.

To investigate the role of astrocytic SCAP in DACI, an astrocyte-specific SCAP cKO mouse model was generated by crossing Aldh1l1-CreERT2 mice with SCAP fl/fl mice. Tamoxifen administration (75 mg/kg/day for 5 consecutive days at 6 weeks of age) induced CreERT2-mediated excision of the floxed SCAP allele in Aldh1l1-expressing astrocytes (Fig. 2A). Genotyping confirmed Cre-dependent recombination of the floxed SCAP allele in Aldh1l1-CreERT2; SCAP fl/fl mice (Fig. S4A). RT-qPCR analysis showed that SCAP mRNA levels in peripheral tissues, including heart, liver, kidney, and adipose tissue, were comparable between SCAP fl/fl and cKO mice, whereas SCAP expression was markedly reduced in MACS-purified astrocytes but unchanged in microglia (Fig. 2B, C). Consistently, immunofluorescence analysis revealed reduced SCAP signal in GFAP⁺ astrocytes (Fig. 2D), with no detectable changes in Iba1⁺ microglia or NeuN⁺ neurons (Fig. S5A, B), confirming cell-type-specific deletion.

Cognitive performance was assessed using a battery of behavioral tests. In the MWM, HFD fl/fl mice exhibited prolonged escape latencies during acquisition training (Fig. 2G). Representative swim trajectories from the acquisition phase and probe trial are shown in Fig. 2E, F. In contrast, HFD cKO mice displayed shorter escape latencies and a higher number of platform crossings, along with increased path length within the target quadrant during the probe trial (Fig. 2H, I). Swimming speed did not differ among groups (Fig. 2J). In contrast to the changes observed in spatial learning and memory, spontaneous alternation in the Y-maze and recognition memory in the NOR test were not significantly altered by astrocytic SCAP deletion (Fig. S4B–D).

Neuroinflammatory changes and microglial morphological features were then examined in the hippocampus. RT-qPCR analysis showed increased expression of IL-1β, IL-6, and TNF-α in HFD fl/fl mice, which was attenuated in HFD cKO mice, whereas IL-4 and IL-10 expression remained unchanged (Fig. 2K). MACS-based cell type-specific RT-qPCR analyses further showed reduced IL-6 and TNF-α expression in microglia isolated from HFD cKO mice, while no significant changes were observed in astrocytes (Fig. 2L, M). Morphological analysis of Iba1⁺ microglia revealed that HFD feeding significantly increased microglial density; however, microglial density did not differ between SCAP fl/fl and cKO mice under either NCD or HFD conditions (Fig. 2O). Compared with NCD fl/fl mice, microglia from HFD fl/fl mice exhibited larger soma area and reduced branching complexity. Under HFD conditions, microglia from SCAP cKO mice displayed smaller soma area and increased numbers of endpoints and branches (Fig. 2N–R). In addition, astrocytic SCAP deletion did not significantly affect body weight, glucose tolerance, or systemic and hippocampal lipid profiles under HFD conditions (Fig. S4E–L). Collectively, these results demonstrate that astrocyte SCAP deletion improves hippocampal-dependent cognitive performance and attenuates microglial activation and neuroinflammatory responses in HFD-fed mice, without affecting systemic metabolic parameters.

### Astrocytic SCAP deletion attenuates microglial inflammatory polarization and lipid droplet accumulation in the hippocampus

**Fig. 3:**
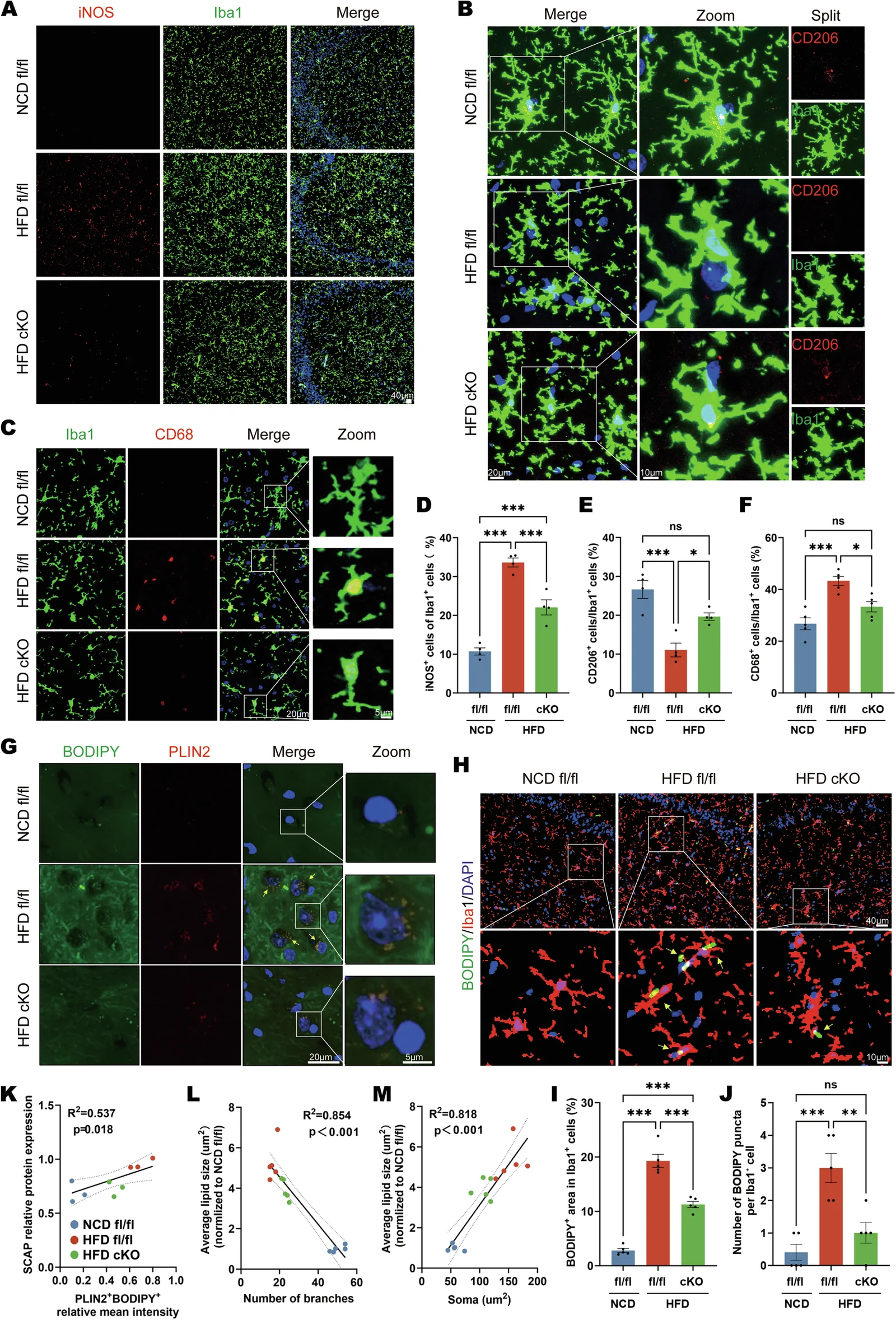
Astrocytic SCAP deletion attenuates microglial inflammatory polarization and lipid droplet accumulation in the hippocampus of HFD-fed mice. **A** Representative confocal images of iNOS⁺ (red) and Iba1⁺ (green) staining in hippocampal sections from NCD fl/fl, HFD fl/fl, and HFD cKO mice. DAPI (blue) was used to label nuclei. Scale bar, 40 μm (*n* = 4 mice per group). **B** Representative confocal images of CD206⁺ (red) and Iba1⁺ (green) staining in hippocampal sections from NCD fl/fl, HFD fl/fl, and HFD cKO mice. DAPI (blue) was used to label nuclei. Insets show higher-magnification views of the boxed regions, with corresponding split channels shown on the right (*n* = 4 mice per group). Scale bars, 20 μm (overview) and 10 μm (enlarged images). **C** Representative confocal images of CD68⁺ (red) and Iba1⁺ (green) staining in hippocampal sections from NCD fl/fl, HFD fl/fl, and HFD cKO mice. DAPI (blue) was used to label nuclei. Insets show higher-magnification views of the boxed regions (*n* = 5 mice per group). Scale bars, 20 μm (overview) and 5 μm (enlarged images). Quantification of the percentage of **D** iNOS⁺ cells, **E** CD206⁺ cells, and **F** CD68⁺ cells among Iba1⁺ microglia in hippocampal sections from NCD fl/fl, HFD fl/fl, and HFD cKO mice (*n* = 4 mice per group for **D** and **E**; *n* = 5 mice per group for **F**). **G** Representative confocal images of BODIPY (green) and PLIN2 (red) staining in hippocampal sections from NCD fl/fl, HFD fl/fl, and HFD cKO mice. DAPI (blue) was used to label nuclei. Insets show higher-magnification views of the boxed regions (*n* = 5 mice per group). Scale bars, 20 μm (overview) and 5 μm (enlarged images). **H** Representative confocal images of BODIPY (green) and Iba1 (red) co-staining in hippocampal sections from NCD fl/fl, HFD fl/fl, and HFD cKO mice. DAPI (blue) was used to label nuclei. Insets show higher-magnification views of the boxed regions (*n* = 5 mice per group). Scale bars, 40 μm (overview) and 10 μm (enlarged images). Quantification of **I** BODIPY⁺ area within Iba1⁺ cells (%) and **J** the number of BODIPY puncta per Iba1⁺ cell in hippocampal sections from NCD fl/fl, HFD fl/fl, and HFD cKO mice (*n* = 5 mice per group). **K** Pearson correlation analysis between hippocampal SCAP protein levels and PLIN2⁺/BODIPY⁺ relative mean intensity across NCD fl/fl, HFD fl/fl, and HFD cKO mice. Each dot represents one mouse (*n* = 3 mice per group). Pearson correlation analyses between average lipid droplet size and microglial morphological parameters, including **L** branch number and **M** soma area. Each dot represents one mouse, with values averaged from 6–12 microglia per mouse (*n* = 5 mice per group). Data are presented as mean ± SEM. Statistical analyses were performed using one-way ANOVA followed by post hoc tests (**D**–**F**, **I**, **J**) or Pearson correlation analysis (**K**–**M**). ^*^P < 0.05, ^**^P < 0.01, ^***^P < 0.001; ns, not significant.

The effects of astrocytic SCAP deletion on microglial inflammatory and metabolic phenotypes were examined in diabetic mice. Immunofluorescence staining for inducible nitric oxide synthase (iNOS) and CD206 showed that, compared with NCD controls, HFD fl/fl mice exhibited an increased proportion of iNOS⁺ microglia, accompanied by a decreased proportion of CD206⁺ microglia. In contrast, HFD cKO mice displayed a reduced proportion of iNOS⁺ microglia and an increased proportion of CD206⁺ microglia (Fig. 3A, B, D, E). Consistently, immunofluorescence analysis of CD68, a lysosomal marker associated with microglial phagocytic activity, revealed increased CD68 expression in HFD fl/fl mice compared with NCD controls, which was attenuated in HFD cKO mice (Fig. 3C, F).

LD accumulation in the hippocampus was examined by immunofluorescence staining using PLIN2 together with the neutral lipid dye BODIPY. HFD fl/fl mice exhibited a marked increase in PLIN2⁺/BODIPY⁺ co-labeled LD puncta in the hippocampus, which was reduced in HFD cKO mice (Fig. 3G). Co-immunostaining of PLIN2/BODIPY with Iba1 revealed the presence of LDs in microglia. Quantitative analyses further demonstrated that HFD feeding significantly increased LD accumulation in microglia, as indicated by increased BODIPY-positive area and puncta number per cell, whereas these effects were markedly attenuated in HFD cKO mice (Fig. 3H–J). In contrast, co-staining of GFAP with BODIPY or PLIN2 showed minimal LD accumulation in astrocytes, with no significant differences in LD area among NCD fl/fl, HFD fl/fl, and HFD cKO mice (Fig. S4M). Consistently, mRNA expression levels of lipid transport– and efflux–related genes (ABCA1, APOE, and ABCG1) in MACS-isolated astrocytes were comparable across groups (Fig. S4N). Pearson correlation analyses further showed that hippocampal PLIN2⁺/BODIPY⁺ intensity was positively correlated with SCAP protein levels (Fig. 3K). In addition, average LD size was positively correlated with microglial soma area and negatively correlated with branch number (Fig. 3L, M), supporting an association between LD burden and microglial morphological features. Collectively, these results indicate that astrocytic SCAP deletion attenuates microglial pro-inflammatory polarization and LD accumulation in the hippocampus, without detectable changes in astrocytic lipid storage or lipid transport–related gene expression.

### Astrocytic SCAP deletion reduces hippocampal LCN2 expression in HFD-fed mice

**Fig. 4:**
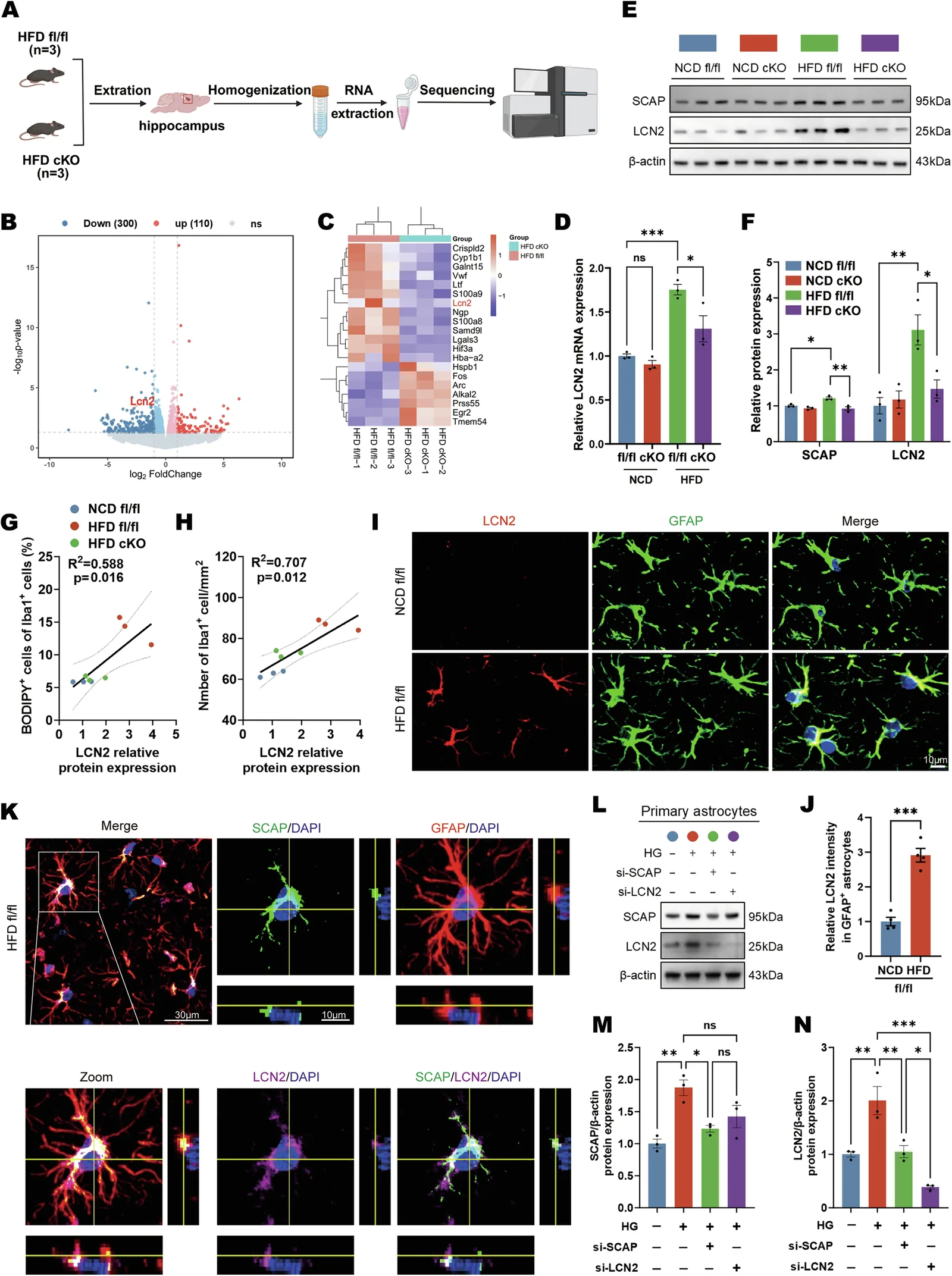
Astrocytic SCAP deletion reduces hippocampal LCN2 expression in HFD-diabetic mice. **A** Schematic overview of the hippocampal RNA-seq workflow in HFD fl/fl and HFD cKO mice, including hippocampal dissection, tissue homogenization, RNA extraction, and sequencing. Created in BioRender (https://BioRender.com/rxyg4u5). **B** Volcano plot of differentially expressed genes identified by RNA-seq analysis in HFD cKO versus HFD fl/fl hippocampal tissues (*n* = 3 mice per group). **C** Heatmap of selected inflammation-related genes identified by RNA-seq analysis, including Lcn2, in HFD fl/fl and HFD cKO hippocampal tissues (*n* = 3 mice per group). **D** RT-qPCR analysis of hippocampal LCN2 mRNA expression in NCD fl/fl, NCD cKO, HFD fl/fl, and HFD cKO mice (*n* = 3 mice per group). **E** Representative Western blot images of SCAP and LCN2 protein expression in hippocampal lysates from NCD fl/fl, NCD cKO, HFD fl/fl, and HFD cKO mice. β-actin was used as the loading control. **F** Quantification of SCAP and LCN2 protein expression in hippocampal lysates from NCD fl/fl, NCD cKO, HFD fl/fl, and HFD cKO mice (*n* = 3 mice per group). Pearson correlation analyses between hippocampal LCN2 protein expression and (**G**) the percentage of BODIPY⁺ cells among Iba1⁺ microglia and (**H**) Iba1⁺ microglial density (cells per mm²) across NCD fl/fl, HFD fl/fl, and HFD cKO mice. Each dot represents one mouse (*n* = 3 mice per group). **I** Representative confocal images of LCN2 (red) and GFAP (green) co-staining in hippocampal sections from NCD fl/fl and HFD fl/fl mice. DAPI (blue) was used to label nuclei. Scale bar, 10 μm. **J** Quantification of relative LCN2 fluorescence intensity in GFAP⁺ astrocytes from NCD fl/fl and HFD fl/fl mice (*n* = 4 mice per group). **K** Representative confocal images of hippocampal sections from HFD fl/fl mice co-stained for SCAP (green), GFAP (red), and LCN2 (magenta), with DAPI (blue) labeling nuclei. Enlarged views of the boxed regions and orthogonal views are shown on the right and below. Scale bars, 30 μm (overview) and 10 μm (enlarged images). **L** Representative Western blot images of SCAP and LCN2 protein expression in primary astrocytes cultured under high-glucose conditions with siRNA-mediated knockdown of SCAP or LCN2. β-actin was used as the loading control. Quantification of **M** SCAP and **N** LCN2 protein expression in primary astrocytes under high-glucose conditions with siRNA-mediated knockdown of SCAP or LCN2 (*n* = 3 independent biological replicates per group). Data are presented as mean ± SEM. Statistical analyses were performed using unpaired two-tailed Student’s *t*-test (**J**), one-way ANOVA followed by post hoc tests (**D**, **F**, **M**, **N**), or Pearson correlation analysis (**G**, **H**). ^*^P < 0.05, ^**^P < 0.01, ^***^P < 0.001; ns, not significant.

To investigate the molecular mechanisms by which astrocytic SCAP regulates microglial activation and lipid metabolic alterations, we performed RNA sequencing on hippocampal tissues from HFD fl/fl and HFD cKO mice (Fig. 4A). Differential expression analysis identified 410 differentially expressed genes (DEGs), including 300 downregulated and 110 upregulated genes (Fig. 4B). Gene Ontology enrichment analysis revealed that downregulated genes were primarily associated with inflammatory and immune-related processes (Fig. S6A). Among these, LCN2 emerged as one of the most significantly downregulated inflammation-related transcripts in HFD cKO mice (Fig. 4C) and was therefore selected for further investigation due to its established role as an astrocyte-derived mediator of microglia-associated neuroinflammatory responses [31].

RT–qPCR and Western blot analyses confirmed that hippocampal LCN2 expression was markedly increased in HFD fl/fl mice but substantially attenuated in HFD cKO mice (Fig. 4D–F). In contrast, plasma LCN2 levels were not significantly altered by astrocytic SCAP deletion (Fig. S6B), suggesting that SCAP-dependent regulation of LCN2 may be primarily restricted to the central nervous system. Hippocampal LCN2 protein abundance positively correlated with both the proportion of BODIPY⁺ microglia and Iba1⁺ microglial density (Fig. 4G, H), linking LCN2 expression to microglial activation and LD accumulation. Immunofluorescence analysis further revealed that HFD-induced LCN2 upregulation was predominantly localized to GFAP⁺ astrocytes, with minimal changes observed in NeuN⁺ neurons or Iba1⁺ microglia (Figs. 4I, J and S6C, D). Consistently, LCN2 expression within astrocytes was markedly reduced in HFD cKO mice (Fig. S6E), and co-localization analysis confirmed that SCAP and LCN2 are enriched within GFAP⁺ astrocytes under HFD conditions (Fig. 4K), supporting a cell- type-specific regulatory relationship.

The regulatory relationship between SCAP and LCN2 was further examined in primary astrocytes. Under HG conditions, SCAP knockdown reduced intracellular LCN2 protein levels, whereas LCN2 silencing did not alter SCAP expression (Fig. 4L–N), indicating that SCAP functions upstream of LCN2. In addition, HG stimulation increased LCN2 secretion into the culture medium, which was attenuated by SCAP knockdown (Fig. S6F). Co-IP analysis showed that SCAP and LCN2 could be detected within the same immunoprecipitated complexes (Fig. S6G), suggesting a potential functional association rather than a direct physical interaction. Collectively, these results identify astrocyte-derived LCN2 as an important downstream effector of SCAP and suggest that SCAP regulates microglial activation and LD accumulation, at least in part, through LCN2 signaling.

### Astrocytic SCAP promotes NF-κB–dependent transcriptional activation of LCN2 under diabetic conditions

**Fig. 5:**
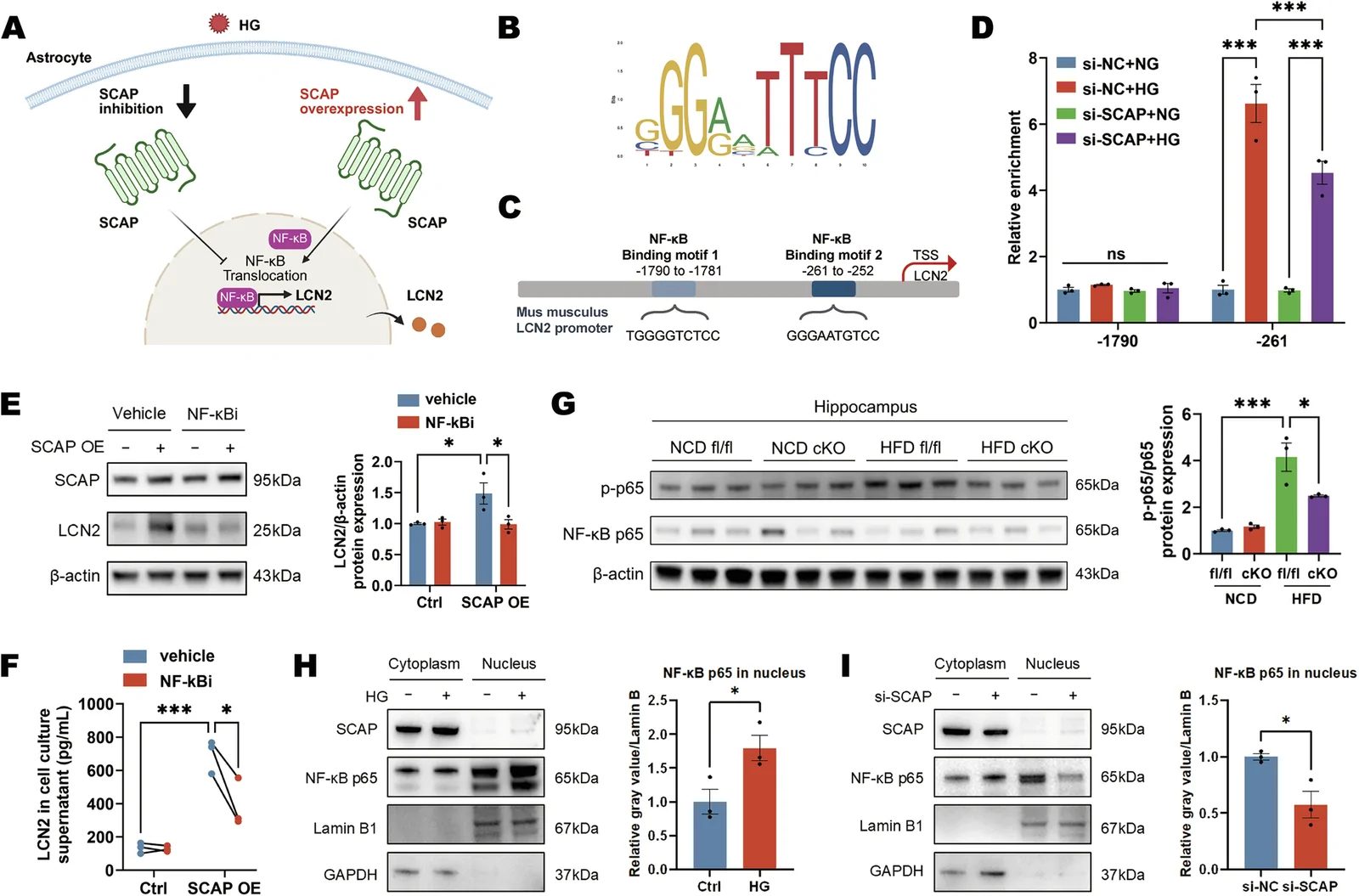
SCAP regulates astrocytic LCN2 expression via NF-κB signaling. **A** Schematic illustration of the proposed mechanism by which SCAP regulates NF-κB-dependent LCN2 expression in astrocytes under HG stimulation. Created in BioRender (https://BioRender.com/ymm1tli). **B** Sequence logo of the predicted NF-κB (p65) binding motif identified within the mouse LCN2 promoter by in silico motif analysis using the JASPAR database. **C** Schematic representation of the mouse LCN2 promoter indicating two predicted NF-κB binding sites located at −1790 to −1781 and −261 to −252 relative to the transcription start site (TSS). **D** CUT&RUN-qPCR analysis of p65 enrichment at the −1790 and −261 regions of the LCN2 promoter in primary astrocytes transfected with si-NC or si-SCAP and cultured under normal-glucose (NG) or high-glucose (HG) conditions (*n* = 3 independent biological replicates per group). **E** Representative Western blot images of SCAP and LCN2 protein expression in primary astrocytes with or without SCAP overexpression in the presence or absence of the NF-κB inhibitor PDTC (10 μM). Quantification of LCN2 protein expression is shown on the right (*n* = 3 independent biological replicates per group). β-actin was used as the loading control. **F** ELISA analysis of LCN2 levels in primary astrocyte-conditioned medium with or without SCAP overexpression in the presence or absence of the NF-κB inhibitor PDTC (10 μM) (*n* = 3 independent biological replicates per group). **G** Representative Western blot images of phosphorylated NF-κB p65 (p-p65) and total p65 in hippocampal lysates from NCD fl/fl, NCD cKO, HFD fl/fl, and HFD cKO mice. Quantification of the p-p65/p65 ratio is shown on the right (*n* = 3 mice per group). β-actin was used as the loading control. **H** Subcellular fractionation followed by western blot analysis of cytoplasmic and nuclear fractions from primary astrocytes cultured under control or high-glucose (HG) conditions. Quantification of nuclear NF-κB p65 is shown on the right (*n* = 3 independent biological replicates per group). Lamin B1 and GAPDH were used as nuclear and cytoplasmic markers, respectively. **I** Subcellular fractionation followed by western blot analysis of cytoplasmic and nuclear fractions from primary astrocytes transfected with control siRNA or si-SCAP. Quantification of nuclear NF-κB p65 is shown on the right (*n* = 3 independent biological replicates per group). Lamin B1 and GAPDH were used as nuclear and cytoplasmic markers, respectively. Data are presented as mean ± SEM. Statistical analyses were performed using one-way ANOVA (**D**, **G**), two-way ANOVA (**E**, **F**), or unpaired two-tailed Student’s *t*-test (**H**, **I**), followed by appropriate post hoc tests where applicable. ^*^P < 0.05, ^**^P < 0.01, ^***^P < 0.001; ns, not significant.

LCN2 is a known NF-κB–responsive gene [32, 33], and SCAP deficiency has been reported to attenuate NF-κB activation in myeloid cells [25]. We therefore examined whether astrocytic SCAP regulates LCN2 expression through NF-κB–dependent transcriptional mechanisms in primary astrocytes exposed to HG stimulation (Fig. 5A). Bioinformatic analysis using the JASPAR database identified putative NF-κB (p65) binding motifs within the promoter region of the mouse LCN2 gene (Fig. 5B, C). CUT&RUN–qPCR analysis further demonstrated that HG stimulation increased p65 occupancy at the endogenous LCN2 promoter, whereas this enrichment was markedly reduced in SCAP-silenced astrocytes (Fig. 5D). In contrast, SCAP overexpression enhanced p65 binding to the LCN2 promoter under HG conditions (Fig. S7). Consistent with these findings, SCAP overexpression increased LCN2 protein expression and secretion in primary astrocytes, and this induction was attenuated by pharmacological inhibition of NF-κB signaling with PDTC (Fig. 5E, F). In vivo, hippocampal tissues from HFD cKO mice exhibited a reduced p-p65/p65 ratio compared with HFD fl/fl controls (Fig. 5G), indicating attenuated hippocampal NF-κB activation following astrocyte-specific SCAP deletion. Subcellular fractionation in primary astrocytes showed that HG stimulation promoted nuclear accumulation of NF-κB p65, whereas SCAP knockdown reduced nuclear p65 levels; SCAP was predominantly localized in the cytoplasmic fraction under these conditions (Fig. 5H, I). Collectively, these findings demonstrate that astrocytic SCAP promotes NF-κB–dependent transcriptional activation of LCN2 under diabetic conditions.

### LCN2 promotes microglial inflammatory activation and lipid droplet accumulation in vitro

**Fig. 6:**
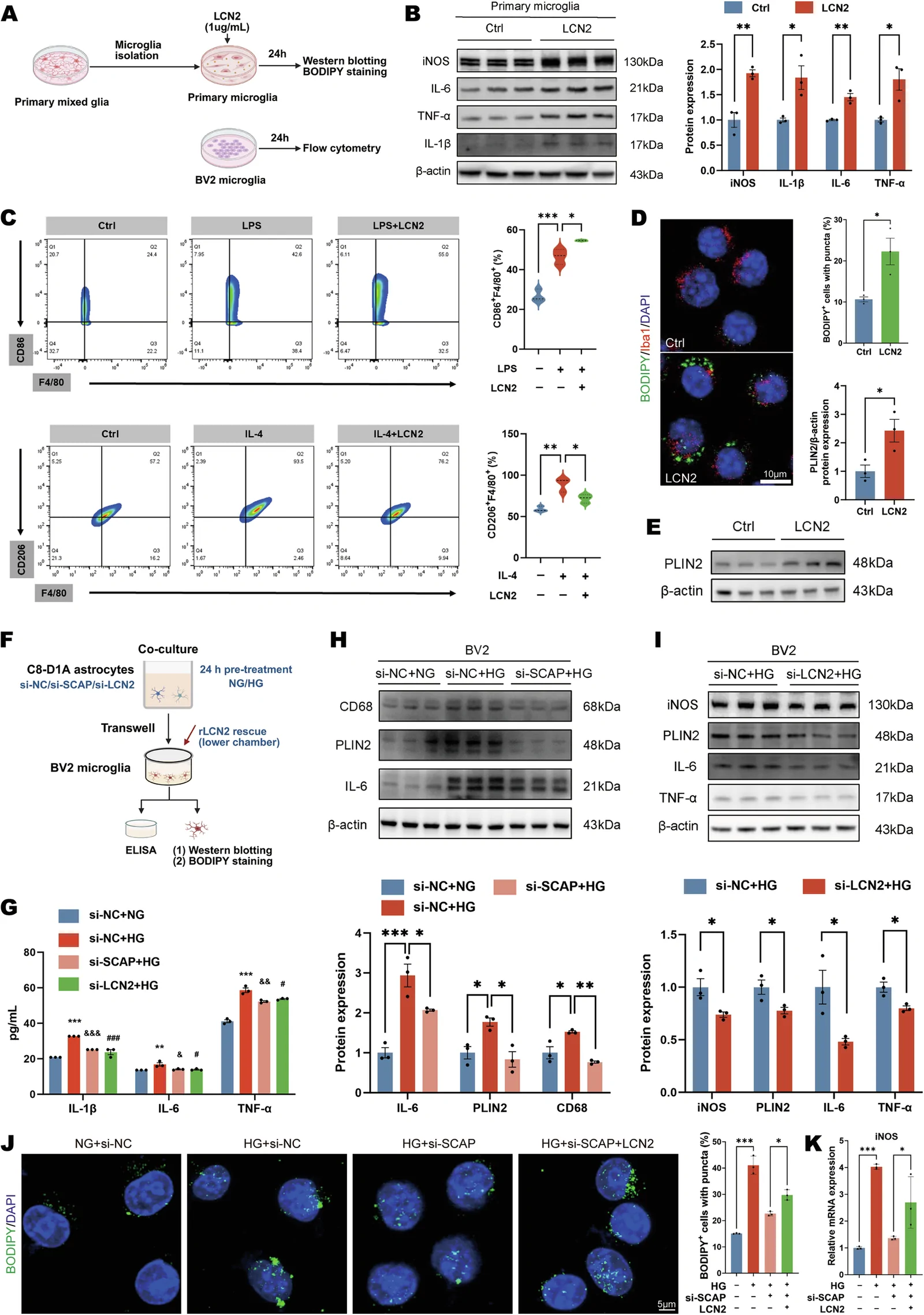
LCN2 induces a pro-inflammatory microglial phenotype and lipid droplet accumulation in vitro. **A** Schematic overview of the experimental design. Primary microglia were treated with recombinant LCN2 (rLCN2, 1 μg/mL, 24 h) for analyses of inflammatory marker expression and lipid droplet (LD) accumulation. BV2 microglia were used for flow cytometry analysis of polarization markers. Created in BioRender (https://BioRender.com/m66mz09). **B** Representative Western blot images of iNOS, IL-1β, IL-6, and TNF-α protein expression in primary microglia following rLCN2 treatment, with quantification shown on the right (*n* = 3 independent biological replicates per group). **C** Flow cytometric analysis of CD86⁺ and CD206⁺ BV2 microglial populations under LPS or IL-4 stimulation, with or without rLCN2 treatment. Representative contour plots and corresponding quantification of CD86⁺ and CD206⁺ cell proportions are shown (*n* = 3 independent biological replicates per group). **D** Representative confocal images of primary microglia stained for Iba1 (red) and BODIPY (green), with DAPI (blue) labeling nuclei, following vehicle (Ctrl) or rLCN2 treatment. Quantification of the percentage of BODIPY⁺ cells among Iba1⁺ microglia and PLIN2 expression is shown on the right (*n* = 3 independent biological replicates per group). Scale bar, 10 μm. **E** Representative Western blot images of PLIN2 protein expression in primary microglia following rLCN2 treatment, with quantification shown on the right (*n* = 3 independent biological replicates per group). **F** Schematic illustration of the Transwell co-culture system. C8-D1A astrocytes transfected with si-NC, si-SCAP, or si-LCN2 were pretreated under normal glucose (NG) or high glucose (HG) conditions for 24 h and then co-cultured with BV2 microglia. For the rescue experiment, recombinant LCN2 (rLCN2) was added to the lower chamber medium during co-culture under the HG + si-SCAP condition. BV2 microglia and culture supernatants were subsequently collected for cytokine, protein, and lipid droplet (LD) analyses. Created in BioRender (https://BioRender.com/x7e40lk). **G** ELISA measurements of IL-1β, IL-6, and TNF-α levels in lower-chamber culture supernatants following co-culture under the indicated conditions (*n* = 3 independent biological replicates per group). **H** Representative Western blot images of CD68, PLIN2, and IL-6 expression in BV2 microglia following co-culture with HG-treated astrocytes transfected with si-NC or si-SCAP, with quantification shown on the right (*n* = 3 independent biological replicates per group). **I** Representative Western blot images of iNOS, PLIN2, IL-6, and TNF-α expression in BV2 microglia following co-culture with HG-treated astrocytes transfected with si-NC or si-LCN2, with quantification shown on the right (*n* = 3 independent biological replicates per group). **J** Representative confocal images of BODIPY staining in BV2 microglia under HG conditions following co-culture with si-SCAP-transfected astrocytes, with or without rLCN2 supplementation. DAPI (blue) labels nuclei. Quantification of BODIPY⁺ lipid droplet puncta is shown on the right (*n* = 3 independent biological replicates per group). Scale bar, 5 μm. **K** RT-qPCR analysis of iNOS mRNA expression in BV2 microglia under HG conditions following co-culture with si-SCAP-transfected astrocytes, with or without rLCN2 supplementation (*n* = 3 independent biological replicates per group). Data are presented as mean ± SEM. Statistical analyses were performed using unpaired two-tailed Student’s *t*-test (**B**, **D**, **E**, **I**) or one-way ANOVA followed by appropriate post hoc tests (**C**, **G**, **H**, **J**, **K**). ^*^P < 0.05, ^**^P < 0.01, ^***^P < 0.001; ns, not significant.

To determine the effects of LCN2 on microglial inflammatory activation and lipid metabolism, we treated primary microglia and BV2 cells with rLCN2 (Fig. 6A). Cell viability assays showed that rLCN2 at 0.5–1 μg/mL did not affect microglia viability, whereas 2 μg/mL reduced viability (Fig. S8A). Therefore, a concentration of 1 μg/mL was used for subsequent experiments. After 24 h of rLCN2 treatment, primary microglia exhibited increased protein expression of pro-inflammatory markers, including iNOS, IL-6, TNF-α, and IL-1β (Fig. 6B). In BV2 cells, rLCN2 further increased the proportion of CD86⁺ cells under LPS stimulation, and reduced the proportion of CD206⁺ cells under IL-4 stimulation (Fig. 6C), indicating a shift toward a pro-inflammatory phenotype. rLCN2 also promoted LD accumulation, as evidenced by an increased proportion of BODIPY⁺ cells with punctate LDs and elevated PLIN2 protein expression in primary microglia (Fig. 6D, E).

A Transwell co-culture system was then established to evaluate the contribution of astrocyte-derived signals (Fig. 6F). Immunofluorescence analysis revealed increased spatial proximity between astrocytes and microglia in HFD-fed mice, suggesting enhanced astrocyte–microglia interactions (Fig. S8B). BV2 microglia exposed to HG–treated C8-D1A astrocytes exhibited increased secretion of IL-1β, IL-6, and TNF-α, which was attenuated by SCAP or LCN2 knockdown in astrocytes (Fig. 6G). Co-culture with HG–stimulated astrocytes increased the expression of CD68, PLIN2, and IL-6 in BV2 microglia, whereas astrocytic SCAP knockdown blunted these responses (Fig. 6H). Similarly, astrocytic LCN2 knockdown reduced the expression of iNOS, PLIN2, IL-6, and TNF-α in co-cultured BV2 microglia (Fig. 6I). In addition, LD accumulation was similarly reduced following SCAP or LCN2 knockdown (Fig. S8C), and immunofluorescence analyses confirmed decreased inflammatory marker expression in microglia (Fig. S8D–G). To further assess whether LCN2 is involved in the effects of astrocytic SCAP on microglial responses, rLCN2 was supplemented in the co-culture system following siRNA-mediated SCAP knockdown in astrocytes. rLCN2 partially restored LD accumulation and increased iNOS mRNA expression in BV2 microglia exposed to SCAP-silenced astrocytes (Fig. 6J, K). These rescue effects suggest that LCN2 at least partly accounts for the SCAP-dependent astrocyte–microglia crosstalk that promotes microglial inflammatory activation and LD accumulation in this HG-induced co-culture model.

### LCN2 activates microglial mTOR signaling via the receptor 24p3R

**Fig. 7:**
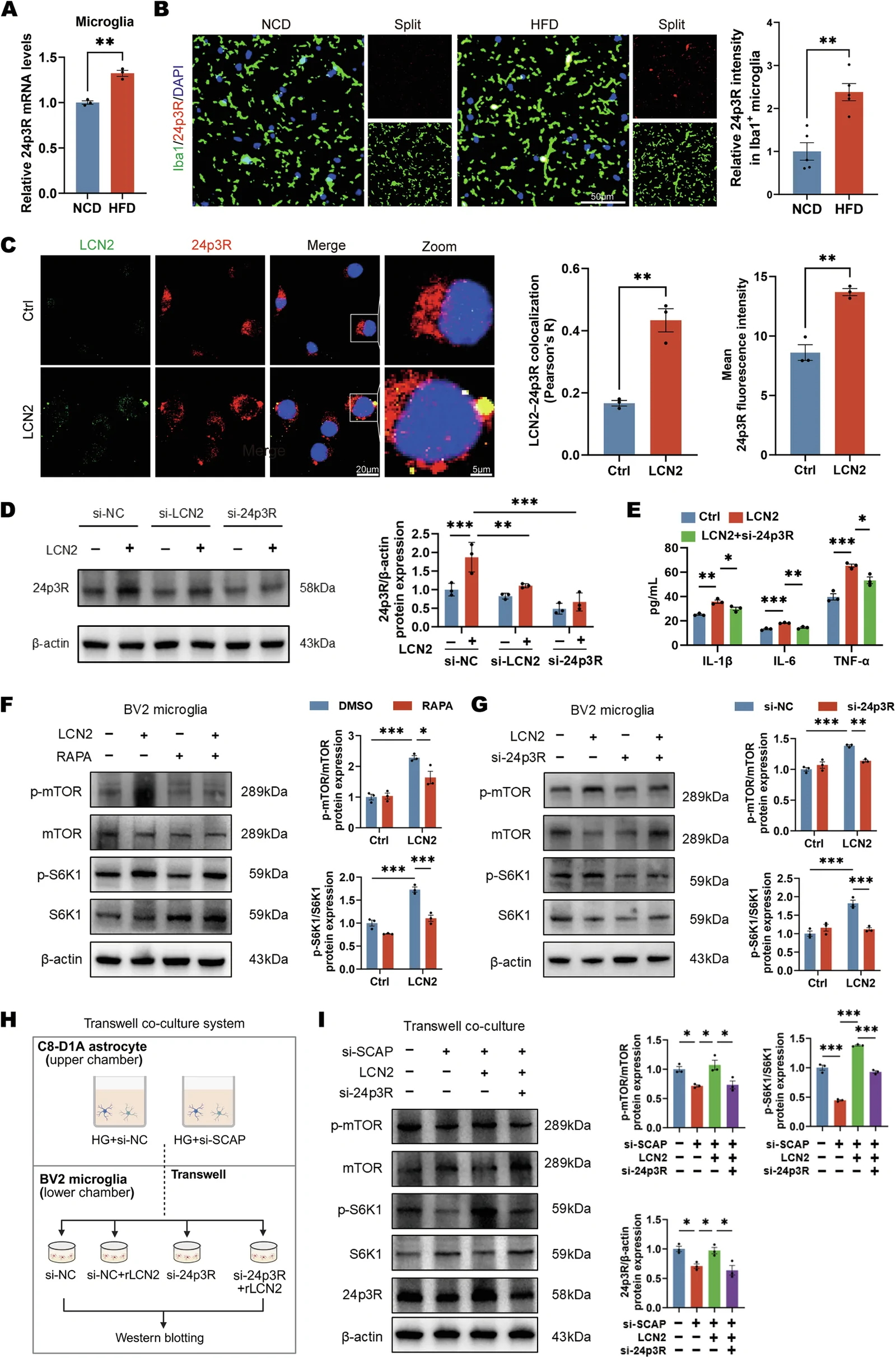
LCN2 activates microglial mTOR signaling via the receptor 24p3R. **A** RT–qPCR analysis of MACS-isolated hippocampal microglia showing 24p3R mRNA expression in HFD-fed mice and NCD controls (*n* = 3 per group). **B** Representative immunofluorescence images of hippocampal sections stained for Iba1 (green) and 24p3R (red), with DAPI labeling nuclei (blue). Quantification of 24p3R fluorescence intensity in Iba1⁺ microglia is shown (*n* = 5 per group). Scale bar, 50 μm. **C** Representative confocal images of BV2 microglia treated with recombinant LCN2 (rLCN2), stained for LCN2 (green) and 24p3R (red), with DAPI labeling nuclei (blue). Quantification of colocalization (Pearson’s correlation coefficient) and 24p3R mean fluorescence intensity is shown (*n* = 3 independent biological replicates per group). Scale bar, 20 μm (overview) and 5 μm (enlarged images). **D** Representative Western blot images and quantification of 24p3R protein expression in BV2 microglia transfected with si-NC, si-LCN2, or si-24p3R, followed by stimulation with rLCN2 (*n* = 3 independent biological replicates per group). **E** ELISA quantification of IL-1β, IL-6, and TNF-α levels in microglial culture supernatants under the indicated treatments (*n* = 3 independent biological replicates per group). **F** Representative Western blot images and quantification of p-mTOR/mTOR and p-S6K1/S6K1 in BV2 microglia treated with rLCN2 in the presence or absence of rapamycin (RAPA) (*n* = 3 independent biological replicates per group). **G** Representative Western blot images and quantification of p-mTOR/mTOR and p-S6K1/S6K1 in BV2 microglia transfected with si-NC or si-24p3R under rLCN2 treatment (*n* = 3 independent biological replicates per group). **H** Schematic illustration of the Transwell co-culture system used to investigate astrocyte–microglia interactions. C8-D1A astrocyte cells in the upper chamber were cultured under high-glucose (HG) conditions and transfected with si-NC or si-SCAP. BV2 microglial cells in the lower chamber were transfected with si-NC or si-24p3R and treated with or without rLCN2. Created in BioRender (https://BioRender.com/x0ibpjw). **I** Representative Western blot images and quantification of 24p3R, p-mTOR/mTOR, and p-S6K1/S6K1 in the co-culture system (*n* = 3 independent biological replicates per group). Data are presented as mean ± SEM. Statistical analyses were performed using unpaired two-tailed Student’s *t*-test (**A**–**C**), one-way ANOVA followed by appropriate post hoc tests (**E**, **I**), or two-way ANOVA with post hoc tests (**D**, **F**, **G**).

To define how LCN2 may signal to microglia under diabetic conditions, we first examined the expression of previously reported LCN2 receptor candidates in the hippocampus. RT–qPCR analysis showed a significant upregulation of the LCN2 receptor 24p3R in the hippocampus of HFD-fed mice, whereas the expression of MC4R remained unchanged (Fig. S9A). Cell type–specific mRNA analyses of MACS-isolated cells further showed that 24p3R was predominantly upregulated in microglia, with no significant changes in astrocytes (Figs. 7A and S9B). Consistently, immunofluorescence analysis showed increased 24p3R fluorescence intensity in Iba1⁺ microglia of HFD-fed mice (Fig. 7B), whereas 24p3R levels in GFAP⁺ astrocytes remained unchanged (Fig. S9C).

In vitro analyses further demonstrated that rLCN2 enhanced the co-localization of LCN2 and 24p3R in BV2 microglia, accompanied by increased 24p3R fluorescence intensity (Fig. 7C). rLCN2 increased 24p3R protein abundance, which was attenuated by LCN2 knockdown and abolished by 24p3R silencing (Fig. 7D). Functionally, 24p3R knockdown significantly reduced rLCN2-induced secretion of IL-1β, IL-6, and TNF-α (Fig. 7E), supporting a role for 24p3R in mediating LCN2-dependent microglial activation.

To further characterize downstream signaling events associated with LCN2–24p3R activation, untargeted LC–MS/MS metabolomic profiling was performed. Among 210 significantly altered metabolites, glycerophospholipid species, including phosphatidylcholines (PC), phosphatidylethanolamines (PE), and phosphatidic acid (PA), were prominently increased (Fig. S10A–G). KEGG enrichment analysis highlighted pathways related to amino acid metabolism, lipid metabolism, and cellular signaling, with notable enrichment of the mTOR pathway (Fig. S10H). This enrichment, together with the established role of mTORC1 in coordinating metabolic reprogramming and inflammatory activation [34, 35], prompted us to further examine whether LCN2 activates mTOR signaling. rLCN2 increased the phosphorylation levels of mTOR and its downstream effector S6K1, which was suppressed by RAPA (Fig. 7F). Consistently, RAPA attenuated rLCN2-induced inflammatory gene expression and LD accumulation (Fig. S11A, B), indicating that mTOR contributes to downstream inflammatory and metabolic responses. Importantly, knockdown of 24p3R reduced rLCN2-induced phosphorylation of mTOR and S6K1 (Fig. 7G), indicating that 24p3R is required for coupling extracellular LCN2 signals to intracellular mTOR activation in microglia.

A Transwell co-culture system was further employed to validate this mechanism (Fig. 7H). Silencing SCAP in astrocytes reduced mTOR signaling activity in microglia, as evidenced by decreased phosphorylation of mTOR and S6K1, whereas rLCN2 supplementation largely restored this activation. In contrast, knockdown of 24p3R in microglia abrogated rLCN2-induced mTOR pathway activation (Fig. 7I), supporting a critical role for 24p3R in mediating LCN2-dependent signaling. Collectively, these findings support a model in which astrocytic SCAP promotes LCN2-dependent paracrine activation of microglial 24p3R, leading to mTOR pathway activation and thereby contributing to inflammatory activation and lipid-associated metabolic alterations.

### Pharmacological blockade of LCN2 or inhibition of mTOR alleviates neuroinflammation, neuronal injury, and cognitive deficits in HFD-diabetic mice

**Fig. 8:**
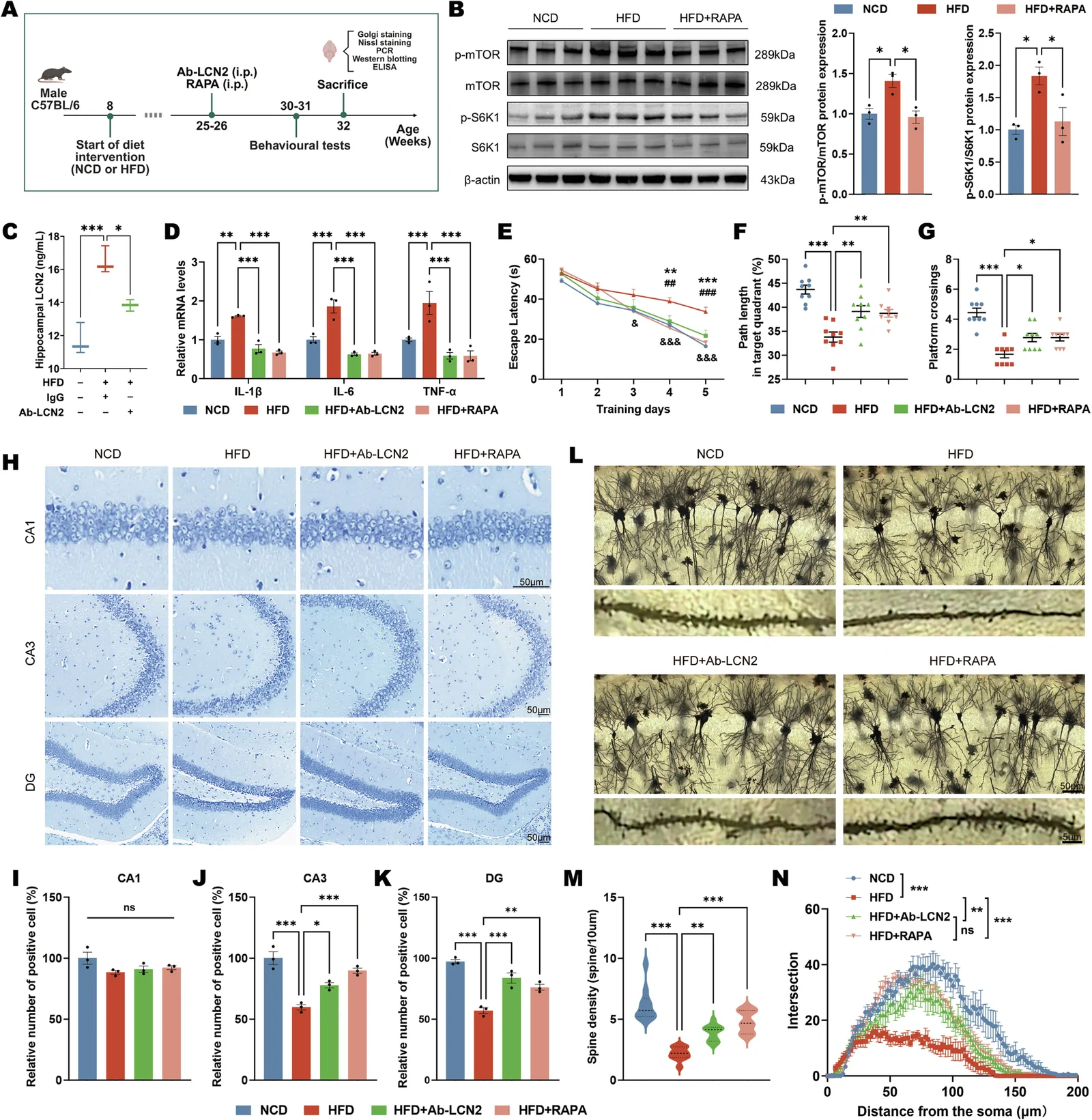
Targeting LCN2 or mTOR signaling ameliorates neuroinflammation, neuronal injury, and cognitive deficits in HFD-diabetic mice. **A** Schematic overview of the in vivo experimental design. The indicated groups include NCD, HFD, HFD+Ab-LCN2, and HFD + RAPA mice. Created in BioRender (https://BioRender.com/c62q79r). **B** Representative Western blot images and quantification of phosphorylated mTOR (p-mTOR), total mTOR, phosphorylated S6K1 (p-S6K1), and total S6K1 in hippocampal lysates from the indicated groups. Quantification of p-mTOR/mTOR and p-S6K1/S6K1 ratios is shown on the right (*n* = 3 mice per group). **C** ELISA quantification of hippocampal LCN2 protein levels (*n* = 3 mice per group). **D** RT-qPCR analysis of IL-1β, IL-6, and TNF-α mRNA expression in hippocampal tissues (*n* = 3 mice per group). Morris water maze (MWM) performance in the indicated groups, including **E** escape latency during acquisition training, **F** percentage of path length in the target quadrant, and **G** number of platform crossings in the probe trial (*n* = 9 mice per group). **H** Representative Nissl staining images of hippocampal CA1, CA3, and DG regions from the indicated groups. Scale bar, 50 μm. Quantification of the relative number of Nissl-positive cells in **I** CA1, **J** CA3, and **K** DG (*n* = 3 mice per group). **L** Representative Golgi staining images of CA1 pyramidal neurons, including higher-magnification views of dendritic spines. Scale bars, 50 μm (overview) and 5 μm (spine images). **M** Quantification of dendritic spine density (spines per 10 μm) in CA1 pyramidal neurons. **N** Sholl analysis of dendritic complexity in CA1 pyramidal neurons (*n* = 3 mice per group; 9–15 neurons analyzed per group). Data are presented as mean ± SEM. Statistical analyses were performed using one-way ANOVA followed by appropriate post hoc tests (**B**–**D**, **F**, **G**, **I**–**M**) or two-way ANOVA followed by appropriate post hoc tests (**E**, **N**). * indicates comparison between NCD and HFD; # indicates comparison between HFD and HFD+Ab-LCN2; & indicates comparison between HFD and HFD + RAPA. ^*^P < 0.05, ^**^P < 0.01, ^***^P < 0.001; ns, not significant.

To determine whether pharmacological interruption of the LCN2–mTOR axis can ameliorate neuroinflammation and cognitive deficits in vivo, we evaluated the therapeutic effects of LCN2 blockade by systemic administration of an anti-LCN2 antibody (Ab-LCN2) or RAPA in HFD-induced diabetic mice according to the experimental timeline shown in Fig. 8A. Consistent with effective pathway modulation, hippocampal mTOR signaling was markedly activated in HFD-fed mice, as evidenced by increased phosphorylation of mTOR and its downstream effector S6K1. RAPA administration significantly reduced both the p-mTOR/mTOR and p-S6K1/S6K1 ratios in hippocampal tissue, confirming efficient inhibition of mTOR signaling in vivo (Fig. 8B). In parallel, Ab-LCN2 treatment significantly reduced detectable hippocampal LCN2 protein levels, as measured by ELISA of hippocampal homogenates (Fig. 8C), indicating effective modulation of LCN2 in vivo. Therapeutic benefits were accompanied by reduced neuroinflammatory responses. Hippocampal mRNA levels of the pro-inflammatory cytokines IL-1β, IL-6, and TNF-α were significantly reduced in both treatment groups relative to HFD controls (Fig. 8D), indicating attenuated neuroinflammatory responses. Both interventions also improved hippocampal-dependent cognitive performance. In the MWM, Ab-LCN2– and RAPA-treated HFD mice exhibited shortened escape latencies during training, an increased percentage of path length in the target quadrant, and a higher number of platform crossings in the probe trial compared with untreated HFD controls (Fig. 8E–G), indicating improved spatial learning and memory.

Histopathological analyses further supported neuronal protection. Nissl staining revealed reduced numbers of Nissl-positive neurons and weakened Nissl staining in the CA3 and DG regions of HFD mice, both of which were partially reversed by Ab-LCN2 or RAPA treatment (Fig. 8H–K). In parallel, Golgi staining demonstrated that HFD-induced reductions in dendritic complexity and spine density of CA1 pyramidal neurons were significantly restored following both interventions (Fig. 8L–N). Collectively, these findings demonstrate that pharmacological blockade of LCN2 or inhibition of mTOR signaling alleviates neuroinflammation, neuronal injury, and cognitive deficits in diabetic mice, supporting the functional relevance of the LCN2–mTOR pathway in DACI.

## Discussion

**Fig. 9:**
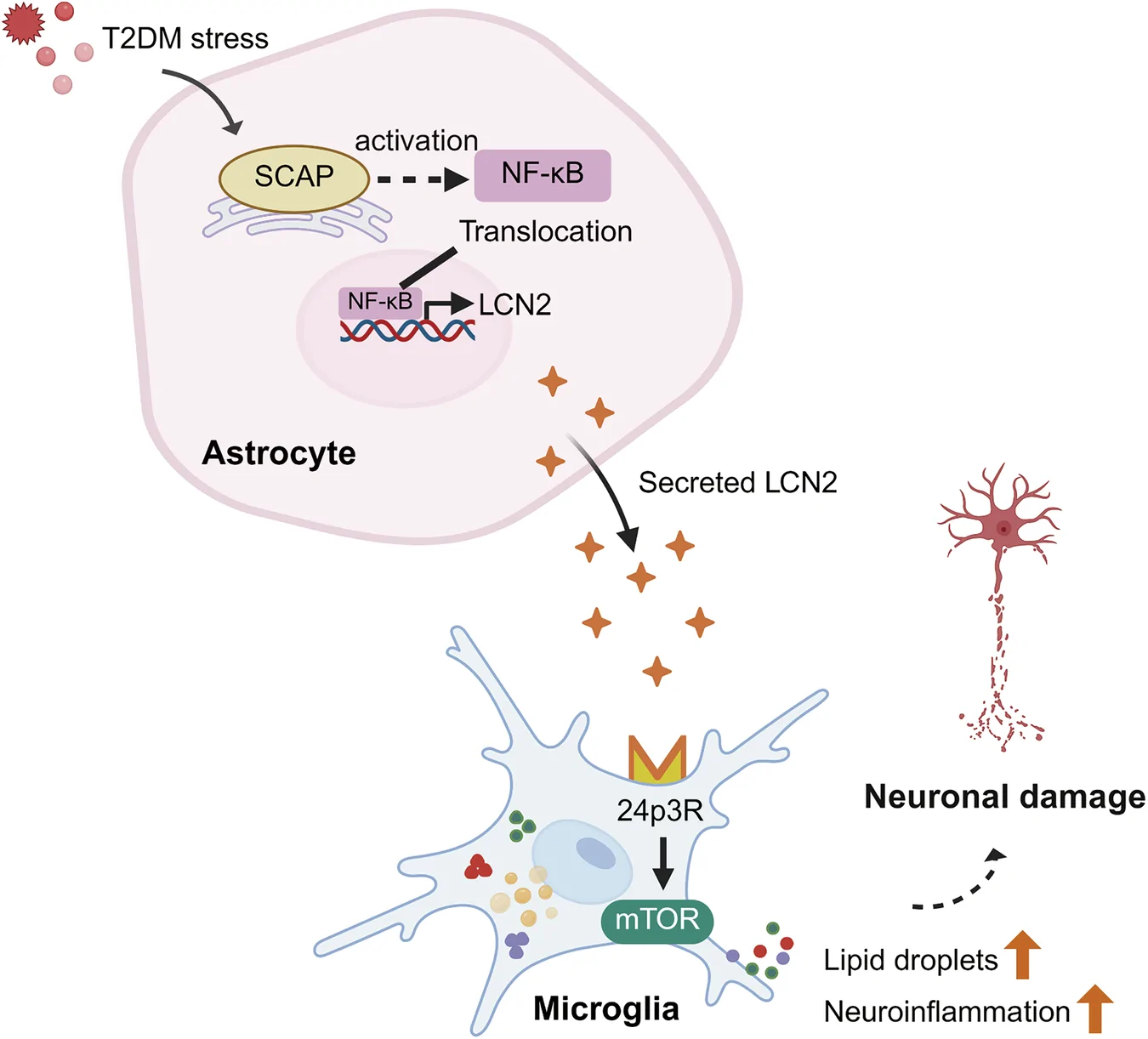
Schematic illustration of the proposed mechanism by which astrocytic SCAP regulates microglial neuroinflammation and lipid droplet accumulation via the LCN2–24p3R–mTOR axis. Under HFD-induced diabetic conditions, SCAP in astrocytes promotes activation of NF-κB signaling, thereby enhancing LCN2 expression and secretion. Secreted LCN2 binds to 24p3R on microglia, leading to activation of mTOR signaling. This signaling cascade promotes microglial pro-inflammatory activation and lipid droplet accumulation, ultimately contributing to neuroinflammation and neuronal injury. Together, these findings suggest that the astrocytic SCAP–LCN2–24p3R–mTOR axis represents a key mechanism underlying glial crosstalk in diabetes-associated cognitive impairment. Created in BioRender (https://BioRender.com/f23oh8b).

In this study, we demonstrate that in an HFD-induced T2DM mouse model, SCAP expression is upregulated in hippocampal astrocytes. Importantly, this increase is not evident during the early phase of metabolic stress but emerges during the chronic phase of disease. Astrocyte-specific deletion of SCAP markedly improved hippocampal-dependent learning, attenuated microglial pro-inflammatory activation, and reduced LD accumulation, supporting a critical role for astrocytic SCAP in glial dysfunction during DACI. Mechanistically, our findings identify astrocytic SCAP as a key link between chronic metabolic stress and microglial inflammation, acting through an LCN2-dependent astrocyte–microglia signaling pathway that activates downstream, receptor-mediated mTOR signaling and promotes LD accumulation (Fig. 9). Together, these results highlight astrocytic SCAP as an important upstream modulator of astrocyte–microglia crosstalk under diabetic conditions and underscore its contribution to glial-driven neuroinflammation and neuronal injury in DACI.

SCAP is predominantly localized in the cytoplasm and, therefore, is unlikely to directly regulate gene transcription. Thus, the effects of astrocytic SCAP on LCN2 expression are likely mediated through intracellular signaling pathways rather than direct promoter engagement. LCN2 transcription is known to be positively regulated by NF-κB, which binds to a response element within the LCN2 promoter [32, 33]. Although the precise molecular mechanism linking SCAP to NF-κB activation was not fully delineated in the present study, our recent study suggested that SCAP may associate with the NF-κB inhibitory protein IκBα and the p65 subunit, forming a complex that undergoes ER-to-Golgi trafficking and promotes IκBα phosphorylation, thereby facilitating NF-κB activation in astrocytes [15]. Similarly, a study in non-alcoholic fatty liver disease reported that Golgi-localized SCAP associates with STING and promotes activation of downstream TBK1–NF-κB signaling in macrophages [25].

Building on these findings, our data identify LCN2 as an important astrocyte-derived effector linking SCAP-dependent stress signaling to microglial responses in HFD-induced T2DM mice. LCN2 is a pleiotropic factor secreted by neurons and glial cells that modulates brain function [36]; however, in this study, astrocytic LCN2 emerges as a paracrine signal preferentially acting on neighboring microglia. Although LCN2 expression is low in the normal adult brain, it is markedly elevated in numerous metabolic and CNS disorders [37, 38], consistent with its stress-responsive nature. Because LCN2 can act on multiple neural cell types, an important question raised by our data is how LCN2 released from reactive astrocytes selectively promotes microglial polarization rather than exerting dominant autocrine effects on astrocytes. Our results suggest that this specificity is mediated, at least in part, by the enriched expression of the LCN2 receptor 24p3R on microglia. The LCN2/24p3R axis has been implicated in diverse inflammatory processes and in shaping local immune microenvironments [39]. Recent studies have reported that LCN2/24p3R signaling mediates astrocyte pyroptosis following cerebral ischemia/reperfusion injury through activation of the NLRP3 inflammasome [40]. While previous studies have mainly focused on the direct effects of LCN2 on neurons, the present study highlights a complementary role for LCN2 in astrocyte–microglia crosstalk–mediated neuroinflammation. Supporting the notion that astrocyte–microglia interactions are a common feature of neuroinflammatory diseases, a recent *Cell Reports* study using spatial transcriptomics identified extensive microglia–astrocyte interactions within the amyloid plaque niche in Alzheimer’s disease (AD) [41]. Together, these observations underscore the importance of dynamic glial interactions in the pathogenesis of chronic neuroinflammatory disorders, including conditions associated with metabolic dysfunction, such as DACI.

It should be noted, however, that LCN2 is a pleiotropic ligand capable of engaging multiple receptors in a context-dependent manner. Although our data support 24p3R as a major candidate receptor mediating astrocyte-derived LCN2 signaling in microglia, we cannot exclude the potential contribution of alternative receptors, such as megalin (LRP2), particularly in vivo. Recent work has identified LRP2 as an important functional receptor for LCN2 in oligodendrocytes and has shown that astrocyte-derived LCN2 can contribute to secondary demyelination after stroke via LRP2 signaling [42]. Further studies employing receptor-specific genetic or pharmacological approaches will be required to delineate the relative contributions of distinct receptors to microglial responses under diabetic conditions.

Although astrocytic SCAP deficiency markedly suppressed microglial LD accumulation and neuroinflammation, our data suggest that this effect is not attributable to detectable changes in intrinsic astrocytic lipid storage or classical lipid efflux pathways. The absence of overt lipid alterations in astrocytes likely reflects, at least in part, their distinctive metabolic features: under physiological conditions, astrocytes are generally considered to rely predominantly on glycolysis and glycogen metabolism for energy supply and to exhibit a lower dependence on neutral lipid storage compared with microglia [43]. In addition, astrocytes possess efficient lipid turnover mechanisms—including fatty acid oxidation, exosome-mediated lipid release, and intercellular lipid transfer—which may help buffer potential perturbations in SREBP-dependent lipid regulatory programs and thereby preserve lipid homeostasis even under metabolic stress [44, 45]. Mechanistically, our findings support a model in which astrocytic SCAP functions less as a direct regulator of cellular lipid storage and more as a signaling hub that promotes neuroinflammation through NF-κB–dependent induction of LCN2. Accordingly, rather than directly altering astrocytic lipid content, SCAP deletion influences microglial lipid metabolism and inflammatory activation indirectly by attenuating astrocyte-derived LCN2 signaling. Consistent with this interpretation, exogenous LCN2 was sufficient in our experimental system to induce LD formation, pro-inflammatory microglial polarization, and mTOR activation, further supporting its role in astrocyte–microglia metabolic communication. Although our tissue-level analyses revealed no overt astrocytic lipid changes, subtle region-specific or lipid-species–specific alterations—such as in cholesterol or sphingolipids—cannot be fully excluded. Future studies combining spatially resolved lipidomics with live-cell LD tracing will be valuable for delineating the dynamic intercellular lipid flux and refining the SCAP–LCN2 axis governing glial lipid homeostasis in diabetic neuroinflammation.

Microglial LDs are increasingly recognized as active indicators of metabolic stress and inflammatory dysfunction rather than passive lipid stores. Lipid droplet–accumulating microglia (LDAM) exhibit impaired phagocytosis, elevated reactive oxygen species production, lysosomal dysfunction, and heightened pro-inflammatory signaling [46–48]. Accumulation of LDs has been reported at early stages of neurodegenerative and metabolic brain disorders, including AD and DACI, and is closely associated with cognitive decline [48–50]. Mechanistically, several studies have shown that neutral lipid–rich LDs can function as platforms for the generation of pro-inflammatory lipid mediators and the maintenance of inflammatory signaling, including NF-κB signaling, thereby amplifying neuroinflammation [51]. In this context, the reduction of LDs observed following astrocytic SCAP deletion likely reflects a partial normalization of microglial metabolic and inflammatory states, highlighting LD dynamics as a sensitive indicator of microglial stress in DACI.

Importantly, these LD-associated changes are closely linked to mTOR-dependent metabolic reprogramming, an important regulator of microglial phenotype. mTOR functions as a key metabolic integrator [52], and its dysregulation contributes to diabetic and age-related neuropathology [53–55]. Hyperactive mTOR suppresses autophagy and mitochondrial function, exacerbates amyloid-β (Aβ) and tau pathology [56, 57]. Recent studies have further revealed that microglial immune functions are tightly coupled to intracellular metabolic reprogramming downstream of mTOR signaling. Pharmacological or genetic inhibition of mTOR promotes a shift away from pro-inflammatory activation, accompanied by reduced production of inflammatory mediators and coordinated changes in glycolytic metabolism [52, 58]. Under pathological conditions, such as Aβ exposure or APOE4 expression, activation of the AKT–mTOR–HIF-1α axis drives a metabolic transition from oxidative phosphorylation toward aerobic glycolysis, resulting in impaired autophagic clearance and lipid dysregulation in microglia [59, 60]. In our study, LCN2-driven mTOR activation was associated with enhanced glycerophospholipid metabolism and increased LD accumulation, supporting a model in which mTOR links metabolic overload to inflammatory polarization. The reduction in microglial LD burden observed following astrocyte-specific SCAP deletion or rapamycin treatment likely reflects coordinated alterations in microglial lipid handling, including decreased de novo lipid synthesis and partial restoration of lipophagy. mTOR inhibition is known to reactivate autophagic–lysosomal pathways [61, 62] and restrict neutral-lipid availability for LD biogenesis via mTOR–PPARγ–dependent mechanisms [63]. In addition, some studies have suggested that LD catabolism may also influence neuroimmune responses. For example, inhibition of adipose triglyceride lipase (ATGL)–dependent LD lipolysis in microglia has been reported to attenuate LPS-induced neuroinflammatory responses by limiting the availability of free fatty acids for the generation of lipid-derived inflammatory mediators [64]. Together, these observations support a mechanistic framework in which excessive LD accumulation and defective lipid turnover contribute to microglial dysfunction, with LD dynamics and mTOR activity emerging as critical nodes linking metabolic stress to neuroinflammation in DACI.

Behaviorally, astrocytic SCAP deletion preferentially improved hippocampus-dependent spatial learning in the MWM, whereas performance in working and recognition memory tasks showed only modest, non-significant improvement. This task-dependent behavioral profile is consistent with the distinct neural circuit requirements of these tasks, as well as with the hippocampus-centered neuroinflammatory and glial alterations observed in our HFD-induced diabetic mice [65–67]. From a translational perspective, targeting the astrocytic SCAP–LCN2 axis attenuated microglial LD accumulation and neuroinflammation, in line with preclinical evidence that SCAP inhibition or LCN2 neutralization alleviates metabolic and inflammatory disturbances [68, 69]. However, given SCAP’s important role in systemic lipid homeostasis, global SCAP inhibition carries potential risks, including hepatic dyslipidemia and impaired steroidogenesis [25, 28, 70]. Within the CNS, non-selective SCAP suppression may also disrupt synaptic maintenance and myelination [13]. These considerations underscore the need for CNS- and cell type–restricted therapeutic strategies, such as brain-targeted nanodelivery or promoter-restricted gene modulation [71], and suggest that downstream targeting of LCN2 or its receptor 24p3R may represent a potentially safer and more tractable alternative.

Several limitations should be considered. First, although LCN2/24p3R signaling appears to act upstream of mTORC1 activation, the intermediate molecular events remain undefined. Additionally, pharmacological mTOR inhibition in vivo is unlikely to be microglia-specific. Future studies employing microglia-restricted genetic manipulation of mTOR pathway components or cell-type–resolved analyses of mTOR activity will be needed to definitively delineate cell-specific contributions. Second, while the involvement of microglial 24p3R in mediating astrocyte-derived LCN2 effects has been suggested, it has not yet been genetically validated in vivo. Third, although LD accumulation strongly correlates with microglial activation, establishing causal relationships will require direct manipulation of LD biogenesis or turnover. Moreover, gain-of-function rescue experiments reintroducing LCN2 into SCAP-deficient astrocytes have not been performed in vivo and would provide a more stringent test of pathway hierarchy. Finally, the exclusive use of male mice limits the generalizability of our findings. Estrogen has been reported to influence NF-κB activity and LCN2 expression in glial cells [72–74], suggesting potential sex-dependent regulation of the SCAP–LCN2 pathway. Future studies could include both male and female subjects and incorporate hormonal manipulation paradigms (such as ovariectomy and estrogen replacement) to systematically evaluate how endocrine regulation shapes activation of the SCAP–LCN2–mTOR signaling axis and its pathological consequences.

In conclusion, this study identifies an astrocyte-to-microglia communication pathway in which astrocytic SCAP promotes NF-κB–dependent LCN2 expression and secretion. Astrocyte-derived LCN2 subsequently engages its cognate receptor 24p3R on microglia, leading to mTOR activation, metabolic reprogramming, and neuroinflammation under diabetic conditions. Together, these findings provide mechanistic insight into how diabetic metabolic stress drives glial dysfunction in DACI and establish the astrocytic SCAP–LCN2 signaling axis as a conceptual framework for developing targeted strategies to ameliorate diabetes-related neuroinflammation and cognitive decline.

## Supplementary information

## Data Availability

The RNA sequencing data generated in this study have been deposited in the Genome Sequence Archive (GSA) under accession number CRA040607. All other data supporting the findings of this study are available within the article and its Supplementary Materials or from the corresponding author upon reasonable request.
