# Supplementary material for: Astrocytic SCAP deletion ameliorates diabetes-associated cognitive impairment by suppressing microglial neuroinflammation and lipid droplet accumulation via the LCN2–24p3R–mTOR axis
Source: Cell Death Dis. 2026 Jun 22;17(1):769. doi: 10.1038/s41419-026-09019-y (PMC13538556; doi:10.1038/s41419-026-09019-y)
Supplement: Supplementary file 1 [file 41419_2026_9019_MOESM3_ESM.pdf]

Corresponding Author Name: Yang Yuan
Manuscript Number: CDDIS-25-542?

SPRINGER NATURE

Reporting Summary

Springer Nature wishes to improve the reproducibility of the work that we publish. This checklist is used to ensure good reporting standards and to improve the reproducibility. Please respond completely to all questions relevant to your manuscript. For more information, please read the journal's Guide to Authors.

☑ **Check here to confirm that the following information is available in the Material & Methods section:**

- **The exact sample size (*n*)** for each experimental group/condition, given as a number, not a range
- **A description of the sample collection** allowing the reader to understand whether the samples represent technical or biological replicates (including how many animals, litters, culture, etc.)
- **A statement of how many times the experiment shown was replicated in the laboratory**
- **Definitions of statistical methods and measures**: For small sample sizes (n<5) descriptive statistics are not appropriate, instead plot individual data points
  - Very common tests, such as *t*-test, simple χ^2 tests, Wilcoxon and Mann-Whitney tests, can be unambiguously identified by name only, but more complex techniques should be described in the methods section
  - Are tests one-sided or two-sided?
  - Are there adjustments for multiple comparisons?
  - **Statistical test results**, e.g., ***P* values**
  - Definition of **'center values'** as **median or mean**;
  - Definition of **error bars as s.d. or s.e.m. or c.i.**

Please ensure that the answers to the following questions are reported in the manuscript itself. We encourage you to include a specific subsection in the methods section for statistics, reagents and animal models. Below, provide the page number or section and paragraph number.

Statistics and general methods

	**Reported in section/paragraph or page #**
1. How was the sample size chosen to ensure adequate power to detect a pre-specified effect size? (Give section/paragraph or page #)	Sample sizes were determined based on previous studies in the field. No statistical methods were used to pre-determine sample size.
For animal studies, include a statement about sample size estimate even if no statistical methods were used.	Methods section
2. Describe inclusion/exclusion criteria if samples or animals were excluded from the analysis. Were the criteria pre-established? (Give section/paragraph or page #)	No data were excluded in this study.
3. If a method of randomization was used to determine how samples/animals were allocated to experimental groups and processed, describe it. (Give section/paragraph or page #)	Animals were randomly assigned to experimental groups prior to the initiation of the experiments. For in vitro experiments, samples were allocated to treatment conditions in a random manner whenever applicable.
For animal studies, include a statement about randomization even if no randomization was used.	Methods section

4. If the investigator was blinded to the group allocation during the experiment and/or when assessing the outcome, state the extent of blinding. (Give section/paragraph or page #)	Investigators were blinded to group allocation during outcome assessment and data analysis whenever feasible. Behavioral testing and quantitative analyses were performed by investigators blinded to the experimental groups
For animal studies, include a statement about blinding even if no blinding was done.	Methods section
5. For every figure, are statistical tests justified as appropriate?	Statistical tests were selected based on the experimental design and data distribution and were appropriate for each figure
Do the data meet the assumptions of the tests (e.g., normal distribution)?	Data distribution was assessed for normality prior to analysis.
Is there an estimate of variation within each group of data?	Methods, Statistical analysis section
Is the variance similar between the groups that are being statistically compared? (Give section/paragraph or page #)	Methods, Statistical analysis section

Reagents

	**Reported in section/paragraph or page #**
6. Report the source of antibodies (vendor and catalog number)	Methods section and Supplementary Table S4
7. Identify the source of cell lines and report if they were recently authenticated (e.g., by STR profiling) and tested for mycoplasma contamination	Methods section

Animal Models

	**Reported in section/paragraph or page #**
8. Report species, strain, sex and age of animals	Methods, Animal models
9. For experiments involving live vertebrates, include a statement of compliance with ethical regulations and identify the committee(s) approving the experiments.	Methods, Animal models

10. We recommend consulting the ARRIVE guidelines (*PLoS Biol.* **8**(6), e1000412,2010) to ensure that other relevant aspects of animal studies are adequately reported.

Human subjects

	Reported in section/paragraph or page #
11. Identify the committee(s) approving the study protocol.	N/A
12. Include a statement confirming that informed consent was obtained from all subjects.	N/A
13. For publication of patient photos, include a statement confirming that consent to publish was obtained.	N/A
14. Report the clinical trial registration number (at ClinicalTrials.gov or equivalent).	N/A

15. For phase II and III randomized controlled trials, please refer to the CONSORT statement and submit the CONSORT checklist with your submission.

16. For tumor marker prognostic studies, we recommend that you follow the REMARK reporting guidelines.

Data deposition

	Reported in section/paragraph or page #
17. Provide accession codes for deposited data. Data deposition in a public repository is mandatory for:<br>a. Protein, DNA and RNA sequences<br>b. Macromolecular structures<br>c. Crystallographic data for small molecules<br>d. Microarray data	Data availability statement

Deposition is strongly recommended for many other datasets for which structured public repositories exist; more details on our data policy are available in the Guide to Authors. We encourage the provision of other source data in supplementary information or in unstructured repositories such as Figshare and Dryad. We encourage publication of Data Descriptors (see Scientific Data) to maximize data reuse.

18. If computer code was used to generate results that are central to the paper's conclusions, include a statement in the Methods section under "**Code availability"** to indicate whether and how the code can be accessed. Include version information as necessary and any restrictions on availability.	Not applicable
