# Supplementary material for: Astrocytic SCAP deletion ameliorates diabetes-associated cognitive impairment by suppressing microglial neuroinflammation and lipid droplet accumulation via the LCN2–24p3R–mTOR axis
Source: Cell Death Dis. 2026 Jun 22;17(1):769. doi: 10.1038/s41419-026-09019-y (PMC13538556; doi:10.1038/s41419-026-09019-y)

Figure 1G

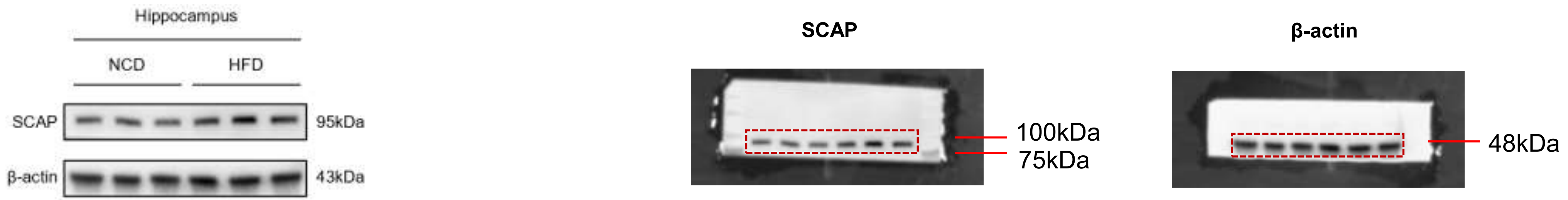

Figure 4E

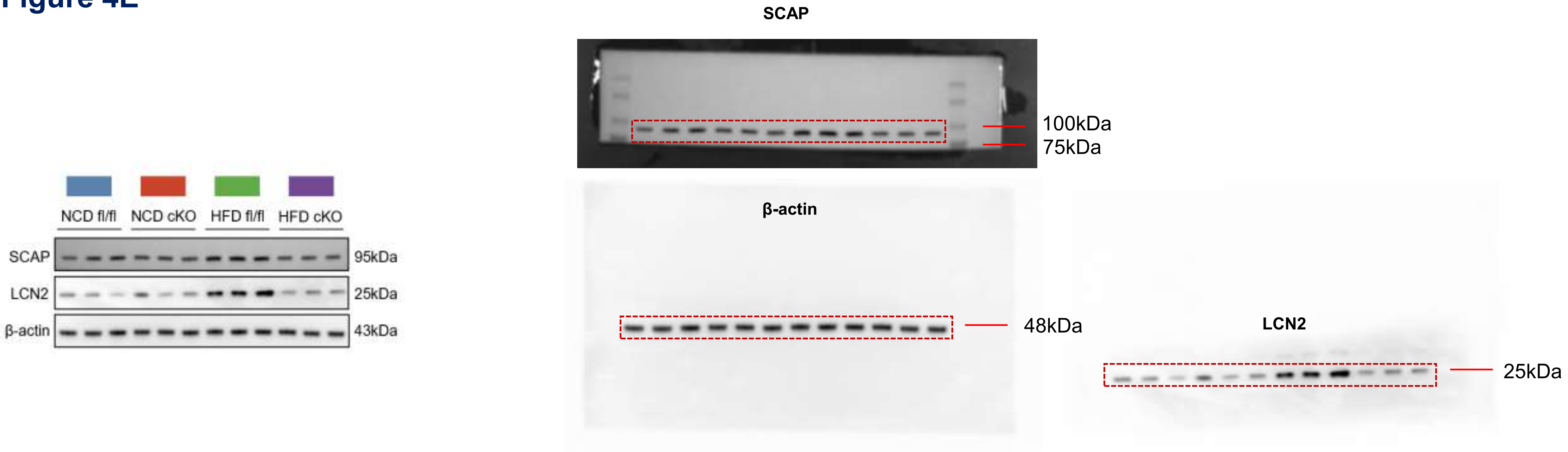

Figure 4L

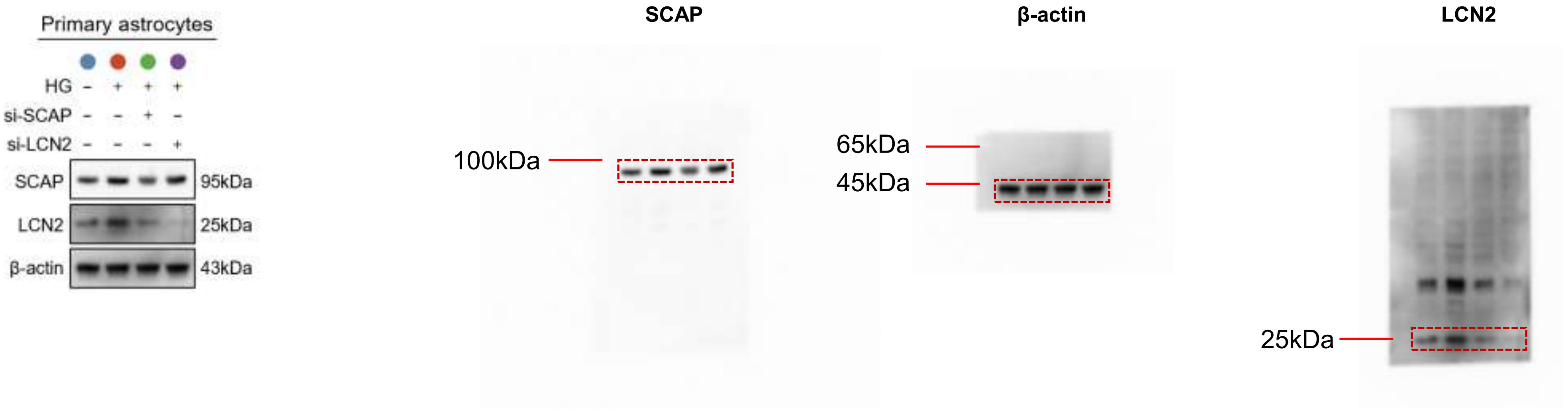

Figure 5E

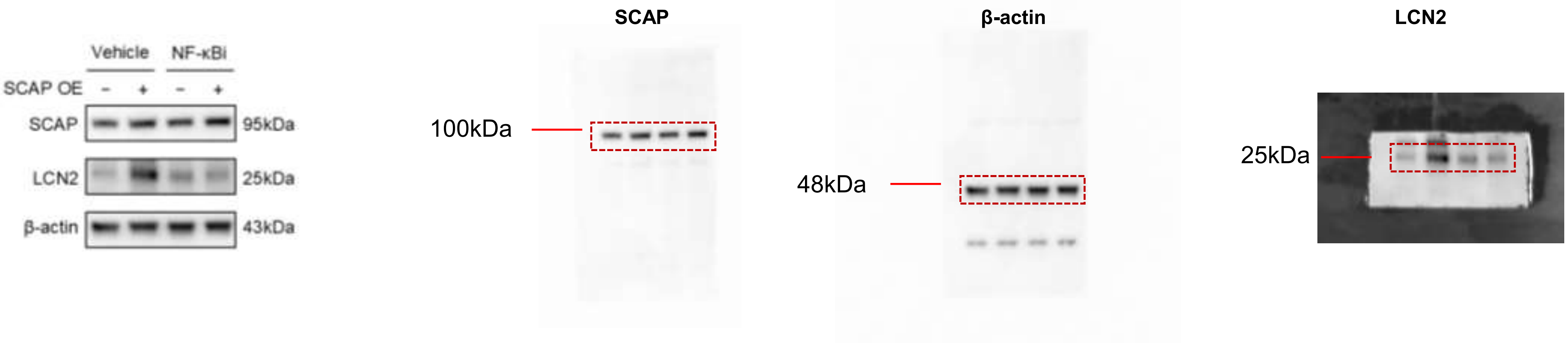

Figure 5G

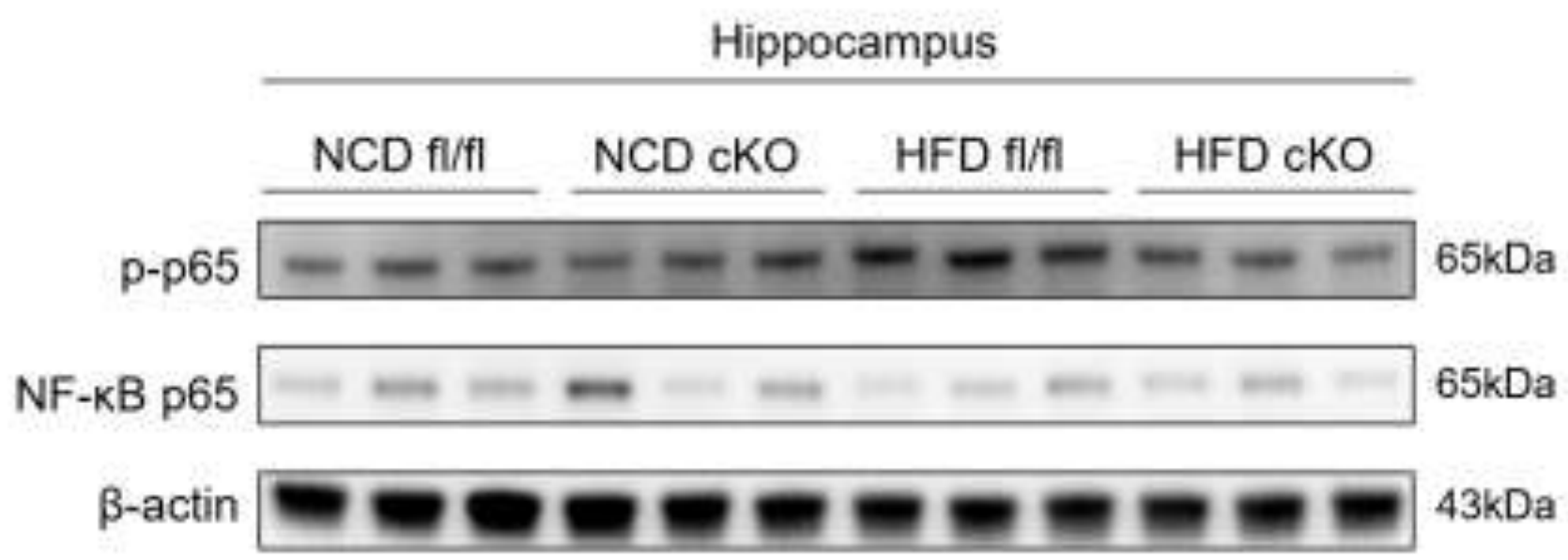
Hippocampus
NCD fl/fl
NCD cKO
HFD fl/fl
HFD cKO
p-p65
NF-κB p65
β-actin
65kDa
65kDa
43kDa

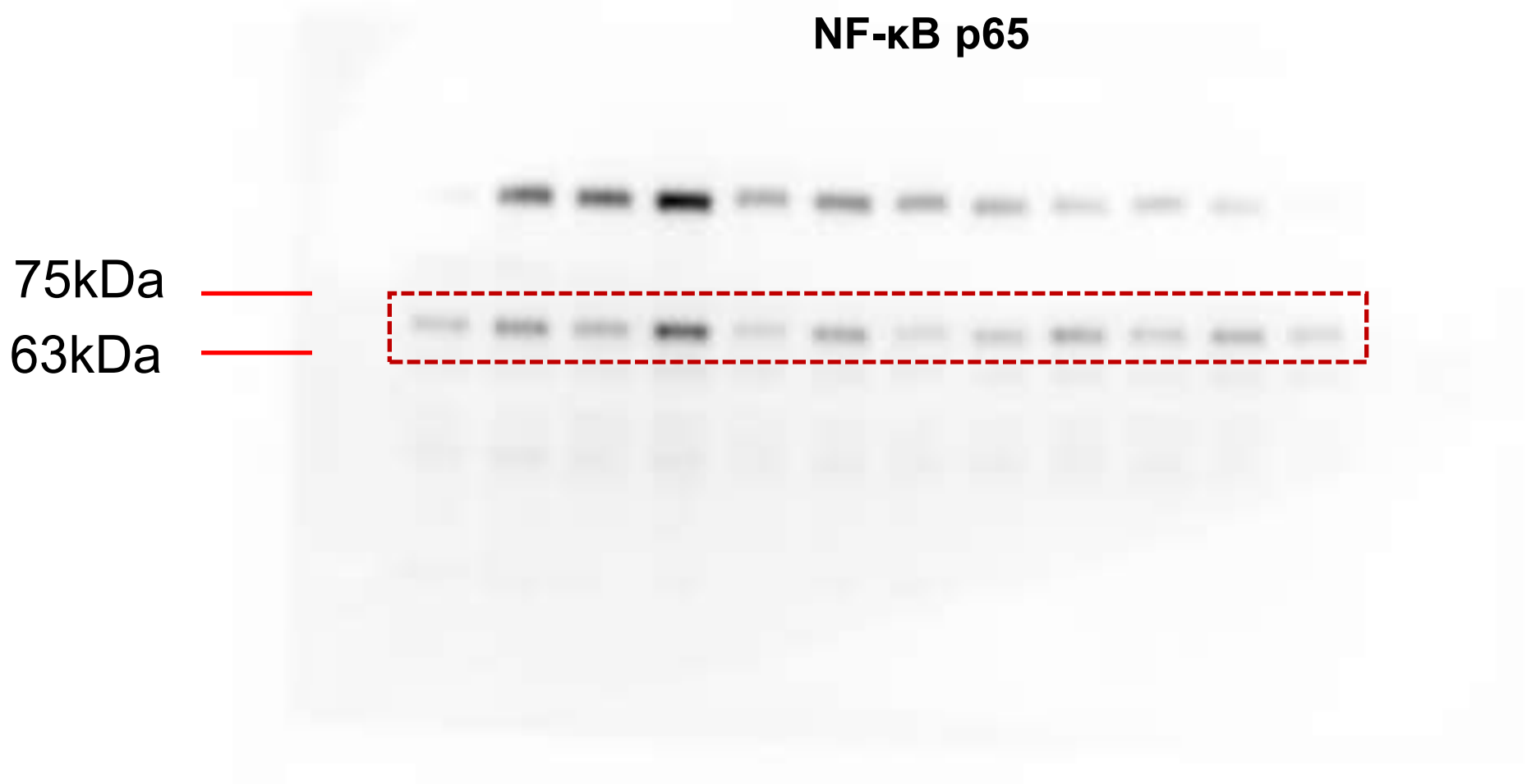
NF-κB p65
75kDa
63kDa

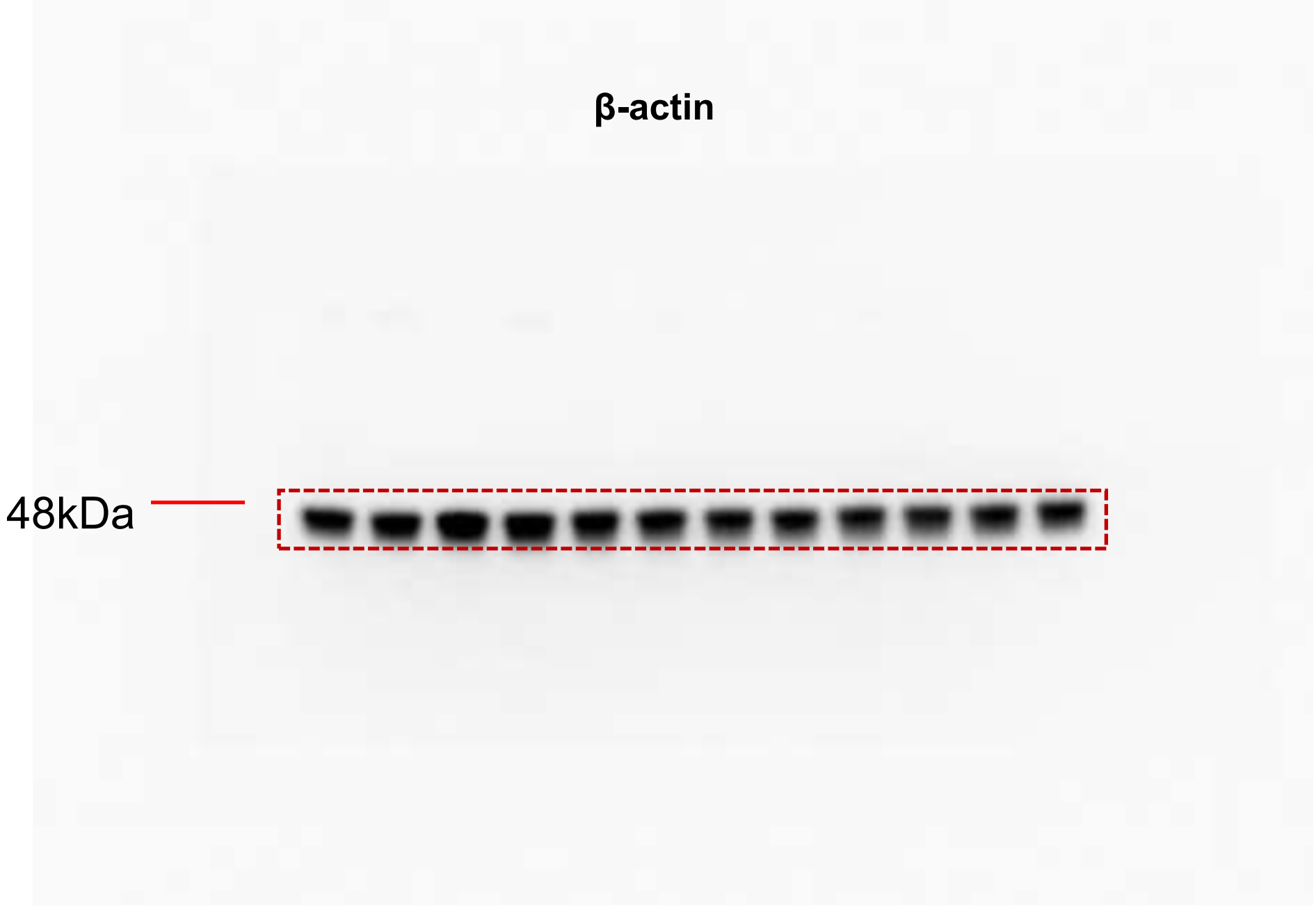
β-actin
48kDa

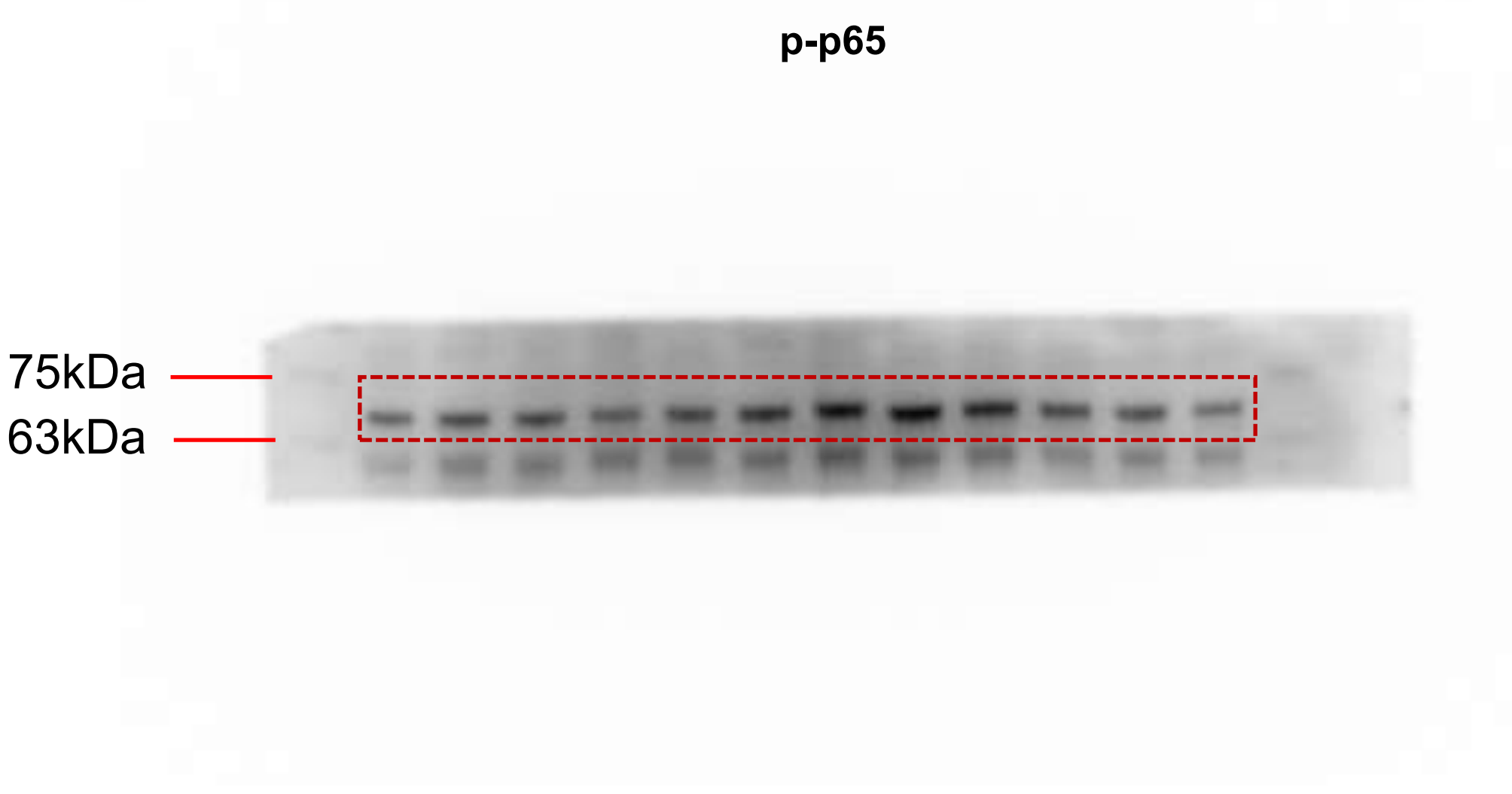
p-p65
75kDa
63kDa

Figure 5H

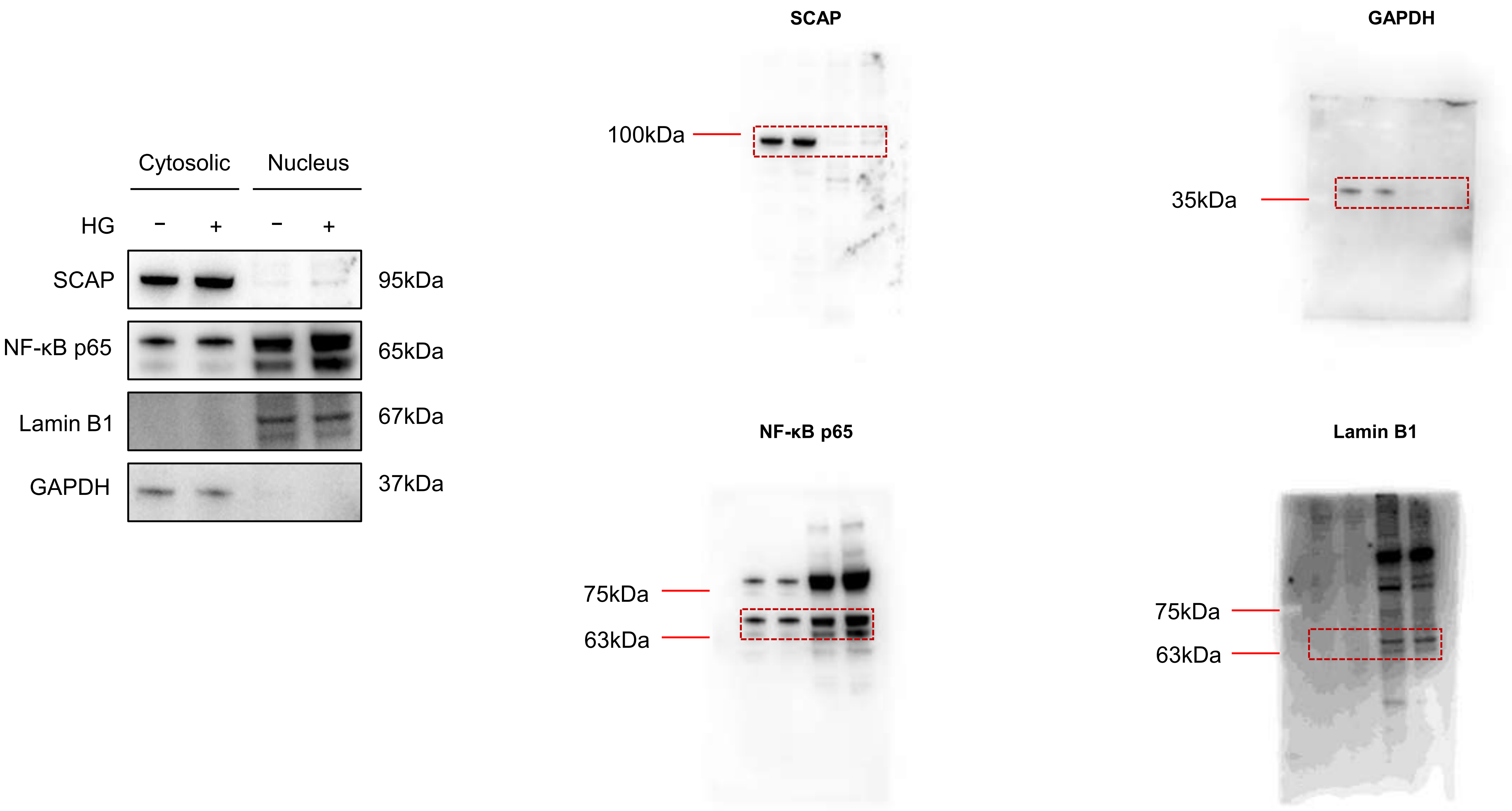

Cytosolic
Nucleus
HG
−
+
−
+
SCAP
95kDa
NF-κB p65
65kDa
Lamin B1
67kDa
GAPDH
37kDa
SCAP
100kDa
GAPDH
35kDa
NF-κB p65
75kDa
63kDa
Lamin B1
75kDa
63kDa

Figure 5I

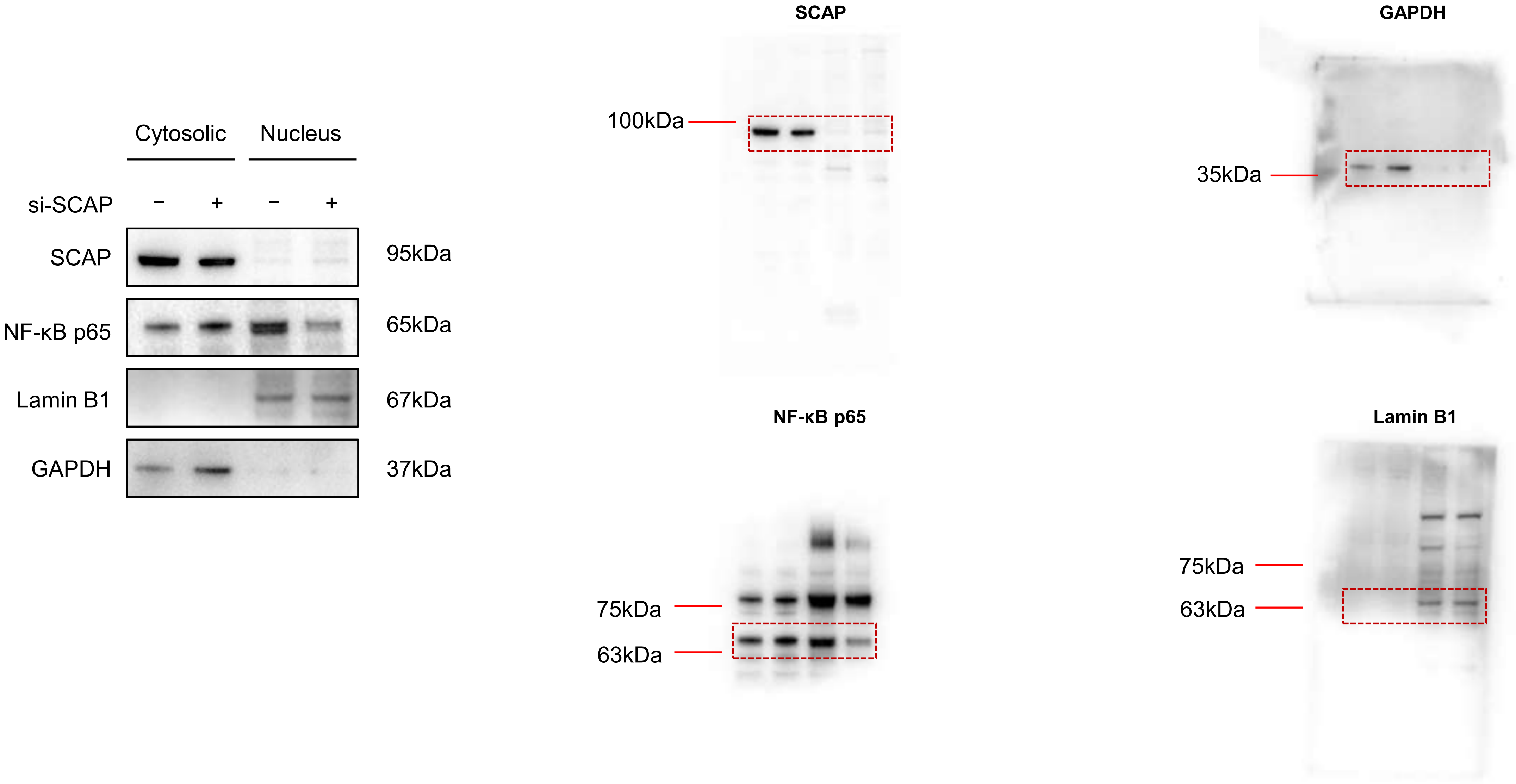

Cytosolic
Nucleus
si-SCAP
−
+
−
+
SCAP
NF-κB p65
Lamin B1
GAPDH
95kDa
65kDa
67kDa
37kDa
SCAP
100kDa
GAPDH
35kDa
NF-κB p65
75kDa
63kDa
Lamin B1
75kDa
63kDa

Figure 6B

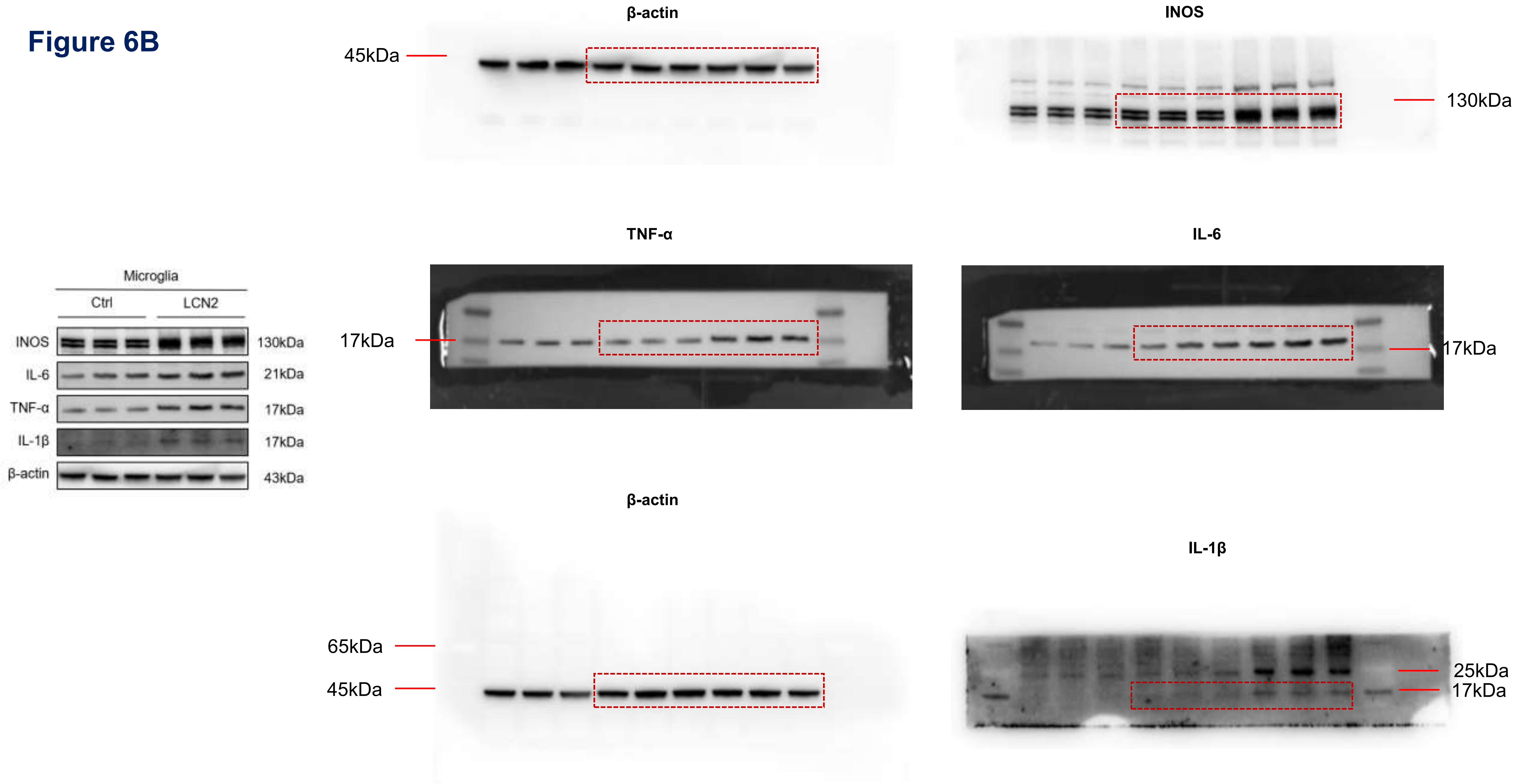

β-actin
45kDa
INOS
130kDa
Microglia
Ctrl
LCN2
INOS
IL-6
TNF-α
IL-1β
β-actin
130kDa
21kDa
17kDa
17kDa
43kDa
TNF-α
17kDa
IL-6
17kDa
β-actin
65kDa
45kDa
IL-1β
25kDa
17kDa

Figure 6E

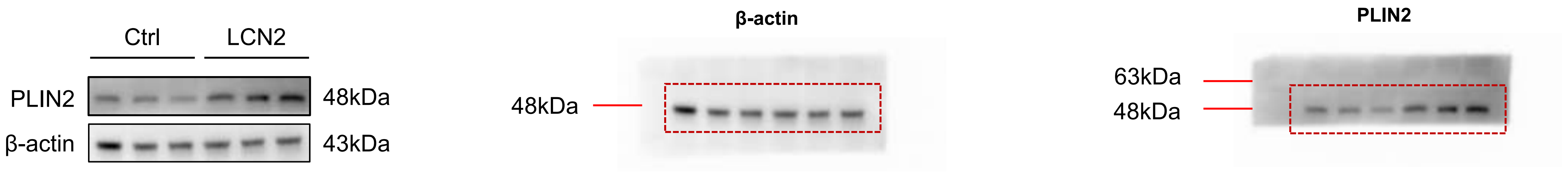

Figure 6H

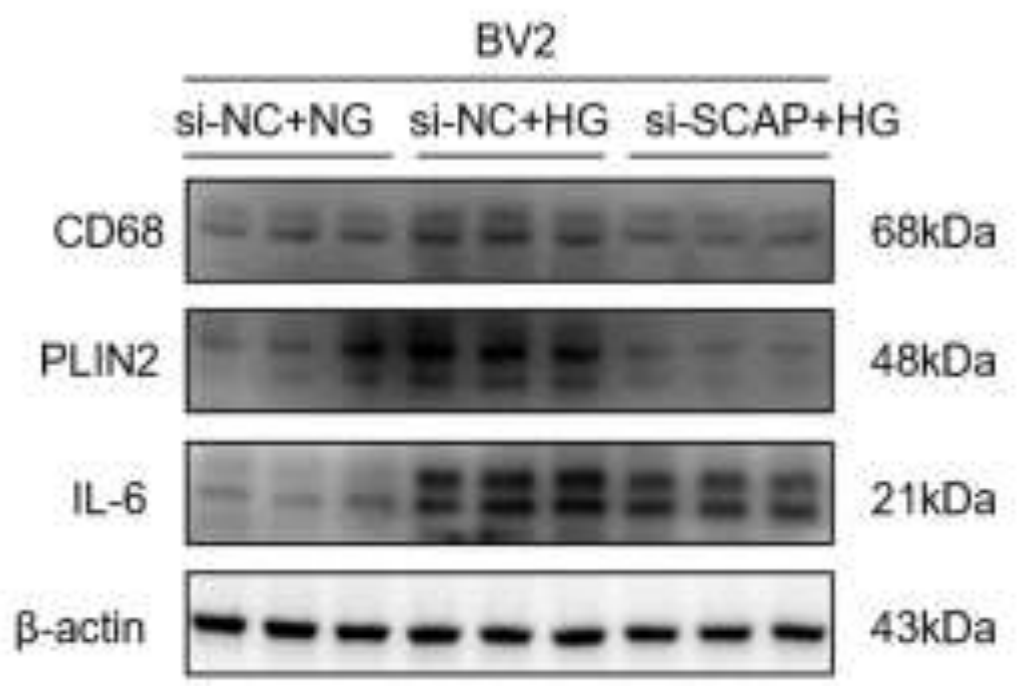

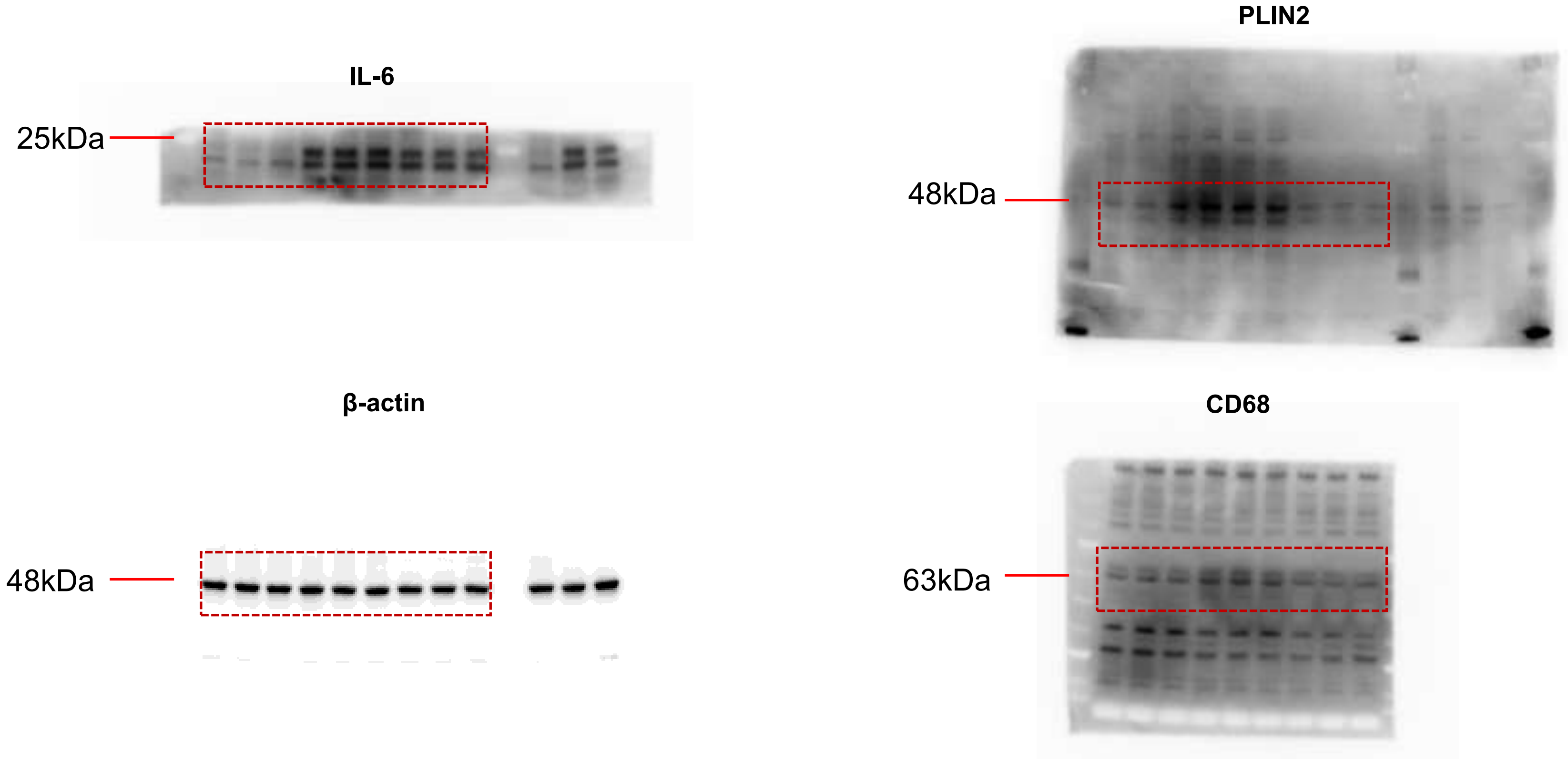

Figure 6I

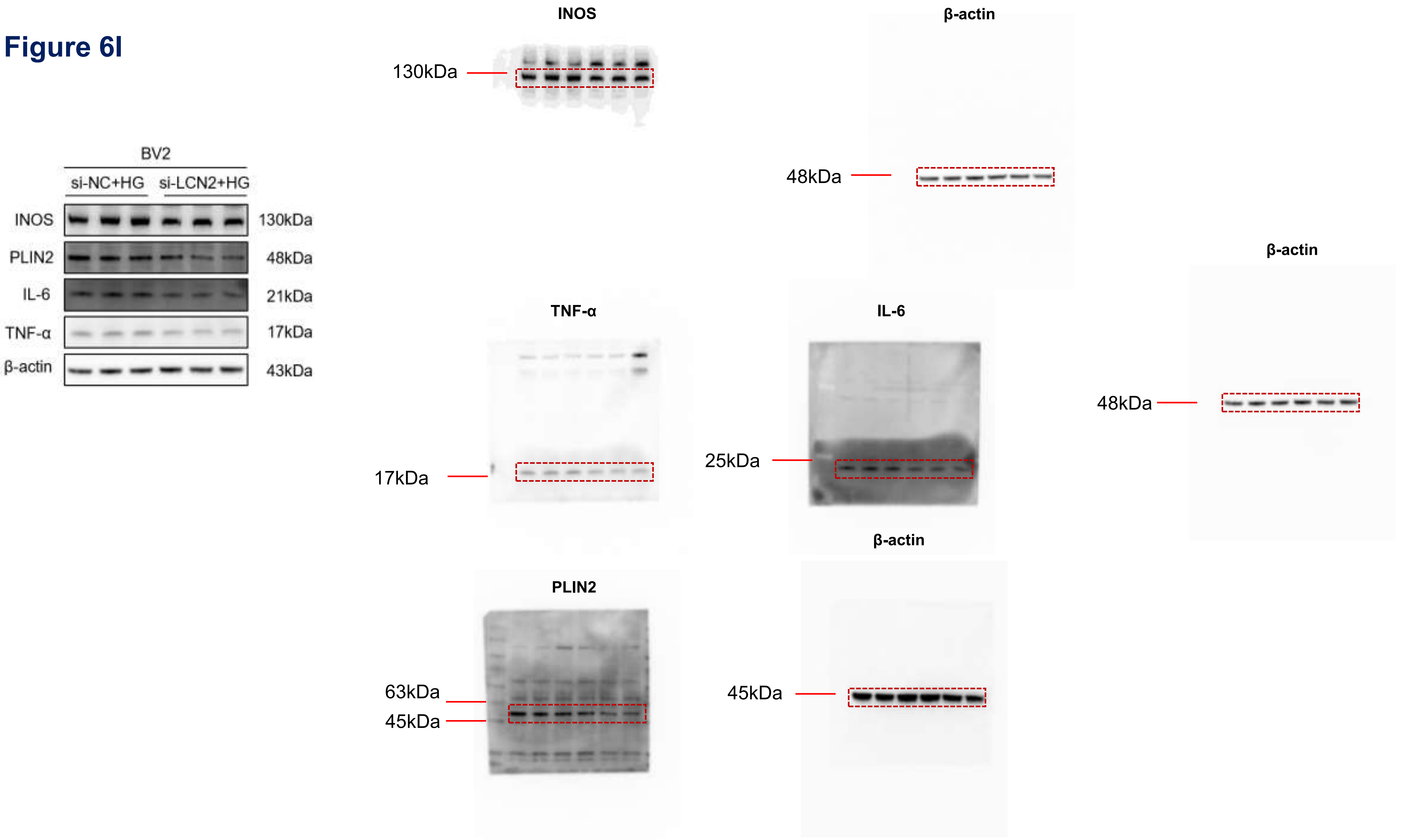

BV2
si-NC+HG
si-LCN2+HG
INOS
PLIN2
IL-6
TNF-α
β-actin
130kDa
48kDa
21kDa
17kDa
43kDa
INOS
130kDa
β-actin
48kDa
TNF-α
17kDa
IL-6
25kDa
β-actin
48kDa
PLIN2
63kDa
45kDa
β-actin
45kDa

Figure 7D

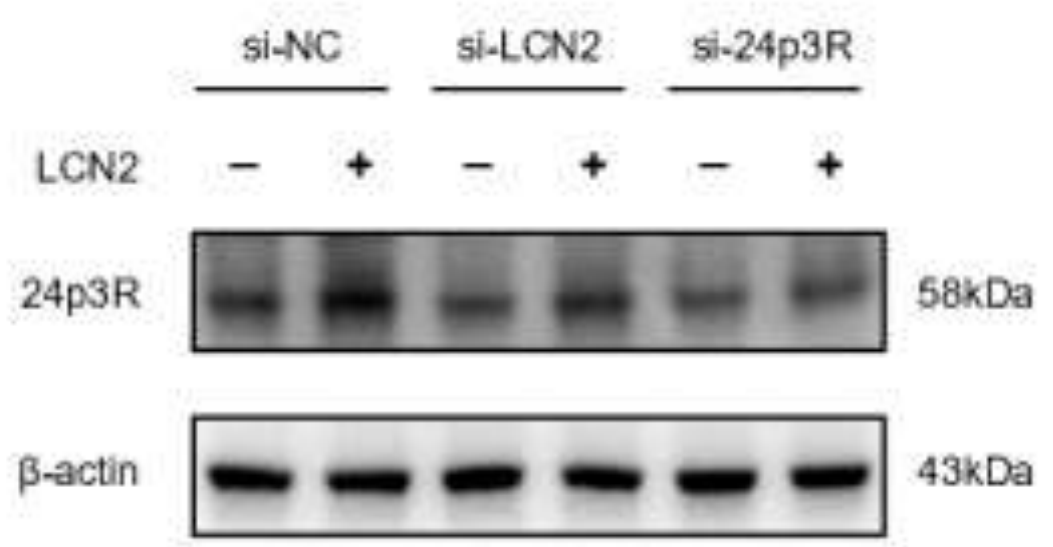

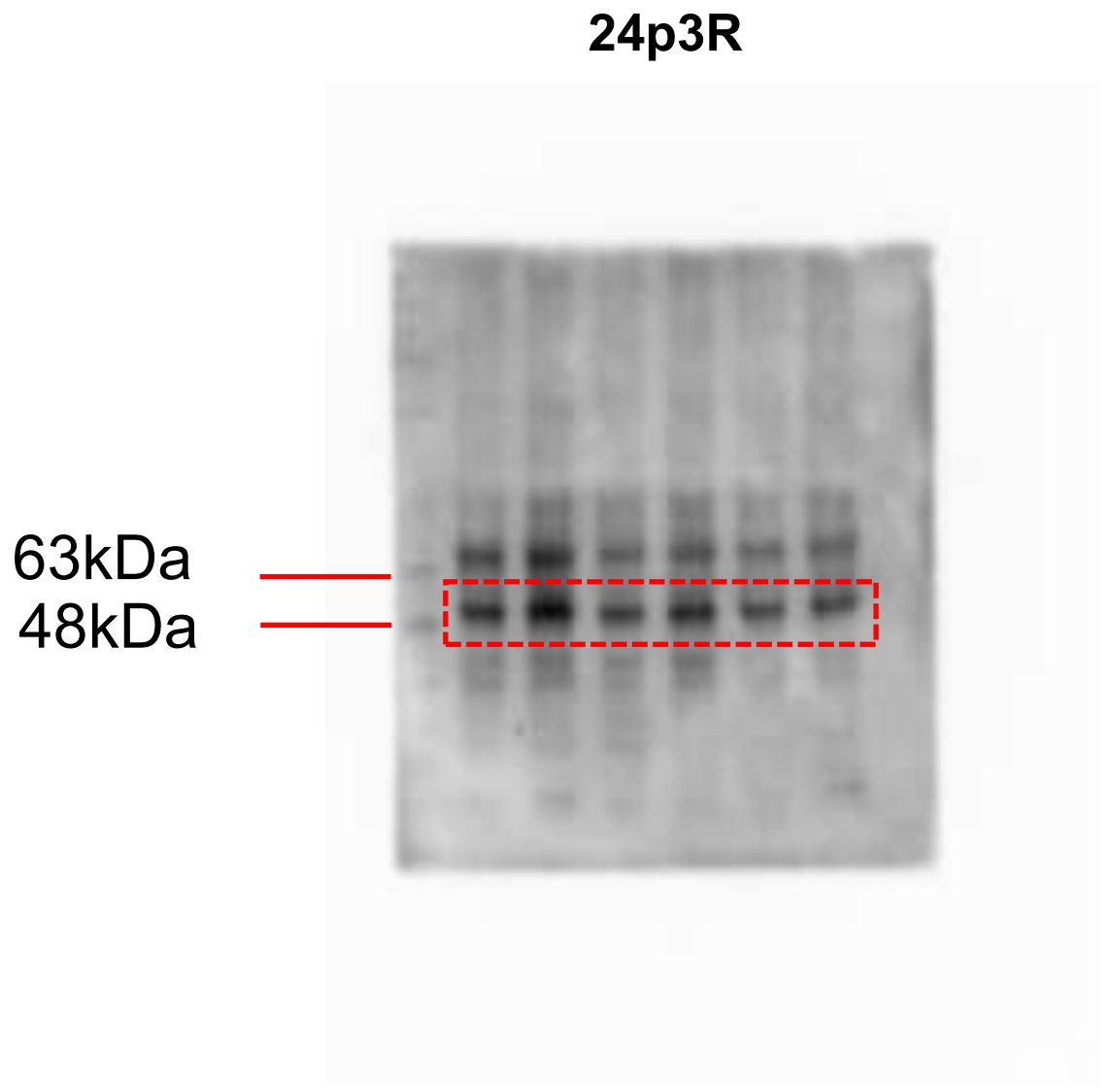

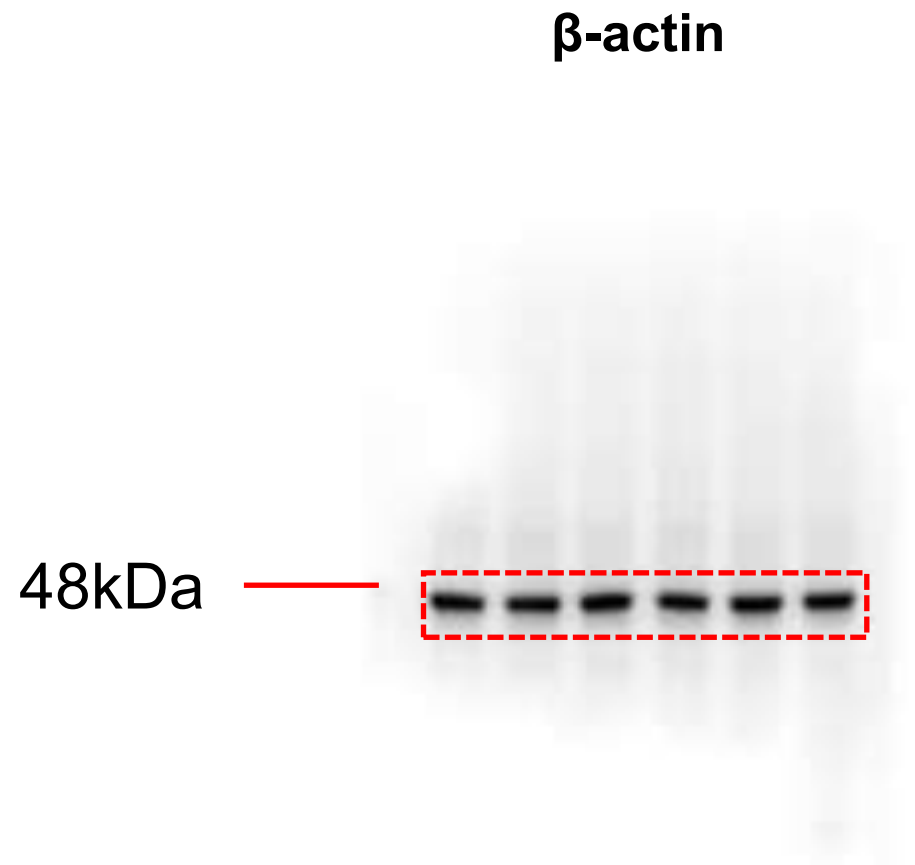

Figure 7F

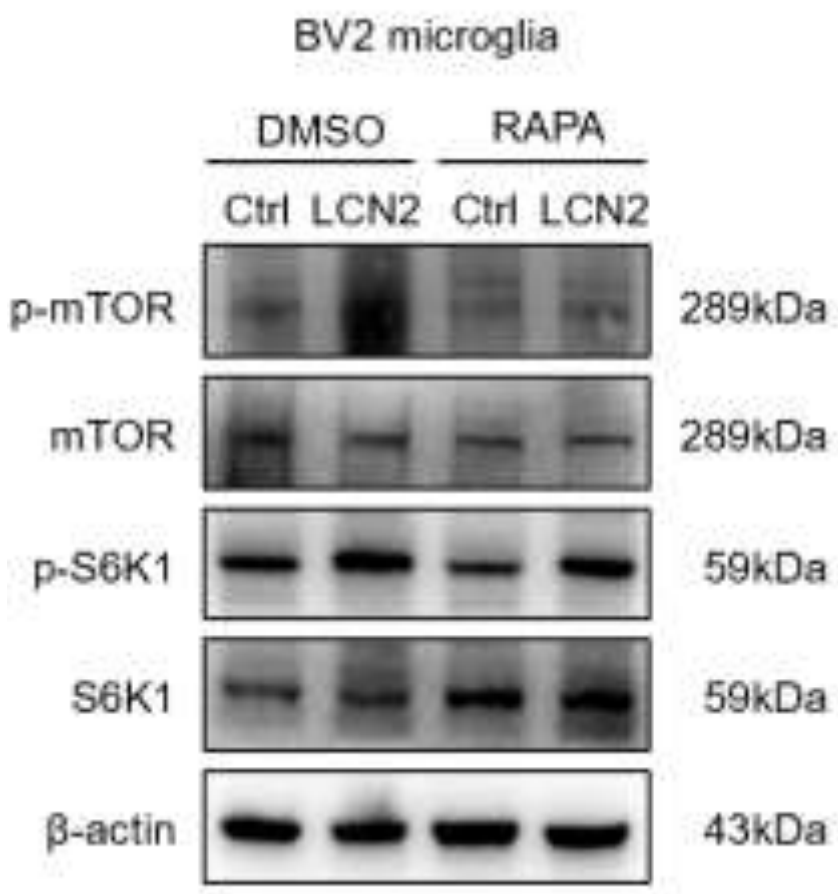

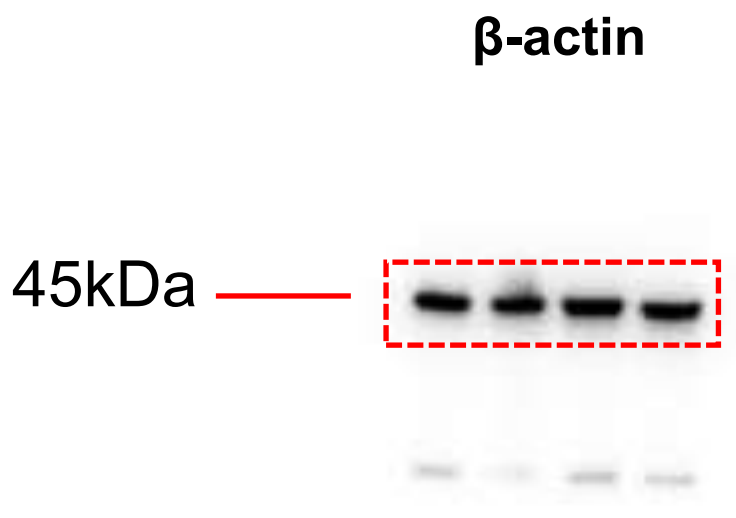

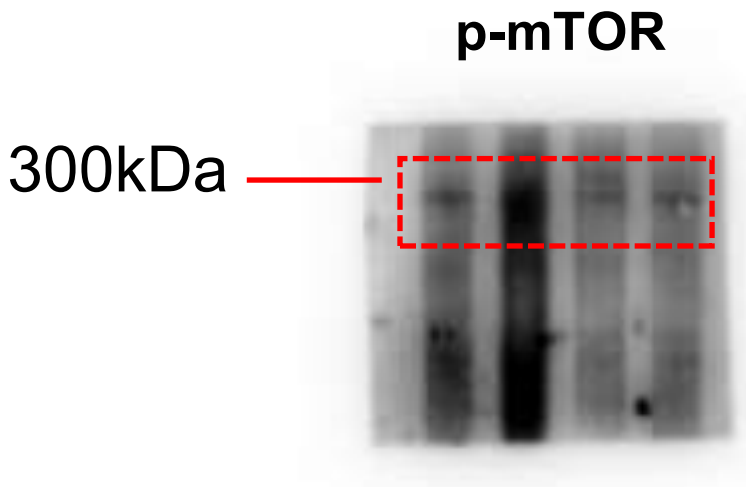

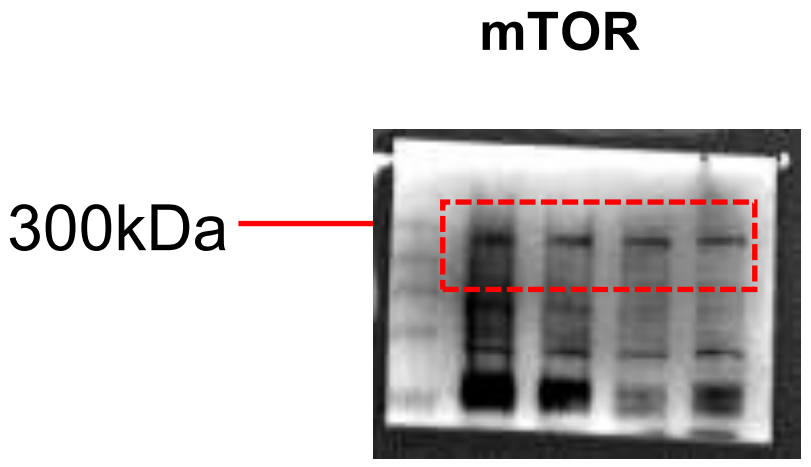

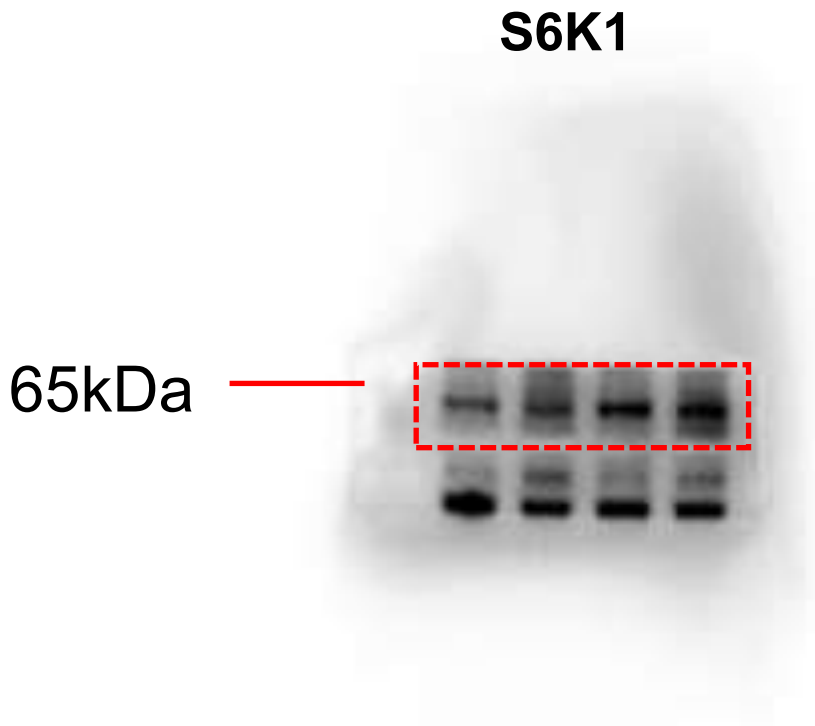

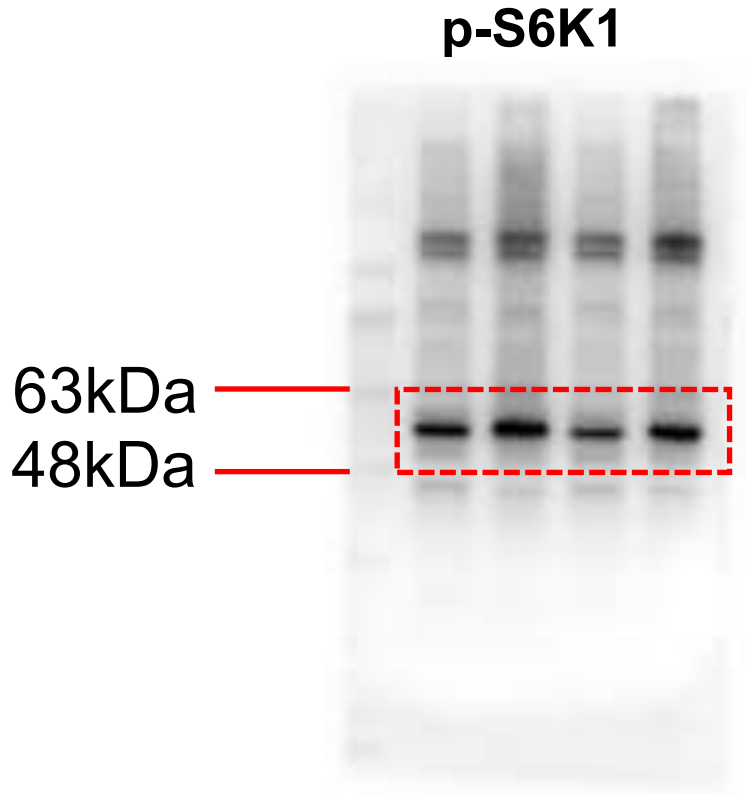

Figure 7G

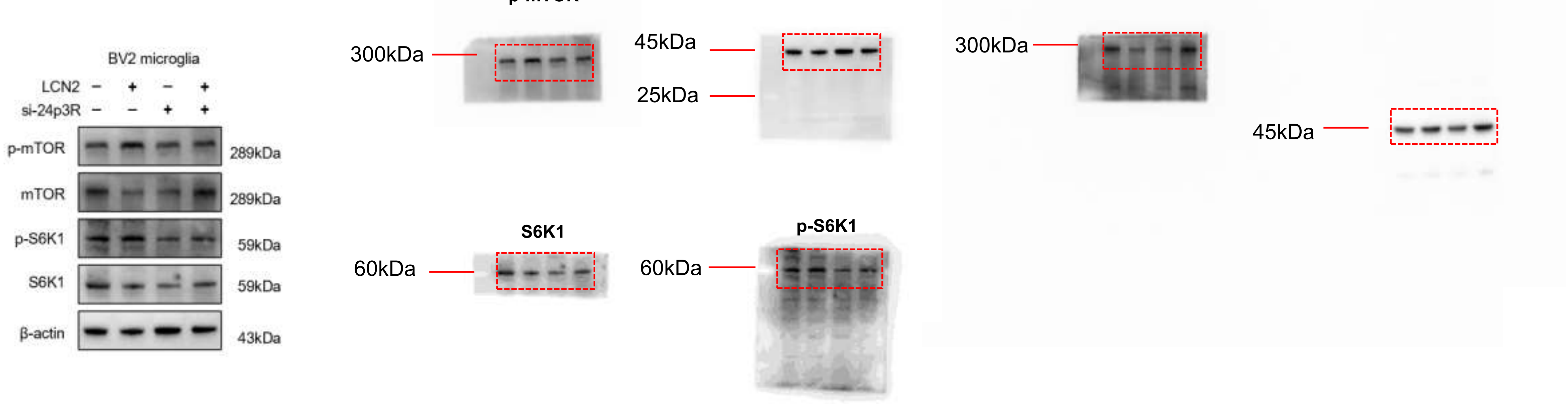

Figure 7I

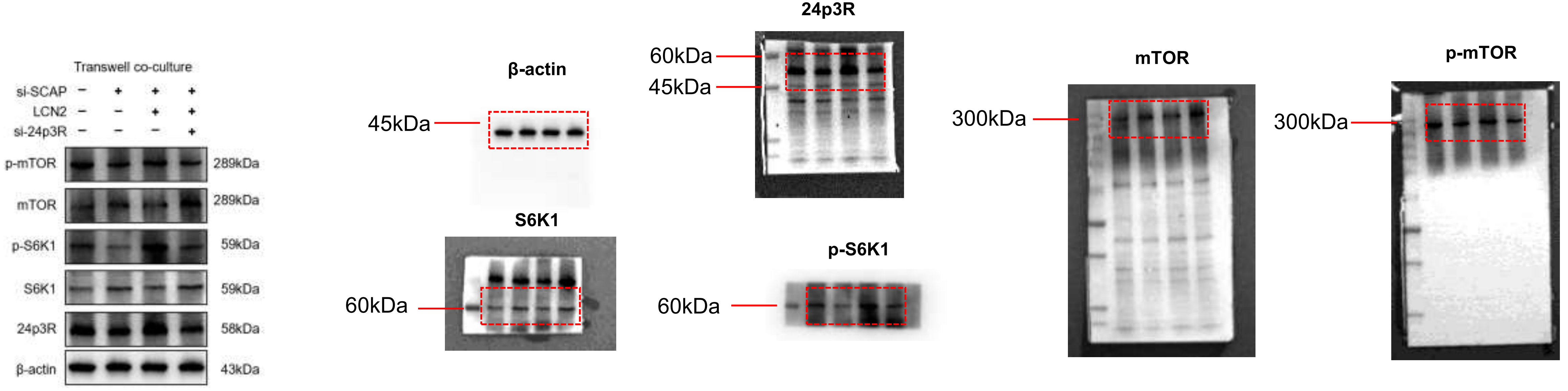

Figure 8B

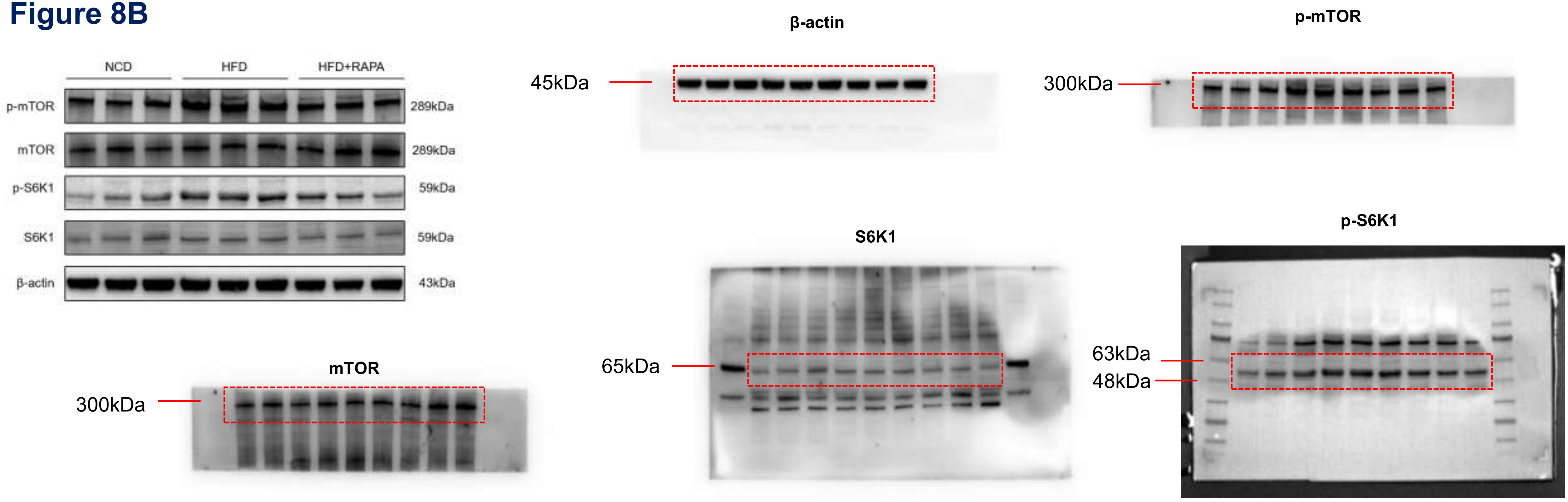

Figure S2

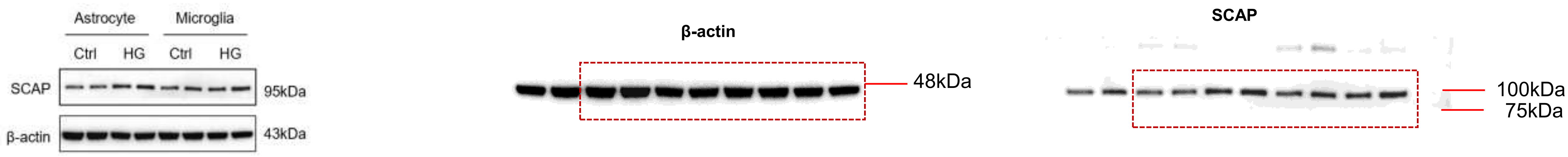

Supplement: Supplementary file 2 [file 41419_2026_9019_MOESM4_ESM.pdf]
