# Supplementary material for: Astrocytic SCAP deletion ameliorates diabetes-associated cognitive impairment by suppressing microglial neuroinflammation and lipid droplet accumulation via the LCN2–24p3R–mTOR axis
Source: Cell Death Dis. 2026 Jun 22;17(1):769. doi: 10.1038/s41419-026-09019-y (PMC13538556; doi:10.1038/s41419-026-09019-y)
Supplement: Supplementary file 3 [file 41419_2026_9019_MOESM1_ESM.docx]

**Supplementary Figure S1. Long-term high-fat diet feeding induces systemic metabolic alterations and hippocampal lipid accumulation.**

(A) Fasting blood glucose levels measured after 24 weeks of normal chow diet (NCD) or high-fat diet (HFD) feeding (n = 6 mice per group).

(B–E) Serum lipid profiles, including total cholesterol (TC), triglycerides (TG), low-density lipoprotein cholesterol (LDL-C), and high-density lipoprotein cholesterol (HDL-C), in NCD and HFD mice (n = 6 mice per group).

(F) Representative Oil Red O (ORO) staining of hippocampal sections, with quantification of ORO-positive area (%) (n = 4 mice per group). Scale bar, 10 μm.

(G) Representative fluorescence staining of lipid droplets (LDs) in hippocampal sections, with quantification of LD-positive area (%) (n = 5 mice per group). Scale bar, 50 μm.

(H) Biochemical quantification of triglyceride (TG) levels in the hippocampus of NCD and HFD mice (n = 5 mice per group).

(I) Representative Filipin III staining of hippocampal sections co-stained with GFAP. Scale bar, 20 μm.

(J) Schematic illustration of magnetic-activated cell sorting (MACS) for the isolation of microglia (CD11b⁺) and astrocytes (ACSA-2⁺) from hippocampal tissue of NCD and HFD mice for downstream RT–qPCR analysis. Created in BioRender (https://BioRender.com/3xszvj1).

(K–L) RT–qPCR analysis of Cx3cr1 and GFAP mRNA expression in CD11b⁺ and ACSA-2⁺ cell fractions, respectively, compared with their corresponding unbound fractions (n = 3 mice per group).

All data are presented as mean ± SD. Statistical analyses were performed using unpaired two-tailed Student’s t-test. *P < 0.05, **P < 0.01, ***P < 0.001; ns, not significant.

**Supplementary Figure S2. SCAP protein expression in astrocytes and microglia under control and high-glucose conditions.**

Representative Western blot images and corresponding quantification of SCAP protein expression in primary astrocytes and microglia cultured under control (Ctrl) or high-glucose (HG) conditions. β-actin served as a loading control.

Data are presented as mean ± SEM (n = 3 independent biological replicates per group). Statistical analyses were performed using unpaired two-tailed Student’s t-test. ***P < 0.001; ns, not significant.

**Supplementary Figure S3. Effects of long-term high-fat diet feeding on learning and memory performance without obvious changes in locomotor activity.**

(A) Escape latency across five consecutive training days in the Morris water maze (MWM) in NCD and HFD mice (n = 9 mice per group).

(B–C) Probe trial performance in the MWM, including (B) percentage of path length in the target quadrant and (C) number of platform crossings (n = 9 mice per group).

(D) Spontaneous alternation percentage in the Y-maze test (n = 9 mice per group).

(E–F) Novel object recognition (NOR) test performance, including preference index at (E) 1 h delay and (F) 24 h delay (n = 9 mice per group).

(G) Representative locomotor trajectories recorded during the open field test (OFT).

(H–I) Quantitative analysis of OFT performance, including (H) distance traveled in the center zone and (I) time spent in the center zone (n = 9 mice per group).

Data are presented as mean ± SEM. Statistical analyses were performed using two-way ANOVA followed by Bonferroni’s post hoc test for MWM training curves (A), and unpaired two-tailed Student’s t-test for all other comparisons (B–F, H, I). *P < 0.05, **P < 0.01, ***P < 0.001; ns, not significant.

**Supplementary Figure S4. Astrocytic SCAP deletion does not affect metabolic, behavioral, or lipid-related parameters in HFD-fed mice.**

(A) Genotyping PCR for Aldh1l1-CreERT2; SCAP fl/fl (SCAP cKO) mice and SCAP fl/fl littermate controls.

(B) Spontaneous alternation percentage in the Y-maze across the indicated groups (n = 9 mice per group).

(C–D) Novel object recognition (NOR) test performance, including preference index at (C) 1 h delay and (D) 24 h delay across the indicated groups (n = 9 mice per group).

(E) Body weight progression during 24 weeks of HFD feeding in HFD fl/fl and HFD cKO mice (n = 9 mice per group).

(F) Intraperitoneal glucose tolerance test (IPGTT) curves and corresponding area under the curve (AUC) analysis in HFD fl/fl and HFD cKO mice (n = 8 mice per group).

(G–J) Serum lipid profiles, including (G) total cholesterol (TC), (H) triglycerides (TG), (I) low-density lipoprotein cholesterol (LDL-C), and (J) high-density lipoprotein cholesterol (HDL-C), in HFD fl/fl and HFD cKO mice (n = 5 mice per group).

(K–L) Biochemical quantification of triglyceride (TG) and total cholesterol (TC) levels in the hippocampus of HFD fl/fl and HFD cKO mice (n = 5 mice per group).

(M) Representative confocal images of hippocampal sections co-stained for GFAP and BODIPY (upper panels) or GFAP and PLIN2 (lower panels), with DAPI labeling nuclei. Quantification of BODIPY⁺ and PLIN2⁺ cells among GFAP⁺ astrocytes across the indicated groups is shown (n = 3 mice per group). Scale bars, 10 μm (upper panels) and 5 μm (lower panels).

(N) RT–qPCR analysis of ABCA1, APOE, and ABCG1 mRNA expression in MACS-isolated hippocampal astrocytes from NCD fl/fl, HFD fl/fl, and HFD cKO mice (n = 3 independent biological replicates per group).

Data are presented as mean ± SEM. Statistical analyses were performed using unpaired two-tailed Student’s t-test for two-group comparisons (G–L), one-way ANOVA followed by Tukey’s post hoc test for multi-group comparisons (B–D, M–N), and two-way ANOVA followed by Bonferroni’s post hoc test for repeated-measures or factorial analyses (E, F). *P < 0.05, **P < 0.01, ***P < 0.001; ns, not significant.

**Supplementary Figure S5. Astrocyte-specific SCAP deletion does not alter SCAP expression in hippocampal microglia or neurons.**

(A) Representative confocal images of hippocampal sections co-stained for SCAP and Iba1, with DAPI labeling nuclei. Higher-magnification views of the boxed regions are shown. Quantification of SCAP fluorescence intensity in Iba1⁺ microglia is presented (n = 4 mice per group). Scale bars, 50 μm (overview images) and 10 μm (enlarged images).

(B) Representative confocal images of hippocampal sections co-stained for SCAP and NeuN, with DAPI labeling nuclei. Individual channels are shown on the right. Quantification of SCAP fluorescence intensity in NeuN⁺ neurons is presented (n = 4 mice per group). Scale bars, 20 μm.

Data are presented as mean ± SEM. Statistical analyses were performed using unpaired two-tailed Student’s t-test. ns, not significant.

**Supplementary Figure S6. Astrocytic SCAP deletion reduces LCN2 expression and secretion under high-fat diet or high-glucose conditions.**

(A) Gene Ontology (GO) enrichment analysis of genes downregulated in hippocampal tissue from HFD-fed SCAP cKO mice compared with HFD fl/fl controls, highlighting biological processes related to inflammatory and immune responses.

(B) ELISA quantification of plasma LCN2 levels in NCD fl/fl, NCD cKO, HFD fl/fl, and HFD cKO mice (n = 5 mice per group).

(C) Representative confocal images of hippocampal sections from NCD fl/fl and HFD fl/fl mice co-stained for LCN2 (red) and NeuN (green), with DAPI labeling nuclei (blue). Quantification of LCN2 fluorescence intensity in NeuN⁺ neurons is shown (n = 4 mice per group). Scale bar, 20 μm.

(D) Representative confocal images of hippocampal sections from NCD fl/fl and HFD fl/fl mice co-stained for LCN2 (red) and Iba1 (green), with DAPI labeling nuclei (blue). Split-channel images are shown to better visualize LCN2 signals. Quantification of LCN2 fluorescence intensity in Iba1⁺ microglia is presented (n = 4 mice per group). Scale bar, 20 μm.

(E) Representative confocal images of hippocampal sections from HFD fl/fl and HFD cKO mice co-stained for GFAP (green) and LCN2 (red), with DAPI labeling nuclei (blue). Quantification of LCN2 fluorescence intensity in GFAP⁺ astrocytes is shown (n = 3 mice per group). Scale bar, 20 μm.

(F) ELISA quantification of LCN2 levels in astrocyte-conditioned medium (ACM) under the indicated conditions, including control (Con), high-glucose (HG), si-SCAP, and si-LCN2 treatments (n = 3 independent biological replicates per group).

(G) Reciprocal co-immunoprecipitation (Co-IP) analysis in primary astrocytes cultured under high-glucose conditions, with immunoblot detection of SCAP and LCN2.

Data are presented as mean ± SEM. Statistical analyses were performed using unpaired two-tailed Student’s t-test (C–E) or one-way ANOVA followed by Tukey’s post hoc test (B, F). *P < 0.05, **P < 0.01, ***P < 0.001; ns, not significant.

**Supplementary Figure S7. CUT&RUN–qPCR analysis of p65 enrichment at the LCN2 promoter under the indicated conditions.**

Primary astrocytes were cultured under normal-glucose (NG) or high-glucose (HG, 33 mM) conditions and transfected with empty vector (Vector) or SCAP overexpression plasmid (SCAP OE). CUT&RUN followed by qPCR was performed to assess p65 enrichment at the LCN2 promoter. Relative enrichment levels were normalized to input and expressed as fold change relative to the Vector + NG group (n = 3 independent biological replicates per group).

Data are presented as mean ± SEM. Statistical analyses were performed using one-way ANOVA followed by Tukey’s post hoc test. *P < 0.05, ***P < 0.001.

**Supplementary Figure S8. Astrocyte-derived LCN2 mediates microglial activation and lipid accumulation under high-glucose conditions.**

(A) Cell viability of primary microglia following 24-h treatment with rLCN2 at the indicated concentrations (0, 500, 1000, and 2000 ng/mL) (n = 5 independent biological replicates per group).

(B) Representative immunofluorescence images of hippocampal sections co-stained for Iba1 and GFAP, with DAPI labeling nuclei, in NCD and HFD mice. Higher-magnification views of the boxed regions are shown. Line-scan fluorescence intensity profiles and pie charts summarizing Iba1⁺/GFAP⁺ signal colocalization are shown below (n = 4 mice per group). Scale bars, 40 μm (overview images) and 5 μm (enlarged images).

(C) Representative confocal images of BODIPY staining in BV2 microglia co-cultured with astrocytes transfected with si-NC, si-SCAP, or si-LCN2 under high-glucose conditions, with quantification of BODIPY-positive lipid droplet accumulation (n = 3 independent biological replicates per group). Scale bar, 5 μm.

(D–E) Representative confocal images and quantification of CD68 (D) and IL-1β (E) fluorescence intensity in BV2 microglia following co-culture with astrocytes transfected with si-NC or si-SCAP under high-glucose conditions (n = 3 independent biological replicates per group). Scale bar, 10 μm.

(F–G) Representative confocal images and quantification of iNOS (F) and IL-6 (G) fluorescence intensity in BV2 microglia following co-culture with astrocytes transfected with si-NC or si-LCN2 under high-glucose conditions (n = 3 independent biological replicates per group). Scale bar, 10 μm.

Data are presented as mean ± SEM. Statistical significance was determined using one-way ANOVA followed by Tukey’s post hoc test (A, C) or unpaired two-tailed Student’s t-test (D–G). *P < 0.05, **P < 0.01, ***P < 0.001; ns, not significant.

**Supplementary Figure S9. Selective upregulation of hippocampal 24p3R, but not MC4R, in HFD mice with unchanged astrocytic expression.**

(A) RT-qPCR analysis of relative mRNA expression of MC4R and 24p3R in the hippocampus of NCD and HFD mice (n = 3 mice per group).

(B) RT-qPCR analysis of 24p3R mRNA expression in MACS-isolated astrocytes from NCD and HFD mice (n = 3 mice per group).

(C) Representative confocal images of hippocampal sections co-stained for GFAP (green), 24p3R (red), and DAPI (blue) in NCD and HFD mice. Enlarged views of the boxed regions are shown. Quantification of 24p3R fluorescence intensity within GFAP⁺ astrocytes is shown on the right (n = 5 mice per group). Scale bars, 50 μm.

Data are presented as mean ± SEM. Statistical significance was determined using an unpaired two-tailed Student’s t-test. *P < 0.05, **P < 0.01, ***P < 0.001; ns, not significant.

**Supplementary Figure S10. LCN2 induces lipid metabolic reprogramming in BV2 microglia revealed by untargeted metabolomics.**

(A) Volcano plot of differentially abundant metabolites identified by untargeted LC–MS/MS metabolomics in BV2 microglia treated with control or recombinant LCN2 (rLCN2), based on fold change and statistical significance thresholds (n = 6 samples per group).

(B) Heatmap of differentially abundant metabolites identified by untargeted LC–MS/MS metabolomics in BV2 microglia treated with control or rLCN2 (n = 6 samples per group).

(C–G) Boxplots of representative glycerophospholipid-related metabolites in BV2 microglia treated with control or rLCN2, including (C) PC(0:0/18:1), (D) PC(18:2/0:0), (E) PE(22:0/13:0), (F) PA(15:0/0:0), and (G) PC(14:1/0:0) (n = 6 samples per group).

(H) KEGG pathway enrichment analysis of differential metabolites revealed significant enrichment in pathways related to amino acid metabolism, lipid metabolism, and cellular signaling, including the mTOR signaling pathway.

Data are presented as mean ± SEM. Statistical significance for (C–G) was determined using unpaired two-tailed Student’s t-test. For metabolomics analyses (A, B, H), statistical methods are described in the Methods section. *P < 0.05, **P < 0.01, ***P < 0.001; ns, not significant.

**Supplementary Figure S11. LCN2-induced microglial activation and lipid droplet accumulation are attenuated by pharmacological inhibition of mTOR.**

(A) RT-qPCR analysis of pro- and anti-inflammatory gene expression, including IL-6, IL-1β, TNF-α, IL-10, IL-4, iNOS, and Arg1, in BV2 microglia treated with vehicle control (Ctrl), rapamycin (RAPA), recombinant LCN2 (rLCN2), or rLCN2 + RAPA (n = 3 independent biological replicates per group).

(B) Representative confocal images of lipid droplets (BODIPY staining, green) with DAPI (blue) labeling nuclei in BV2 microglia treated with Ctrl, RAPA, rLCN2, or rLCN2 + RAPA. Quantification of BODIPY-positive cells is shown on the right (n = 6 independent biological replicates per group). Scale bar, 5 μm.

Data are presented as mean ± SEM. Statistical significance was determined using one-way ANOVA followed by Tukey’s post hoc test. *P < 0.05, **P < 0.01, ***P < 0.001; ns, not significant.
