# Supplementary material for: Astrocytic SCAP deletion ameliorates diabetes-associated cognitive impairment by suppressing microglial neuroinflammation and lipid droplet accumulation via the LCN2–24p3R–mTOR axis
Source: Cell Death Dis. 2026 Jun 22;17(1):769. doi: 10.1038/s41419-026-09019-y (PMC13538556; doi:10.1038/s41419-026-09019-y)
Supplement: Supplementary file 4 [file 41419_2026_9019_MOESM2_ESM.docx]

| **siRNA** | **Sequence (5′–3′)** |
| --- | --- |
| si-NC sense | UUCUCCGAACGUGUCACGUTT |
| si-NC antisense | ACGUGACACGUUCGGAGAATT |
| si-SCAP sense | CCUCCUGGCAGUAGAUGUATT |
| si-SCAP antisense | UACAUCUACUGCCAGGAGGTT |
| si-LCN2 sense | GCACCAUCUAUGAGCUACATT |
| si-LCN2 antisense | UGUAGCUCAUAGAUGGUGCTT |
| si-24p3R sense | GGCGAUUUCUACAGCGAAUTT |
| si-24p3R antisense | AUUCGCUGUAGAAAUCGCCTT |

**Supplementary Table 1. Sequences of siRNA used in this study.**

**Supplementary Table 2.** **Sequences of primers for quantitative RT-PCR.**

| **Gene** | **Primer** |
| --- | --- |
| β-actin | F: CCTGAGGCTCTTTTCCAGCC  R: TAGAGGTCTTTACGGATGTCAACGT |
| TNF-α | F: CTGAACTTCGGGGTGATCGG  R: GGCTTGTCACTCGAATTTTGAGA |
| IL-6 | F: TAGTCCTTCCTACCCCAATTTCC  R: TTGGTCCTTAGCCACTCCTTC |
| IL-1β | F: GAAATGCCACCTTTTGACAGTG  R: TGGATGCTCTCATCAGGACAG |
| IL-4 | F: CGAAGAACACCACAGAGAGTGAGCT  R: GACTCATTCATGGTGCAGCTTATCG |
| IL-10 | F: TGCTATGCTGCCTGCTCTTA  R: ATGTTGTCCAGCTGGTCCTT |
| INOS | F: GGAGTGACGGCAAACATGACT  R: CTCCAAGCCAAAGTCCTTAGAG |
| Arg1 | F: ACATTGGCTTGCGAGACGTA  R: ATCACCTTGCCAATCCCCAG |
| SCAP | F: GCTCGTGACCACCTACATCAT  R: CCCCACTTGGACTTGACCA |
| LCN2 | F: GCAGGTGGTACGTTGTGGG  R: CTCTTGTAGCTCATAGATGGTGC |
| 24p3R | F: TTTGGCCGTCGTGGGATTG  R: GGCGCAATCAGGTAGACACC |
| MC4R | F: CCCGGACGGAGGATGCTAT  R: CGCCACGATCACTAGAATGT |
| Cx3cr1 | F: GAGTATGACGATTCTGCTGAGG  R: CAGACCGAACGTGAAGACGAG |
| GFAP | F: CTACATCGAGAAGGTCCGCT  R: CCGAGGTCCTGTGTGAGATT |
| ABCA1 | F: CGTTTCCGGGAAGTGTCCTA  R: GCTAGAGATGACAAGGAGGATGGA |
| ABCG1 | F: CCCCTGAGGCTTCTACACAA  R: ATCACGGGGTCCTTGGTTAG |
| APOE | F: CCTCAAGAGCTGGTTCGAG  R: TCGGCGTTCAGTGATTGTC |

**Supplementary Table 3.** **Sequences of primers for CUT&RUN-qPCR.**

| **Gene** | **Primer** |
| --- | --- |
| LCN2-1 | F: CTTGGCACAGGGTGAAATGC  R: TGCTGGGCAGCCAATGTAAT |
| LCN2-2 | F: CCTGTTGCTCAACCTTGCAC  R: CCAAGTCCAGGAAGCCATGA |

**Supplementary Table 4. List of antibody used in the experiments.**

| **Reagent** | **Source** | **Identifier** | **Application** |
| --- | --- | --- | --- |
| β-actin Antibody | Proteintech | 20536-1-AP | WB |
| SCAP Antibody | Abcam | ab190103 | WB |
| GFAP Antibody | Proteintech | 16825-1-AP | IF |
| Iba1 Antibody | HUABIO | ET1705-78 | IF |
| SCAP Antibody | Bioss | bs-3862R | IF |
| NeuN Antibody | Proteintech | 26975-1-AP | IF |
| INOS Antibody | HUABIO | HA722031 | IF, WB |
| CD206 Antibody | Proteintech | 18704-1-AP | IF |
| CD68 Antibody | Bio-Rad | MCA1957T | IF, WB |
| PLIN2 Antibody | Proteintech | 15294-1-AP | IF, WB |
| LCN2 Antibody | AiFang Biological | AF16477 | WB |
| LCN2 Antibody | R&D Systems | AF1857 | IF, IP |
| NF-κB p65 Antibody | HUABIO | HA721307 | WB |
| p-p65 Antibody | Wanleibio | WL02169 | WB |
| Lamin B1 | Wanleibio | WL01775 | WB |
| GAPDH | Proteintech | 60004-1-Ig | WB |
| IL-6 Antibody | Wanleibio | WL02841 | WB, IF |
| TNF-α Antibody | Wanleibio | WL01581 | WB, IF |
| IL-1β Antibody | Wanleibio | WL00891 | WB, IF |
| F4/80 Antibody | Elabscience | E-AB-F0995C | FC |
| CD86 Antibody | Elabscience | E-AB-F0994E | FC |
| CD206 Antibody | BioLegend | 141705 | FC |
| CD16/32 Antibody | Elabscience | E-AB-F0997A | FC |
| 24p3R Antibody | Absin | abs119942 | IF, WB |
| mTOR Antibody | Wanleibio | WL02477 | WB |
| p-mTOR Antibody | Wanleibio | WL03694 | WB |
| S6K1 Antibody | Wanleibio | WL03839 | WB |
| p-S6K1 Antibody | Wanleibio | WL04213 | WB |
| SCAP Antibody | Proteintech | 12266-1-AP | IP |
